# Cytotoxicity, Antimicrobial, and *In Silico* Studies of Secondary Metabolites From *Aspergillus sp*. Isolated From *Tecoma stans* (L.) Juss. Ex Kunth Leaves

**DOI:** 10.3389/fchem.2021.760083

**Published:** 2021-10-13

**Authors:** Heba E. Elsayed, Reem A. Kamel, Reham R. Ibrahim, Ahmed S. Abdel-Razek, Mohamed A. Shaaban, Marcel Frese, Norbert Sewald, Hassan Y. Ebrahim, Fatma A. Moharram

**Affiliations:** ^1^ Pharmacognosy Department, Faculty of Pharmacy, Helwan University, Helwan, Egypt; ^2^ Mansheyat El-Bakry General Hospital, Cairo, Egypt; ^3^ Microbial Chemistry Department, Genetic Engineering and Biotechnology Research Division, National Research Centre, Giza, Egyp; ^4^ Chemistry of Natural Compounds Department, Division of Pharmaceutical Industries, National Research Centre, Giza, Egypt; ^5^ Organic and Bio-organic Chemistry, Faculty of Chemistry, Bielefeld University, Bielefeld, Germany

**Keywords:** antimicrobial, aspergillus sp, cytotoxicity, DNA-gyrase, Hsp90, anthraquinones, xanthones

## Abstract

Endophytes are prolific producers of privileged secondary metabolites with diverse therapeutic potential, although their anticancer and antimicrobial potential still have a room for further investigation. Herein, seven known secondary metabolites namely, arugosin C (1), ergosterol (2), iso-emericellin (3), sterigmatocystin (4), dihydrosterigmatocystin (5), versicolorin B (6), and diorcinol (7) were isolated from the rice culture of *Aspergillus sp.* retrieved from *Tecoma stans* (L.) Juss. ex Kunth leaves. Their anticancer and antimicrobial activities were evaluated in MTT and agar well diffusion assays, respectively. The cytotoxicity results showed that metabolite **3** displayed the best viability inhibition on the MCF-7 breast cancer cells with IC_50_ = 225.21 µM, while **5** on the HepG2 hepatocellular carcinoma cells with IC_50_ = 161.81 µM. **5** demonstrated a 60% apoptotic mode of cell death which is virtually correlated to its high docking affinity to Hsp90 ATP binding cleft (binding score −8.4 Kcal/mol). On the other side, metabolites **4** and **5** displayed promising antimicrobial activity especially on *Pseudomonas aeruginosa* with MIC = 125 μg/ml. The observed effect may be likely related to their excellent *in silico* inhibition of the bacterial DNA-gyrase kinase domain (binding score −10.28 Kcal/mol). To the best of our knowledge, this study is the first to report the promising cytotoxic and antibacterial activities of metabolites 3, 4, and 5 which needs further investigation and renovation to therapeutic leads.

## Introduction

Terrestrial plants are colonized by an infinite number of micro-organisms, denoted as endophytes (Uzma et al., 2018). Endophytes are often bacteria or fungi that inhabit the internal plant tissues in a symbiotic relationship (Uzma et al., 2018). Such special co-existence afforded unique, structurally diverse metabolites. Some of which demonstrate potential biological activities as antimicrobial, antioxidant, anticancer, immunosuppressive, and anti-inflammatory (Uzma et al., 2018; Keshri et al., 2021). Among the renowned fungal endophytes, is *Aspergillus* (Vadlapudi et al., 2017) which is considered a ubiquitous genus in the fungal kingdom and constitute near 185 species. Most species are characterized by their ability to synthesize heterogeneous secondary metabolites just as polyketides, xanthones, quinones, terpenoids, and alkaloids (Keshri et al., 2021) which are renowned by their profound impact on modern medicine (Newman and Cragg, 2007). Hence, insights are directed to endophytes as alternate sources for the discovery of potent antimicrobial and anticancer agents.

Cancer is a heterogeneous disease, with an impaired cellular function that grows beyond its usual boundaries (Gutschner and Diederichs, 2012). It was estimated as one of the leading causes of death worldwide thus, it exerts an enormous burden on the health systems (McGuire, 2015; Examinati et al., 2018). According to statistical evidence, hepatocellular carcinoma (HC) ranked the sixth most diagnosed cancer and the fourth leading cause of cancer mortality worldwide (Dasgupta et al., 2020). In addition, a comprehensive review has been recently published by Rashed and co-workers who discuss the critical situation of HC in Egypt as it ranked the fourth among other cancers in terms of mortality rate (Rashed et al., 2020). On the other hand, breast cancer is the most common type of cancer in women and the first cause of cancer death among them (Bray et al., 2018). In Egypt, it constitutes 33% of female cancer cases and more than 22,000 new cases are diagnosed with it each year (Ibrahim et al., 2020). This is expected to rise exponentially over the next years given the enlarging population, changes in the population pyramid, and adopting the westernized lifestyle. Among the major challenges associated with the currently available cancer treatments is their toxicity, resistance, and selectivity (Zugazagoitia et al., 2016). Accordingly, these drawbacks have motivated our research group for the *in vitro* screening of natural products isolated from various natural sources on the MCF-7 breast cancer and the hepatocellular HepG2 adenocarcinoma cells as representative in-house available cell lines for the screening protocol and discovery of promising bioactive hits.

On the other hand, the increasing prevalence of pathogenic bacteria that are resistant to currently available antibiotics, just as *Pseudomonas aeruginosa*, represents an alarming threat to public health (Tajkarimi et al., 2010; Breijyeh, et al., 2020). Hence, there is an interest in the discovery of new natural antimicrobial agents aimed at opposing these pathogens and reducing their acquired resistance.

*Tecoma stans* (L.) Juss. ex Kunth (family Bignoniaceae) is cultivated in Egypt for ornamental purposes, although it is characterized by the presence of an array of secondary metabolites correlated to diverse biological activities (Bhat, 2019). Hence, we hypothesized that the endophytes inhabiting this plant might produce various bio-active metabolites. In this regard, the study herein aimed at the investigation of the ethyl acetate extract of the endophytic fungus, *Aspergillus sp.* isolated from *T. stans* leaves for the first time, in an attempt, to discover cytotoxic and antimicrobial hits. In addition, a molecular docking study was conducted to suggest the molecular targets involved in both activities.

## Materials and Methods

### General Experimental Procedures

Silica gel 60 (Merck, Darmstadt, Germany) and Sephadex LH-20 (Sigma-Aldrich, Steinheim, Germany), as well as pre-coated silica gel 60 F_254_ plates (Merck, Darmstadt, Germany), were used for column chromatography (CC) and thin-layer chromatography (TLC), respectively. ^1^H and ^13^C NMR data of isolated metabolites were measured on Bruker Avance (Bruker, Rheinstetten, Germany) at 400, 500, and 600 MHz for ^1^H NMR, while 100, 125, and 150 MHz for ^13^C NMR. The NMR data were represented as δ ppm values relative to the internal reference TMS. ESI-MS spectra were recorded on a Micromass AC-TOF spectrometer (Micromass, Agilent Technologies 1,200 series, Waldbronn, Germany). UV data were reported on a UV-, V-630 BIO spectrophotometer (JASCO, United States). Specific optical rotation was measured on Ap-300 Automatic polarimeter (ATAGO Co. Ltd., United States). All chemicals and solvents used were of analytical grade.

### Fungal Isolation and Fermentation

*T. stans* leaves were collected from El Orman Botanical Garden, Giza, Egypt during February 2017. The plant was authenticated by Dr. Mohammed El-Gibaly, Lecturer of Taxonomy and Consultant for Central Administration of Plantation and Environment, Giza, Egypt. A voucher specimen (28Tst/2017) was kept at the Pharmacognosy Department herbarium, Faculty of Pharmacy, Helwan University, Cairo, Egypt. The plant endophytes were retrieved, cultured, and purified according to previously stated procedure by Quambusch et al. (2014). Briefly, the leaves were cut into small pieces, washed with sterilized distilled water and 70% aqueous ethanol (2 × 2 min). Thereafter, the inner tissue was cleaved into small segments and located onto fresh potato dextrose agar (PDA) media (Innis et al., 2012). The plates were incubated for 3–6 weeks at 25°C and the resulted colonies were transferred to fresh PDA media and kept at 40°C. Further pure strains were isolated by repeated, successive inoculation.

### Identification of the Isolated Fungal Strain

The partial sequence of large subunit ribosomal RNA (18S rDNA) was used for the identification of the fungal isolates. ABT DNA mini extraction kit (Applied Biotechnology Co. Ltd., Egypt) was used for extraction of the fungus total genomic DNA from an *in vitro* culture after applying the guidelines. the nuclear ribosomal internal transcribed spacers (ITS rDNA) were amplified as single fragments by Polymerase Chain Reaction (PCR, Innis et al., 2012). Hot starTaq Master Mix Kit (Bio-Rad, Hercules, CA, United States) was mixed with a pair of primers ITS1 (sequence: 50-TCC GTA GGT GAA CCT GCG G-3′) and ITS4 (sequence 50-TCC TCC GCT TAT TGA TAT GC-3′) and DNA template in the thermal cycle as mentioned in the manufacturer protocol. The PCR product was isolated from the gel slice by the Gen Elute™ Gel extraction kit. The corresponding ITS-rDNA sequence of the fungus was then used for similarity analysis using the Blast N algorithm against the public database at the National Centre for Biotechnology Information (NCBI; http://www.ncbi.nlm.nih.gov). The sequence data were deposited at Gene Bank with accession number MN148642. The phylogenetic trees were constructed using Molecular Evolutionary Genetics Analysis (MEGA) version 10.0.5.

### Fungal Mass Cultivation

Fungi mass was transferred from the PDA media to sterile distilled water (5 ml), then inoculated into 100 ml of yeast/malt extract broth at 25°C and served as the seed culture. Three days later, about 5 ml of the seed culture was grown in Erlenmeyer flasks (5 × 1L) containing solid rice medium (100 g rice and 150 ml distilled water) for 14 days at 25°C under static conditions.

### Extraction and Isolation of the Secondary Metabolites

After the end of the incubation period, the rice cultures were mixed with ethyl acetate (EtOAc, 300 ml) overnight. The extraction was repeated at least three times, followed by filtration to remove the mycelia. The pooled extracts were evaporated under vacuum to afford 0.757 g. The residue was dissolved in a 15 mL dichloromethane (DCM)/methanol (MeOH) mixture (95:5 v/v), mixed with 2 g silica then subjected to fractionation using silica gel column chromatography (CC, 120 g, 40 × 3 cm). The column was eluted with 100% DCM followed by gradually increasing the polarity with MeOH (1% till 50% MeOH) to give nineteen fractions each of 0.3 L. All fractions were collected based on their thin layer chromatography (TLC) similarity pattern after spraying the chromatograms with *p*-anisaldehyde/sulfuric acid as well as their UV activity. Five major collective fractions (I–V) were finally obtained. Fr. II (100% DCM, 0.2 g) was subjected to fractionation on Sephadex LH-20 CC using DCM/MeOH (50:50 v/v) as an eluent to afford three subfractions. Each subfraction was purified using Sephadex LH-20 CC using DCM: MeOH (50:50 v/v) as an eluent to yield pure compounds **1** (13.1 mg), **2** (9.2 mg), and **3** (10.0 mg). Moreover, Fr. III (100% DCM, 0.11 g) was fractionated on Sephadex LH-20 CC (DCM: MeOH, 50:50 v/v) to afford two subfractions 1) and 2). Compound **4** (7.2 mg) was obtained by precipitation from subfraction 1) by the addition of excess cyclohexane. Subfraction 2) was subjected to preparative TLC (PTLC) using cyclohexane: DCM (80:20 v/v) mixtures, followed by Sephadex LH-20 CC eluted with DCM:MeOH 50:50 v/v to yield pure compound **5** (10 mg). Concerning Fr. IV (0.1 g, DCM: MeOH. 99:1, v/v), it was subjected to (PTLC) using DCM: MeOH (97:3 v/v) to provide crude samples of **6** and **7**. The samples were purified using Sephadex LH-20 CC eluted with DCM: MeOH (50:50 v/v) to afford pure compounds **6** (12 mg) and **7** (8 mg). Lastly, fraction V (0.16 g, DCM: MeOH. 98:2, v/v) remained unexplored due to its complexity (Supplementary Scheme S1). The purity and identity of the isolated compounds were established according to their colours under UV light, spraying with p-anisaldehyde/sulfuric acid and heating the chromatograms.

### Cell Lines and Culture Conditions

The hepatocellular carcinoma (HepG2) and Human breast adenocarcinoma (MCF-7) cell lines were purchased from the American Type Culture Collection (ATCC, Manassas, VA, United States). Fetal bovine serum, L-glutamine, penicillin, streptomycin G, amphotericin B, trypsin/EDTA were obtained from Sigma-Aldrich, United States while, Dulbecco’s Modified Eagle’s Medium (DMEM) was supplied from Cambrex BioScience (Copenhagen, Denmark). All cells were cultured in DMEM supplemented with 100 U/mL penicillin, 10% fetal bovine serum, 100 U/mL streptomycin, and 250 ng/ml amphotericin B and maintained in humidified air containing 5% CO_2_ at 37°C.

### MTT Cytotoxicity Assay

Microculture tetrazolium assay (MTT) was used for assessing the cytotoxic activity compared to DMSO as negative control (Vistica et al., 1991). Briefly, cells were treated with the extract or compounds **1, 3–7** (20 µL medium/well), prepared in DMSO, at least in four different concentrations. The plate was incubated at 37°C in humidified 5% CO_2_ atmosphere for 24 h, then 40 µL of MTT solution/well was added and re-incubation was continued for 4 h. The crystals were solubilized using DMSO and the absorbances were measured at λ_570_ nm using an ELISA microplate reader (Sunrise, TECAN Inc., CA, United States). The experiments were repeated in triplicate for each concentration. % Cell viability was calculated from the following equation.

% Cell viability = [(Abs. of tested sample–Abs. of negative control/Abs. of negative control) × 100].

The relation between drug concentration and surviving cells is plotted to get the survival curve of each cell line. The IC_50_ ± SD was calculated from the equation of the dose-response curve using linear regression analysis implemented to GraphPad prism 9.2.0.

### Mode of Cell Death

The conventional Ethidium bromide/acridine orange (EB/AO) method was adopted to investigate the mode of cell death induced by the most active compounds on MCF-7 and HepG2 cancerous cells (Ribble et al., 2005; Cohen, 1993). Briefly, about 1 × 10^4^ cells were treated separately with 50% of the IC_50_ on cell culture slides (SPL, South Korea) for 24 h. Thereafter, the slides were washed with phosphate buffer saline and stained with EB/AO for 10 min in the dark followed by examination with fluorescence microscope (Axio Imager Z2, Zeiss, Germany) using ZEN 11 blue edition software (Zeiss, Germany). The percentages of live, apoptotic, and necrotic cells were calculated for each sample concentration at its respective cell line on basis of 1,000 cells/sample (*n* = 3).

### Standard Microbes, Antibiotics, and Culture Media

The stock cultures of Gram-negative bacteria, *Escherichia coli* ATCC 8739*, Klebsiella pneumoniae* ATCC 700603*, Pseudomonas aeruginosa* ATCC 9027, Gram-positive bacteria as *Staphylococcus aureus* ATCC 25923*, Streptococcus faecalis* ATCC 8043*, Listeria monocytogenes* ATCC 7644*,* and the fungus *Candida albicans* ATCC 10231 were obtained from Microlab, Institute of Research and Technology, Vellore, Tamilnadu, India. The microbiological growth media, Mueller-Hinton agar, and Mueller-Hinton broth were obtained from Oxoid, ThermoFisher Scientific (MA, United States). Positive control antibiotic 6.0 mm discs as Chloramphenicol (C30), Gentamycin (CN10), Ceftriaxone (CEC30), and Cefoxitin (FOX30) were purchased from Oxoid, ThermoFisher Scientific (MA, United States), while biological grade sterile DMSO from ThermoFisher Scientific (MA, United States).

### Antimicrobial Susceptibility Test

The agar well diffusion assay was used to determine the susceptibility of a panel of microbes to various treatments according to the Clinical and Laboratory Standards Institute (Gholizadeh et al., 2013; Wayne, 2019). Briefly, about 100 µL of 1 × 10^5^ bacterial suspensions were spread individually on the surface of Muller Hinton (MH) agar plates and 0.6 cm diameter wells were made. Each well received 50 µL of the extract or pure compounds, then the plates were kept in the refrigerator to enhance the diffusion of the treatments. The plates were incubated for 24 h under the proper temperature. The antimicrobial susceptibility was determined by measuring the diameter of developed inhibition zones in mm. The activity was compared to conventional antibiotics with different modes of action. For instance, chloramphenicol (C30), gentamycin (CN10) as protein synthesis inhibitors, whereas ceftriaxone (CEC30) and cefoxitin (FOX30) are cell wall synthesis inhibitors, while DMSO was used as a negative control.

### Determination of Minimum Inhibitory Concentrations

MIC was determined using the broth microdilution assay (Balouiri et al., 2016). Briefly, 10 mg of the ethyl acetate extract and 1 mg of pure metabolites **1 and 3–5** were dissolved in 1 ml DMSO to prepare the stock solutions. The stock solutions were diluted to 1/10 using sterile Mueller Hinton broth (MHB). On the other hand, about 100 µL of sterile MHB was added to each well of a microtiter plate followed by 150 µL of the diluted extract or tested compounds in a two-fold serial dilution manner. Ultimately, 100 µL of each microbial inoculum containing 1 × 10^5^ CFU/ml (obtical density (OD) = 0.08–0.12 at λ_max_ 625) was transferred to each well except the blank ones. The plate was incubated at 37°C for 24 h, thereafter the absorbances of the wells were recorded using an automated Elisa reader (Asys Hitech GmbH, Austria).

### Molecular Docking

The molecular docking experiment was conducted using Auto Dock Vina, M.G.L tools 1.5.7. to validate the cytotoxic activity of the isolated metabolites The X-ray crystal structure of the heat shock protein 90 (Hsp90), as proposed molecular target, was retrieved from the protein data bank (PDB code: 4YKQ; http://www.rcsb.org). The active site has been determined from the binding of the co-crystalized ligand 1-(5-ethyl-2,4-dihydroxy phenyl)-1,3-dihydro-2H-benzimidazole-2-one (CS301), while grid generation was delineated using MGL tools 1.5.7. Auto Dock Vina achieves approximately two orders of magnitude speed-up using multithreading on multi-core stations to significantly improve the accuracy of the binding mode predictions. The structures of the isolated compounds **1, 3–7** were sketched using Molecular Graphics Laboratory (MGL) tools 1.5.7. and transformed to protein data bank, partial charge (Q) and atom type (T) (pdbqt) format. The energy minimization of the enzyme, co-crystallized ligand and the isolated compounds were prepared using MGL tools 1.5.7. The docking results were visually inspected by the discovery studio 4.5 visualizer. The binding results are expressed as Auto Dock score which calculates free binding energies and iterated local search global optimization algorithm (Baxter, 1981; Abagyan et al., 1994).

On the other side, the molecular docking study for the virtual validation of the antimicrobial activity was carried out using Molecular Operating Environment (MOE, 2019.0102) software. The minimizations were performed with MOE until a root square mean deviation (RMSD) gradient of 0.1 kcal∙mol^−1^Å^−1^ with MMFF94x force field. The X-ray crystallographic structure of DNA gyrase enzyme complexed with its ligand (1-[5-[6-fluoranyl-8-(methylamino)-4-[3-(trifluoro methyl)pyrazol-1-yl]-9H-pyrido[2,3-b]indol-3-yl]pyrimidin-2-yl]cyclopropane-1-carboxylic acid, EZ6, PDB ID: 6M1J) was downloaded from the protein data bank**.** For each co-crystallized enzyme, water molecules and ligands which are not involved in the binding were removed. The protein was prepared using *Protonate 3D* protocol in MOE with default options. Triangle Matcher placement method and London dG scoring function were used for docking. The 3D molecular visualization was sketched using easy-to-use graphical interface option of the MOE platform.

## Results

**FIGURE 1:**
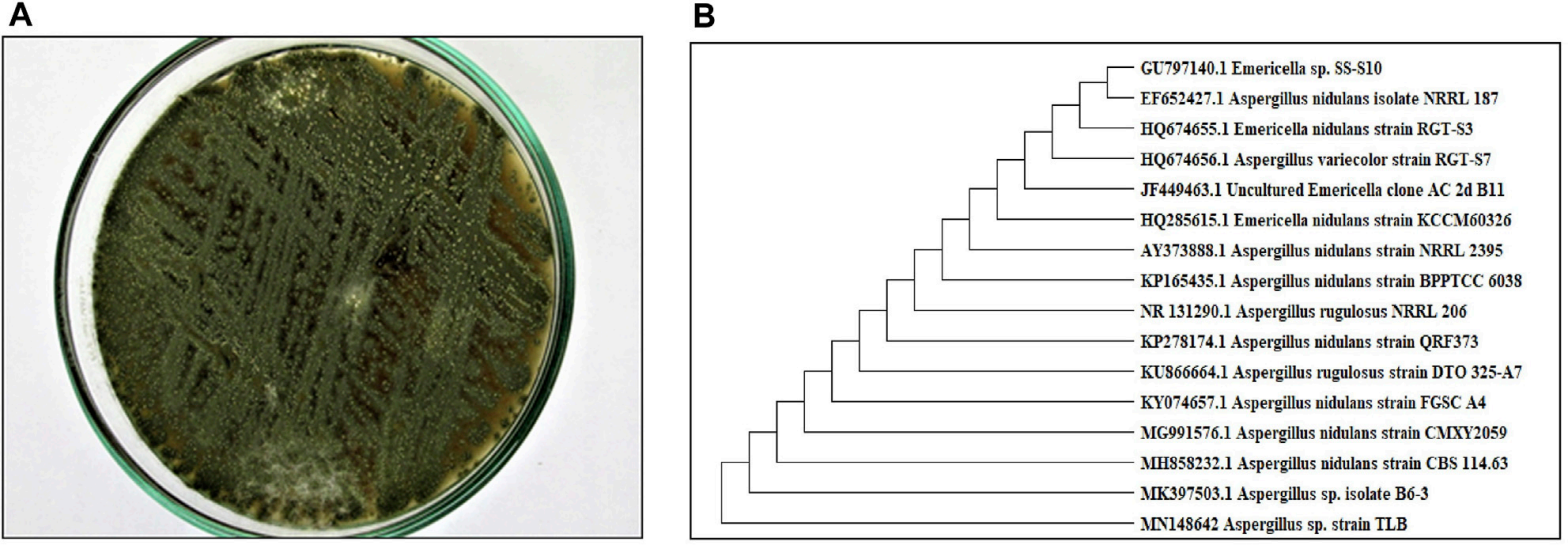
**(A)** Colony morphology of Aspergillus sp. isolated from *T. stans* leaves on potato dextrose agar (PDA) media. **(B)** Neighbor-joining phylogenetic tree of strain TLB (MN148642) based on 18 S rRNA gene sequences, showing its close relationship to *Aspergillus* species.

**FIGURE 2:**
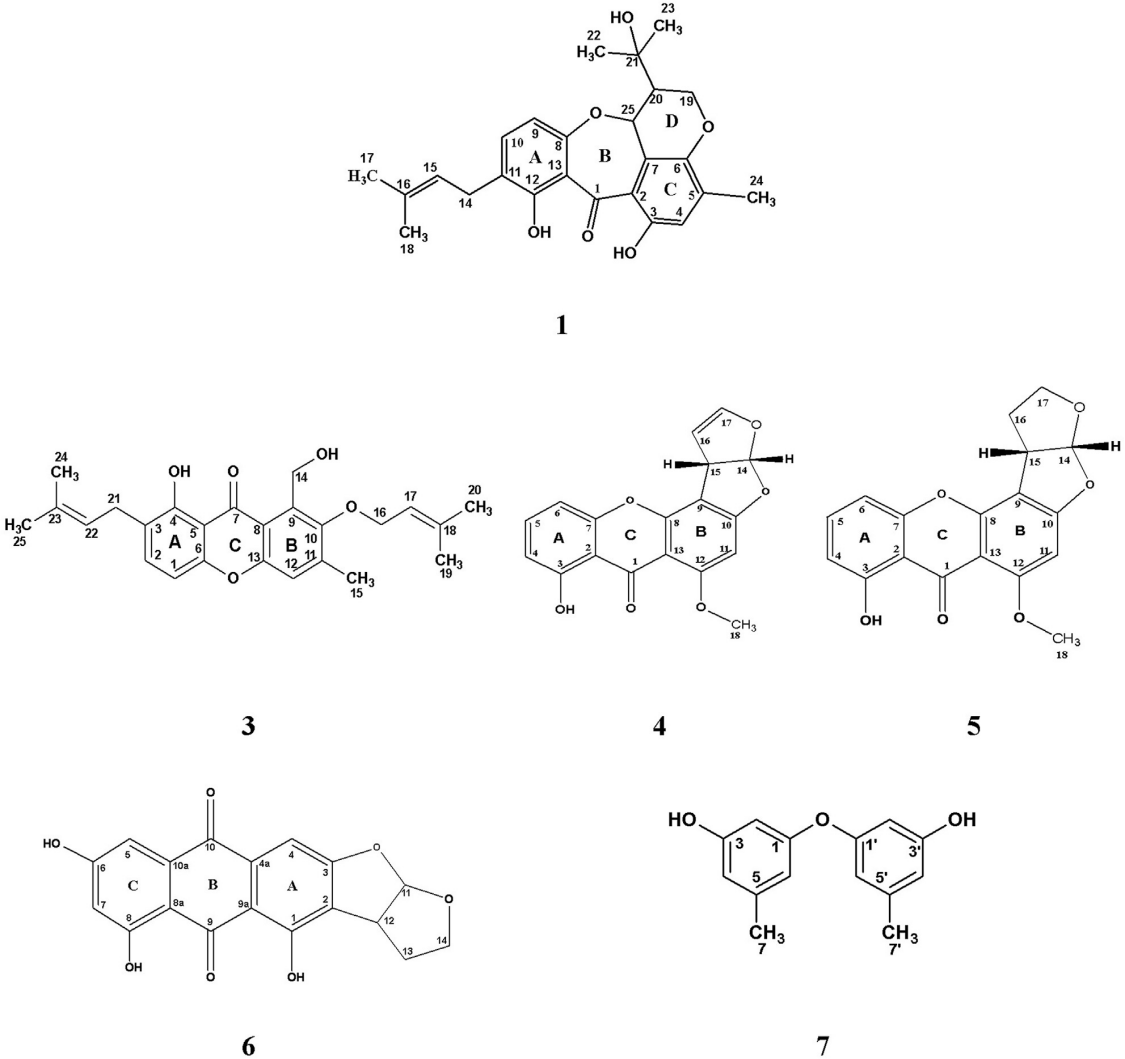
Chemical structure of isolated compounds (1, 3–7) from the ethyl acetate extract of the rice culture of *Asperigellius sp.* obtained from *T. stans* (L.) Juss. ex Kunth leaves.

A pure colony of the endophytic fungus *Aspergillus sp.* was isolated from *T. stans* leaves and characterized based on its morphologic traits just as the diameter of the colonies (42–53 mm) after 7 days of incubation on PDA medium at 25°C, the deep green abundant smooth conidia with white mycelia, and the dark brown colour shown on the reverse (Figure 1A). Moreover, the molecular phylogenic tree (Figure 1B) has been delineated with 100% similarity index (SI) to both *Aspergillus nidulans* and *Aspergillus regulosus* species, hence the isolated fugus was established as *Aspergillus sp.* The ethyl acetate extract (EtOAc) of the rice culture of the isolated fungi exerts cytotoxic potential in a dose response manner in MTT assay with IC_50_ equivalent to 186.66 ± 9.4 and 158.54 ± 7.2 µg/ml on MCF-7 and HepG2 cell lines, respectively (Table 1). Meanwhile, the extract was assessed for its ability to inhibit the growth of selected bacterial and fungal strains in agar well diffusion assay. The results declared that the extract displayed promising antibacterial activity at the maximum tested dose (10 mg/ml) against *L. monocytogenes, K. pneumonia*, *P. aeruginosa* with a noticeable zone of inhibition measured as 11, 12, and 17 mm, respectively compared to the available tested antibiotics. Interestingly, *E. coli*, *S. aureus*, *S. faecalis,* and *C. albicans* showed high resistance to the applied treatment at the proposed tested concentrations. Moreover, the MIC of the extract on the forementioned susceptible organisms was calculated as 125–1,000 µg/ml with *P. aeruginosa* being the most sensitive organism to all treatments (125 µg/ml, Table 2). Accordingly, to identify the bioactive metabolites that are responsible for the observed activities, the EtOAc extract was subjected to chromatographic fractionation with the subsequent isolation of seven known metabolites (Supplementary Scheme S1). All metabolites (Figure 2) were elucidated based on their chemical and spectroscopic data (UV, ESI/MS, 1-and 2D-NMR) and detailed discussion was accomplished in comparison to those previously stated in the literature (Supplementary Data).

**TABLE 1:** Inhibitory concentrations (IC_50_ ± SD) of the total ethyl acetate extract (µg/ml) and pure compound 1, 3–7 (µM) in MTT cytotoxicity assay on MCF-7 breast cancer and HepG2 hepatocellular carcinoma cell lines.

Compound/Extract	IC_50_ ± SD	
	MCF-7	HepG2
EtOAc extract^a^	186.66 ± 9.4	158.54 ± 7.2
1^b^	449.95 ± 11.3	697.30 ± 19.3
3^b^	225.21 ± 7.4	232.07 ± 4.9
4^b^	403.97 ± 4.1	265.20 ± 9.7
5^b^	453.57 ± 8.3	161.81 ± 5.2
6^b^	382.03 ± 7.2	476.07 ± 11.2
7^b^	577.60 ± 4.2	647.09 ± 16.5

a (µg/ml).

b (µM).

**TABLE 2:** Screening of the inhibitory effect of EtOAc extract (tested conc. 5 and 10 mg/ml) and pure compounds 1, 3–5 (tested conc. 0.5 and 1 mg/ml) against reference standards of Gram-positive, Gram-negative human pathogens, and fungi in comparison to standard tested antibiotics in agar well diffusion assay.

Strains/Conc. (mg/ml)	Tested treatments										Tested antibiotics			
	EtOAc		1		3		4		5		C	CN	CEC	FOX
	10	5	1	0.5	1	0.5	1	0.5	1	0.5	30	10	30	30
Gram + ve bacteria													
*S. aureus*	Nz	Nz	16 mm	Nz	11 mm	9 mm	11 mm	Nz	Nz	Nz	13 mm	—	—	—
*S. faecalis*	Nz	Nz	13 mm	Nz	11 mm	Nz	10 mm	Nz	Nz	Nz	9 mm	10 mm	—	—
*L. monocytogenes*	11 mm	Nz	Nz	Nz	Nz	Nz	Nz	Nz	Nz	Nz	7 mm	8 mm	—	—
Gram −ve bacteria													
*E. coli*	Nz	Nz	Nz	Nz	Nz	Nz	Nz	Nz	Nz	Nz	—	—	—	—
*K. pneumoniae*	12 mm	Nz	Nz	Nz	13 mm	Nz	Nz	Nz	Nz	Nz	12 mm	—	—	—
*P.aeruginosa*	17 mm	14 mm	14 mm	13 mm	14 mm	12 mm	15 mm	14 mm	18 mm	10 mm	9 mm	—	—	—
Fungi														
*C. albicans*	Nz	Nz	Nz	Nz	Nz	Nz	Nz	Nz	Nz	Nz	18 mm	9 mm	—	—
10% DMSO (−ve control)	Nz	Nz	Nz	Nz	Nz	Nz	Nz	Nz	Nz	Nz	—	—	—	—

C: chloramphenicol, CN: Gentamycin, CEC: Ceftriaxone, FOX: cefoxitin, Nz = No zone of inhibition was observed, - = not tested.

### Arugosin C (1)

It was obtained as yellow amorphous powder, develops dark purple color upon spraying with *p*-anisaldehyde/sulfuric acid reagent. UV (MeOH, λ_max_, nm): 309; 360 (sh). Positive mode LR-ESI-MS: *m/z* 447.3 [M + Na] ^+^. ^1^H NMR (500 MHz, CDCl_3_ *-d*) δ_H_ 13.80 (s, 12-OH), 10.63 (s, 3-OH), 7.21 (1H, d, *J* = 8.5, H-10), 6.74 (1H, s, H-4), 6.34 (1H, d, *J* = 8.5, H-9), 5.25 (1H, m, H-15), 5.01 (1H, d, *J* = 4.5, H-25), 4.30 (1H, dd, *J* = 11.5, 4.0, H-19_a_), 4.13 (1H, dd, *J* = 11.5, 7.0, H-19_b_), 3.24 (2H, d, *J* = 7.5, CH_2_-14), 2.30 (1H, dt, *J* = 7, 4.0, H-20), 2.17 (3H, s, CH_3_-24), 1.69 (3H, s, CH_3_-17), 1.65 (3H, s, CH_3_-18), 1.25 (3H, s, CH_3_-22), 1.19 (3H, s, CH_3_-23). ^13^C NMR (125 MHz, CDCl_3_ *-d*) δ_C_ 197.1 (C-1), 163.3 (C-12), 159.0 (C-8), 155.6 (C-3), 145.2 (C-6), 137.6 (C-10), 136.4 (C- 5), 133.3 (C-16), 124.0 (C-11), 121.7 (C-15), 120.2 (C- 4), 120.5 (C-7), 119.6 (C- 2), 112.6 (C- 13), 108.9 (C- 9), 74.0 (C-25), 71.1(C-21), 65.1 (C-19), 49.3 (C-20), 29.1 (C-22), 28.3 (C-23), 27.7 (C-14), 25.7 (C-17), 17.7 (C-18), 16.3 (C-24) (Supplementary Figures S1, S7). All cited values are in accordance with previously published data by Ballantine et al., 1973.

### Ergosterol (2)

White amorphous powder exhibits blue color after spraying the chromatogram with *p*-anisaldehyde/sulfuric acid reagent. Positive mode LR-ESI-MS *m/z*: 379.3 [M + H- H_2_O] ^+^. ^1^H NMR (400 MHz, CDCl_3_ *-d*) δ_H_ 5.58 (1H, d, *J* = 3.2 Hz, H-6), 5.40 (1H, d, *J* = 2.0 Hz, H-7), 5.28–5.16 (2H, m, H-22, H-23), 3.69–3.63 (1H, m, H-3), 2.49 (1H, d, *J* = 16.7 Hz, H-4_a_), 2.30 (1H, t, *J* = 13.2 Hz, H-4_b_), 2.09–1.85 (5H, m, CH_2_-2, H-9, H-16_a_, H-17), 1.28 (2H, s, CH_2_-12), 1.07 (3H, d, *J* = 6.8 Hz, CH_3_-21), 0.96 (3H, s, CH_3_-19), 0.94 (3H, d, *J* = 6.8 Hz, CH_3_-28), 0.89 (3H, d, *J* = 6.0 Hz, CH_3_-27), 0.85 (3H, d, *J* = 6.4 Hz, CH_3_-26), 0.65 (3H, s, CH_3_-18). ^13^C NMR (CDCl_3_-*d*, 100 MHz), δ_C_ 141.4 (C-8), 139.8 (C-5), 135.6 (C-23), 132.0 (C-22), 119.6 (C-6), 116.3 (C-7), 70.5 (C-3), 55.8 (C-17), 54.6 (C-14), 46.3 (C-9), 42.8 (C-13, 24), 40.8 (C-4), 40.4 (C-20), 39.1 (C-12), 38.4 (C-1), 37.0 (C-10), 33.1 (C-25), 32.0 (C-2), 29.7 (C-16), 23.0 (C-15), 22.7 (C-11), 21.1 (C-21), 20.0 (C-27), 19.7 (C-26), 17.6 (C-19), 16.3 (C-28), 12.0 (C-18) (Supplementary Figures S8, S10). Data was compared with previously published values stated by Shirane et al. (1996).

### Iso-Emericellin (3)

It was isolated as yellow amorphous powder. Shown pink color after spraying with *p-*anisaldehyde/sulfuric acid and heating the chromatogram. UV (MeOH, λ_max_, nm): 266 and 296 nm. Positive mode LR-ESI/MS: *m/z*: 431.2 [M + Na] ^+^. ^1^H NMR (500 MHz, CDCl_3_ *-d*) δ_H_ 12.87 (1H, s, OH-4), 7.41 (1H, d, *J* = 8.5, H-2), 7.22 (1H, s, H-12), 6.69 (1H, d, *J* = 8.5, H-1), 5.55 (1H, m, H-17), 5.01 (2H, m, CH_2_-14), 5.27 (1H, m, H-22), 4.38 (2H, d, *J* = 7.5, CH_2_-16), 3.33 (2H, d, 7.0, H-21), 2.39 (3H, s, H-15), 1.75 (3H, s, CH_3_-20), 1.70 (3H, s, CH_3_-25), 1.68 (3H, s, CH_3_-24), 1.65 (3H, s, CH_3_-19). ^13^C NMR (125 MHz, CDCl_3_ *-d*) δ_C_ 184.7 (C-7) 159.1 (C-4), 154.2 (C- 13), 153.7 (C-6), 152.5 (C-10), 142.6 (C-11), 139.3 (C-18), 136.9 (C-2), 134.1 (C-9), 133.4 (C-23), 122.9 (C-3), 121.8 (C-22), 119.6 (C-17), 119.4 (C-12), 118.1 (C-8), 108.3 (C-5), 105.7 (C-1), 72.2, (C-16), 57.2 (C-14), 27.1 (C-21), 25.9 (C-20), 25.8 (C-25), 18.1 (C-19), 17.8 (C-24), 17.7 (C-15) (Supplementary Figures S11, S18). Complete identification was based on the aforementioned analysis as well as comparison with previously published data for iso-emercellin (Bringmann et al., 2003).

### Sterigmatocystin (4) and Dihydrosterigmatocystin (5)

They were purified as pale-yellow amorphous powder as well as they display a yellow color rapidly changed to brown upon spraying with *p*-anisaldehyde/sulfuric acid and heating the chromatogram. Positive mode LR-ESI/MS *m/z*: 347.1 [M + Na] ^+^, 671.1 [2M + Na] ^+^, and 349.1 [M + Na] ^+^, 675.1 [2M + Na] ^+^ for compounds 4 and 5, respectively. ^1^H and ^13^C NMR data are represented in Table 3 (Supplementary Figures S19, S31). All values of both compounds were compared to previously cited data for sterigmatocystin and dihydrosterigmatocystin by Davies et al., 1960, Gorst-Allman et al., 1977; Zhu and Lin, 2007.

**TABLE 3:** ^1^H NMR (500 MHz), ^13^C NMR (125 MHz) data in CDCl_3_-*d* and 2D-NMR (^1^H-^1^H COSY and ^13^C-^1^H HMBC) key correlations for pure compounds 4 and 5.

Position	4				5			
	δ_C_	δ_H_	COSY	HMBC	δ_C_	δ_H_	COSY	HMBC
1	181.4	—	—	—	181.4	—	—	—
2	109.1	—	—	—	109.0	—	—	—
3	162.5	—	—	—	162.4	—	—	—
4	111.4	6.70 (dd, 8.5, 1.0)	5	C-2,3,7,6	111.3	6.69 (dd, 1.0, 8.5)	5	2, 6
5	135.6	7.41 (t-like, 8.5)	4, 6	C-2,3,7	135.6	7.43 (t like, 8.5)	4, 6	3, 7
6	105.8	6.80 (d, 7.5 over lapped)	5	C-1,2,7	105.9	6.76 (dd, 8.5, 1.0)	5	2,4,7
7	154.8	—	—	—	155.0	—	—	—
8	154.2	—	—	—	154.5	—	—	—
9	106.5	6.34 (d, 8.5)	10	13, 11, 8, 1	105.8	—	—	—
10	164.7	7.21 (d, 8.5)	9	12, 8, 14	166.2	—	—	—
11	90.4	6.35 (s)	—	1, 8, 9, 13, 10, 12	89.8	6.30 (s)	—	9, 10, 12, 13
12	163.6	—	—	—	163.5	—	—	—
13	106.2	—	—	—	105.4	—	—	—
14	113.4	6.76 (d, 7.5)	15	9, 10, 15, 16, 17	113.5	6.44 (d, 5.5)	15	10, 11, 15, 16, 17
15	48.3	4.72 (dt, 7.5, 2.5)	14, 16	9, 10, 14,16,17	44.3	4.16 (dd, 5.5, 9.5)	14, 16	14
16	102.5	5.40 (t-like, 2.5)	15, 17	14, 15, 17	31.4	2.27 (m)	15,17_a_, 17_b_	9, 14,15, 17
17_a_	145.3	6.42 (dd, 2.5, 2.0)	16	14, 15, 16	67.9	4.11 (m)	16, 17_b_ 16,17_a_	15
17_b_						3.63 (td, 9.0, 6.5)		
18	56.7	3.91 (s)	—	11, 12	56.8	3.93 (s)	—	12
OH	—	13.18 (s)	—	2, 3, 4, 5	—	13.20 (s)	—	2, 3, 4

Data between parentheses represent (multiplicity and *J* value in Hz).

### Versicolorin B (6)

It was obtained as an orange amorphous powder displaying orange fluorescence under long-UV light (λ_365_ nm). UV (MeOH, λ_max_, nm): 291.6, 359.4, 452. Negative mode LR-ESI/Ms *m/z*: 338.9 [M-H]^-^. ^1^H NMR (600 MHz, CDCl_3_-*d*) δ_H_ 7.18 (1H, d, *J* = 2.4, H-5), 7.17 (1H, s, H-4). 6.54 (1H, d, *J* = 2.4, H-7), 6.48 (1H, d, *J* = 6.0, H-11), 4.14 (1H, m, H-12), 4.13, 3.60 (2H, m, CH_2_-14), 2.31, 2.27 (2H, m, CH_2_-13). ^13^C NMR (150 MHz, CDCl3*-d*), δ_C_ 192.9 (C-9), 181.8 (C-10), 166.1 (C-6), 165.5 (C-8), 165.4 (C-3), 161.4 (C-1), 132.8 (C-10_a_), 132.0 (C-4_a_), 120.0 (C-2), 113.3 (C-11), 110.9 (C-9_a_), 109.2 (C-5), 108.0 (C-8_a_), 107.8 (C-7), 101.2 (C-4), 43.9 (C-12), 67.1 (C-14), 30.2 (C-13) (Supplementary Figures S32, S38). Metabolite **6** was confirmed as versicolorin B by comparing the elucidated values with previously reported data in the literature (Cole and Cox, 1981; Hawas et al., 2012; Jakšić et al., 2012).

### Diorcinol (7)

It was isolated as pale-yellow oil that gave intense pink color on spraying with *p-*anisaldehyde/sulfuric acid reagent and heating the chromatogram. UV (MeOH, λ_max_, nm): 208, 278. Positive mode HR-ESI/MS: *m/z*: 231.1102 [M + H] ^+^ and negative mode HR-ESI/MS: *m/z*: 229.1232 [M- H]^−^. ^1^H NMR (500 MHz, CDCl_3_-*d*) δ_H_ 6.34 (4H, d, *J* = 6.5 Hz, CH-4/4′, CH-6/6′); 6.23 (2H, t-like, *J* = 2.0 Hz, CH-2/2′); 4.68 (2H, s, CH-3/3′ OH); 2.20 (6H, CH_3_-7/7′). ^13^C NMR (125 MHz, CDCl_3_ *-d*), δ_C_ 158.1 (C-1/1′), 156.5 (C-3/3′), 141.0 (C-5/5′), 112.1 (C-6/6′), 111.1 (C-4/4′), 103.5 (C-2/2′), 21.5 (C-7/7′) (Supplementary Figures S39, S46). Hence, 3 was identified as diorcinol by comparing the data with those previously reported in the literature (Itabashi et al., 1993).

**FIGURE 3:**
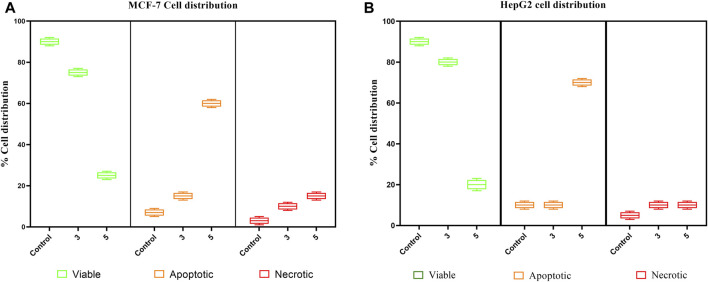
Box plot representing mode of cell death of compounds 3 and 5 on **(A)** the MCF-7 breast cancer cells, **(B)** HepG2 hepatocellular carcinoma cells.

The pure, identified secondary metabolites (**1**, **3–7**) were screened for their cytotoxicity on the MCF-7 and Hep G2 cancer cells in MTT cytotoxicity assay. The results were represented in terms of IC_50_ (µM±SD) and displayed in Table 1. Generally, the HepG2 cell line was sensitive to most of the applied treatments than the MCF-7 cell line. Amongst, metabolite **5** displayed modest cytotoxicity on HepG2 with IC_50_ = 161.81 ± 5.2 µM, followed by **3** (IC_50_ = 232.07 ± 4.9 µM) and **4** (IC_50_ = 265.20 ± 9.7 µM), while compounds **1**, **6**, and **7** exhibited week cytotoxicity with IC_50_ > 400 µM. On the other hand, the cytotoxicity on the MCF-7 cells revealed that **3** exhibited the best activity with IC_50_ = 225.21 ± 7.4 µM, while all other metabolites displayed week effect with IC_50_ approximately above 350 µM. Accordingly, the mode of cell death of the most active metabolites, **3** and **5,** represented as necrosis and apoptosis modes were quantified using EB/AO staining method. The results declared that both compounds showed shallow necrotic effect on the MCF-7 cells represented by 10 and 15% (Figure 3A), respectively while 5 exerted an overt apoptotic mode which is quantified as 60% in comparison to **3** (15%). On the other hand, the cytotoxicity mode displayed by 5 on the Hep-G2 cell line was measured as 70% apoptotic and 10% necrotic (Figure 3B). To unravel the molecular target involved in the cytotoxicity of the bioactive hits, molecular docking study was conducted for metabolites **1**, **3–7** on Hsp90 (PDB code: 4YKQ) as a proposed target commonly involved in cancer prognosis. The docking scores of the ligand and all tested metabolites along with the amino acid residues involved in their interaction with the molecular target were displayed in Table 5. Despite the ATP-binding pocket of Hsp-90 includes various hydrophobic, polar, and charged amino acids, Ala-55, Met-98, and Thr-184 were critically incorporated in its inhibitory activity (Rampogu, et al., 2019, Figure 4). Concerning the docking of the most active metabolites, the careful inspection of **3** docked within the binding cleft showed that it displayed various hydrophobic interactions with the critical amino acids allocated in the catalytic cleft with a net resulting binding score of −7.7 Kcal/mol (Table 5). The chromone core showed hydrophobic interaction with Asp-54 meanwhile, ring-B and methyl group at position 15 displayed the same type of interaction with Ala-55. Also, the methyl functionality at position 20 displayed hydrophobic interactions with amino acids Leu-107, Met-98, and Phe-138. Compound **4** and its dehydro-analog (**5**) manifested almost similar interactions represented by the *pi*-stacking with the alkyl residue of Met-98, while a *pi*-stacking interaction with the amide functionality of Asp-54. In all, resulted in excellent near binding scores of −8.4 and −8.3 Kcal/mol, respectively. The docking results of the remaining, less active metabolites were shown in Table 5 and detailed discussion available as supplementary data .

**FIGURE 4:**
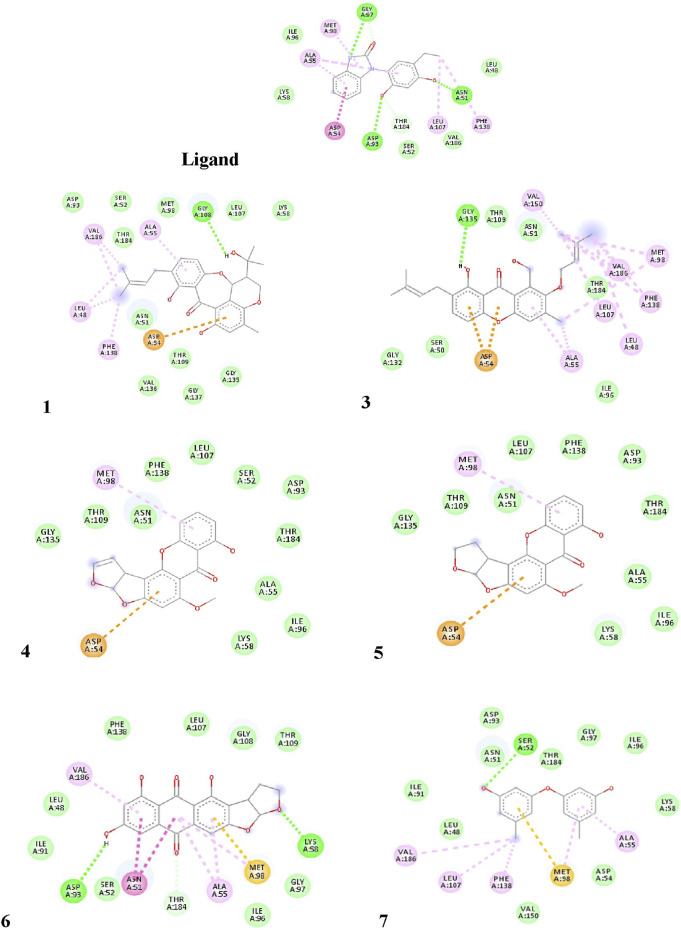
Proposed binding mode of the ligand (CS301) and isolated compounds 1-7 in the 2D representation of the X-ray crystal structure of Hsp-90 (PDB: 4YKQ) showing various interactions with the critical amino acids within the catalytic kinase domain.

**FIGURE 5:**
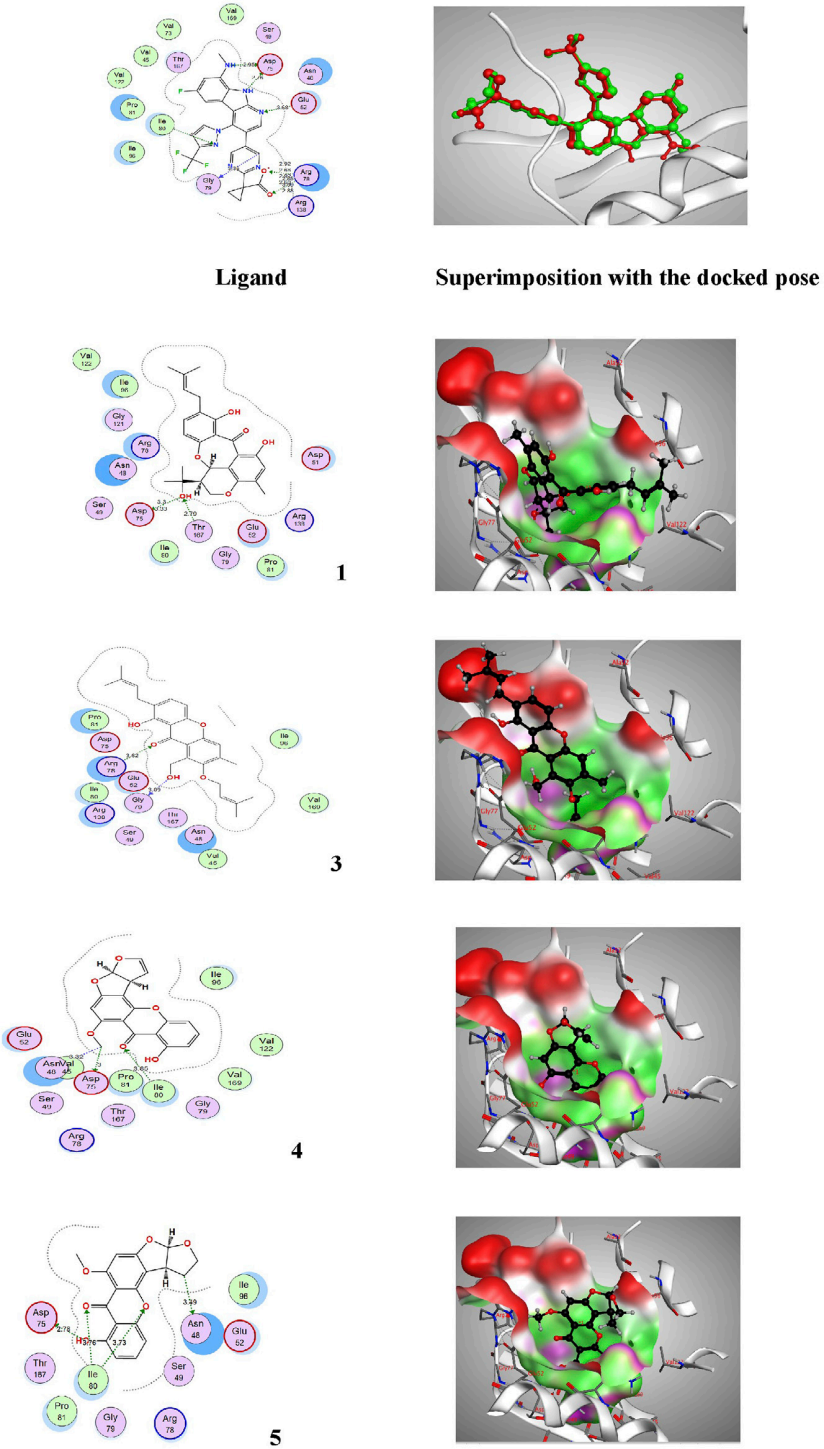
Proposed binding mode of the ligand (EZ6) and isolated compounds 1, 3–5 in 2D and 3D representation of the X-ray crystal structure of DNA gyrase (PDB: 6M1J) showing various interactions with the critical amino acids within the vicinity of the active site. Colour codes: (Bright red (Asp, Glu), yellow (Cys, Met), blue (Lys, Arg), orange (Ser, Thr), mid blue (Phe, Tyr), Cyan (Asn, Gln), Light grey (Gly), green (Leu, Val, Ile), dark grey (Ala), pink (Trp), pale blue (His), flesh (Pro).

On the other side, the results of the antimicrobial susceptibility assay using the agar well diffusion method showed that all tested compounds (**1, 3–5**) possess antibacterial activity that varied among the standard strains, with almost no apparent antifungal potential (Table 2). Interestingly, Gram-negative strains, especially *P. aeruginosa*, were more sensitive to the tested treatments as compared to the Gram-positive ones (Table 2). For instance, compound **1** inhibited the growth of *S. aureus, S. faecalis*, and *P. aeruginosa* with inhibitory zones measured as 16, 13, and 14 mm, respectively which is almost higher than the standard antibiotic, chloramphenicol (9–13 mm), meanwhile *E. coli*, *L. monocytogenes*, and *K. pneumoniae* being resistant to **1**’s treatment. On the other hand, the inhibition zones exerted by **3** and **4** (9–15 mm) on *S. aureus, S. faecalis*, and *P. aeruginosa* were almost comparable to that of the standard antibiotic gentamycin (10 mm) but higher than that of chloramphenicol (9–13 mm, Table 2). Meanwhile, both compounds displayed completely far MIC in the broth micro dilution method being 1,000 and 125 µg/ml, respectively (Table 4 **)**. Else way, compound **5** was the most active among the tested samples on *P. aeruginosa* (18 mm) which is almost double the measured inhibitory zone for chloramphenicol (9 mm). A keen look at the results of MIC (Table 4) showed that **4** and **5** displayed the best and almost equivalent MIC on *P. aeruginosa* (MIC = 125 µg/ml) while, the rest of the tested compounds displayed MIC values ≥ 1,000 µg/ml. Ironically to better correlate the observed antibacterial activity with a validated molecular target, *in silico* docking study was conducted for the bioactive hits on the DNA gyrase enzyme (PDB code: 6M1J) as a proposed molecular target using MOE, 2019.0102 platform. The virtual setup was first validated by self-docking of the co-crystallized ligand, **EZ6**, in the vicinity of the binding site of the enzyme, with net docking score = −15.5981 kcal/mol and RMSD = 0.7977 Å. Examination of the binding interactions of EZ6 to the active site of the enzyme, showed the occurrence of strong hydrogen bond interactions with the amino acids; Glu-52, Asp-75, Arg-78, and Ile-80 (Figure 5). On the other side, all tested compounds (**1, 3, 4,** and **5**) showed a good binding energy score ranging from −10.2796 to −12.3974 kcal/mol (Table 5). For instance, the 2D presentation of the docked pose of **1** (Figure 5) showed hydrogen bond interaction between hydroxyl group at position 27 and amino acids Asp-75 and Thr-167, while **3** displayed two hydrogen bonding interactions. One of them is delineated between the hydroxy methylene group at position 14 and the amino acid Gly-79 while the second is between the pyrane-4-one functionality and amino acid Arg-78. It is worth noting that compounds **4** and **5** displayed comparable docking scores −10.28 deduced from their interaction with Asn-48, Asp-75, and Ile-80 through non-classical and normal hydrogen bonds, respectively (Figure 5; Table 5).

**TABLE 4:** Minimum inhibitory concentrations (MICs in µg/mL) of the EtOAc extract and Pure compounds 1, 3-5 against most sensitive reference Gram positive and Gram negative human pathogens in microbroth dilution assay.

Standard strains	EtOAc	1	3	4	5
*S. aureus*	1,000	1,000	1,000	1,000	—
*S. faecalis*	1,000	1,000	1,000	1,000	—
*K. pneumonia*	>1,000	—	>1,000	—	—
*P. aeruginosa*	625	1,000	1,000	125	125

**TABLE 5:** Docking scores and amino acids residues involved in the interaction of the ligand and isolated metabolites (1, 3–7) with Hsp90 and DNA gyrase enzyme.

Target Compound	Hsp90		DNA gyrase	
	Docking score (kcal/mol)	Interacting amino acids	Docking score (kcal/mol)	Interacting amino acids
Ligand	−10.89	**Ala-55**, Asp93, Asp-54, Asn-51, Gly-97, Leu-107, **Met-98**, Phe-138, and **Thr-184**	−15.59	**Glu-52**, **Asp-75**, **Arg-78**, **and Ile-80**
1	−9.00	Gly108, Leu48, Asp54, **Ala55**, Phe138, Val186	−11.46	**Asp75**, Thr167
3	−7.70	Leu48, Leu107, **Ala55**, Gly135, Phe138, Val186, **Met98**, Asp54, Val 150	−12.39	**Arg78**, Gly79
4	−8.40	Asp54, **Met98**	−10.28	Asn48, **Asp75**, **Ile80**
5	−8.30	Asp54, **Met98**	−10.27	Asn48, **Asp75**, **Ile80**
6	−8.90	Lys58, Asn51, **Ala55**, Val186, **Met98**, **Asp93**, **Thr184**	—	—
7	−7.50	Leu107, **Ala55**, Gly108, Phe138. Val186, **Met98**	—	—

Residues written in **bold** are critical amino acids incorporated in the required activity.

## Discussion

**FIGURE 6:**
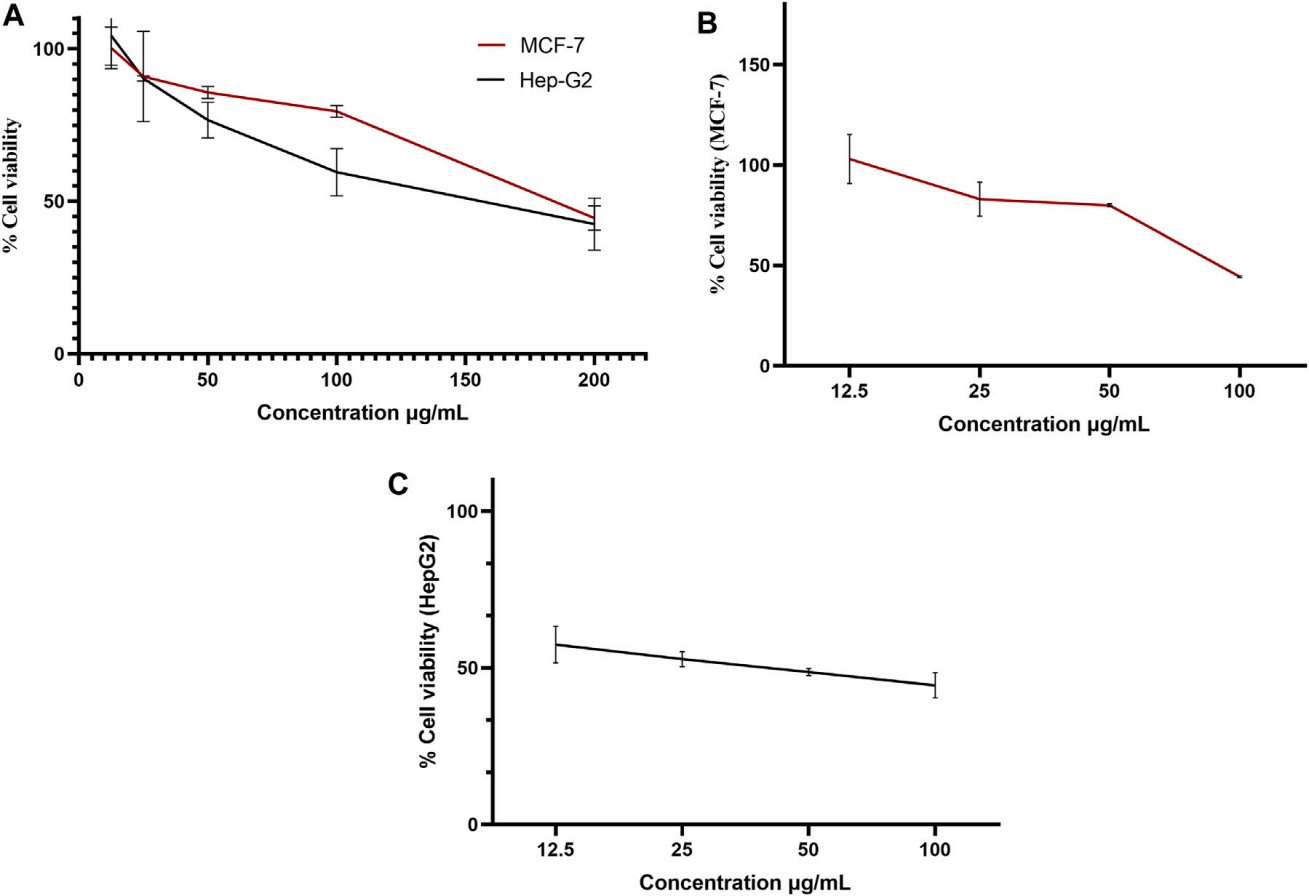
Dose-response curve of the % viability versus concentrations in MTT assay on MCF-7 and HepG2 cell lines for **(A)** ethyl acetate extract, **(B)** iso-emercellin and **(C)** dihydrosterigmatocystin.

Endophytes are gaining biotechnological and industrially relevance as a result of their ability to biosynthesized secondary metabolites that could serve as biocontrol, antimicrobial, anticancer, and anti-oxidants (Gouda et al., 2016; Yadav, 2018). As part of our ongoing research on the discovery of bio-active hits from terrestrial endophytes with potential cytotoxicity and antimicrobial activities, the EtOAc extract of the endophytic fungi, *Aspergillus* sp. isolated from sterilized *T. stans* leaves was screened in the MTT assay and agar well diffusion assays, respectively. Concerning the anticancer screening, it was conducted on the MCF-7 breast adenocarcinoma and HepG2 hepatocellular carcinoma cell lines. Both cell lines were nominated based on the reported literature as being among cancer’s prevalent types due to their high global mortality rates (Dasgupta, et al., 2020; Bray et al., 2018). The tested extract showed promising dose response activity (Figure 6) which motivated us for the fractionation and isolation of its major metabolites to discover new bio-active and targeted hits. The chromatographic separation afforded seven known metabolites belongs to various scaffolds and categorized as four xanthones (**1, 3, 4,** and **5**), one sterol (**2**), one-anthraquinone (**6**), and one-simple phenolic (**7**). Eventually, all metabolites were subjected to cytotoxicity screening on the same cell lines as per stated for the extract. The screening results showed that compounds **3** and **5** displayed the best cytotoxicity results. Xanthones are a unique class of secondary metabolites with broad array of bioactivities, just as anti-microbial, anti-carcinogenic, and anti-oxidant. Hence, they are designated as “privileged structures” (Negi et al., 2013). Prior literature (Obolskiy et al., 2010; Shan et al., 2011) has figured out that the anti-cancer activity is utmost in linear xanthones containing at least tetra-oxygen functionality and anchored with two isoprenyl units, a chemical feature which is typically fulfilled by metabolite **3**. Therefore, the cytotoxic activity exerted by **3** on both cell lines may be correlated to the forementioned structure-activity relationship. On the other hand, **4** is a renowned, potent mycotoxin that is classified by the international agency of research in cancer as group 2B carcinogens (International Agency for Research on Cancer (IARC, 1976; International Agency for Research on Cancer (IARC, 1987). However, several *in vitro* studies stated that **4** is more cytotoxic than aflatoxin with mutagenic effect on human adenocarcinoma lung cells A549 and human esophageal epithelial cells Het-1A (Wang et al., 2013). Moreover, Gao and co-workers report its induction for oxidative DNA damage in a human liver-derived cell line (Gao et al., 2015), while Lee, *et al.* accounts its significant cytotoxicity against A-549, ovarian (SK-OV-3), melanoma (SK-MEL-2), CNS (XF-498), hepatocellular (HepG2), and colon (HCT-15) adenocarcinoma cell lines (Lee et al., 2010). Ultimately, the toxic potential of the Δ^16^ sterigmatocystin (derivative **5)** has been poorly investigated so far hence, this study is almost the first to report its preliminary *in vitro* cytotoxicity. Our results demonstrated that cytotoxicity of **5** on HepG2 is approximately two-fold better than its parent **4**, while displayed far less activity on MCF-7. This selectivity may be explained by the previous data which stated that sterigmatocystin derivatives are activated by the hepatocellular cytochrome P450 enzymes producing reactive epoxide species (Cabaret et al., 2010). Additionally, the acquired saturation of Δ^16^ double bond may promote the partition of **5** across biological membranes. Hence, both mechanisms may be in action for **5** activities. Incidentally, analyzing the mode of cell death has been an integral requirement of many biological studies, just as cytotoxicity. Cancerous cell death may lie in one of the four main categories that have been previously reported in the biomedical literature (Liu et al., 2018). Apoptosis and senescent death (SD) which considered as physiological modes, while necrosis and stress-induced cell death (SICD) which are known as pathological modes (Liu et al., 2018). Accordingly, the necrosis and apoptosis modes of the most active compounds **3** and **5** were selected and quantified using EB/AO staining assay. The results revealed that **5** displayed almost cytotoxicity via apoptotic mode while **3** by weak necrotic and apoptotic mode. Apoptosis is a programmed cell death induced by several factors which interestingly, are the same that induce the expression and accumulation of Hsp90 (Avendaño and Menendez, 2015). Hsp90 is an evolutionary conserved molecular chaperone that is crucial in the maintenance and folding of an array of proteins called clients. Some of the Hsp90 clients are oncogenic proteins and are involved in cancer cell hallmarks as cell cycle, cell survival, angiogenesis, metastasis, and apoptosis (Avendaño and Menendez, 2015; Mori et al., 2015). Hsp90 flexible monomer consists of three domains: The N-terminal domain, the middle domain, and C-terminal. The N-terminal owns a distinctive ATP binding site, the middle domain possesses a binding site for the client’s proteins and co-chaperones, while the C-terminal is involved in the Hsp90 dimerization. Interestingly, Rampogu et al., 2019 reported the critical involvement of a panel of amino acids residues just as Ala55, Lys58, Asp93, Ile96, Met98, and Thr184 directed at inhibiting the ATP-binding activity of Hsp90 followed by the subsequent inhibition of oncogenic downstream signaling. So, a molecular docking study was conducted to examine the binding interactions of the isolated compounds compared to the validated binding ligand in the ATP binding cleft. The results revealed that **5** shows strong affinity to the critically assignable amino acids of the ligand and those previously stated in the literature just as Leu48 and Met98 with favorable fitting to the binding site in an approximately U-shaped conformation. In all, inhibits Hsp90 catalytic activity which is not only results in rapid degradation of its client proteins but also induces apoptosis in cancerous cells (Avendaño and Menendez, 2015; Mori et al., 2015). On the other hand, although compound **3** possesses multiple hydrophobic interactions with the critical amino acids Ala-55, and Met-98, in addition to other residues (Leu48, Leu-107, Phe-138, Gly135, Val186, Val 150, and Asp54) it displayed a low scoring function than **5** (binding score -7.7 Kcal/Mol) and missed the features required for occupying the Hsp90 binding vicinity. Hence, the results explain that **3** exerted its cytotoxic mode may be at least in part through a combination with another molecular target.

Antibiotic resistance, both in the community setting and in hospitals, represents an alarming threat to the burden of infectious diseases (Tajkarimi et al., 2010). The current results showed a promising activity on most of the tested bacteria deduced from the measured zone of inhibition which almost exceeds the tested antibiotics. The obvious activity of the EtOAc extract in addition to metabolites **4** and **5** on *P. aeruginosa* was so interesting. *P. aeruginosa* is a multidrug-resistant pathogen recognized for its ubiquity, profound antibiotic resistance, in addition to its association with serious hospital-acquired infections (Diggle and Whiteley, 2020). *P. aeruginosa* possesses a plethora of resistance mechanisms just as restricted membrane permeability to many antibiotics, the inherited efflux pumps, antibiotic-inactivating enzymes, in addition to the quiet ability for biofilm generation (Diggle and Whiteley, 2020). The current study is the first to report the antimicrobial potential of the bioactive hits, 4 and 5. Both metabolites are related sterigmatocystins with xanthone nucleus attached to a bisfuran moiety, a unique scaffold with previous reports of antimicrobial activity (Negi et al., 2013). It is well known that a wide range of potent natural antimicrobials exerts their action through targeting the DNA gyrase enzyme. DNA gyrase is a ubiquitous member of topoisomerases type II that plays a critical role in bacterial cell survival (Khan et al., 2018). The enzyme temporarily cleaves the DNA strands, hence it is responsible for controlling the topological state, transcription, and replication of DNA (Khan et al., 2018). However, inhibiting this enzyme makes it a desirable and viable therapeutic target for the development of new antibacterial agents. The enzyme possess two subunits, namely A and B. The A subunit is engaged in interactions with DNA, and it contains the tyrosine active site responsible for DNA cleavage, while the B subunit encompasses the active site of ATPase. Herein, we target the docking of the tested metabolites, within the ATP binding domain of *P. aeruginosa* DNA gyrase B subunit (accession code: 6M1J; Rahimi et al., 2016). Although the results demonstrated that both compounds showed less binding score than their corresponding hits, their 3D presentation showed that they possess strong hydrogen bond interactions with a balanced fitting feature in the active site cleft. A dual hallmark which promote both **4** and **5** to act effectively as ATP competitive inhibitors.

To our knowledge, this is the first report about the anticancer potential of metabolite **5** on HepG2 cancerous cells with a proposed mechanism through targeting the catalytic site of the Hsp90. Also, the *in vitro* antibacterial activity of 4 and 5, especially against *P. aureuginosa,* which may be at least in part through targeting the DNA gyrase catalytic domain.

## Conclusions

Aspergillus species are notable machinery for various secondary metabolites just as xanthones which are renowned for their unique scaffold, valuable biological activities, and applications in drug discovery. Modern biotechnological techniques and microbial fermentation processes enable researchers to better understand and manipulate endophytic resources and make them more beneficial in the management of human ailments. The ethyl acetate extract of the rice culture of Aspergillus sp isolated from *T. stans* (L.) leaves represents a potential source of metabolites with cytotoxic and antimicrobial activities. Xanthone-based metabolites may be, at least in part, responsible for the aforementioned activities, however further pharmacological and toxicological studies are necessary. Dihydrosterigmatocystin is a promising apoptotic inducing agent on HepG2 cancerous cells with virtual affinity to the ATP binding cleft of Hsp90 chapron. On the otherside, iso-emericellin has a broad-spectrum antimicrobial activity including *P. aeruginosa*. DNA gyrase enzyme is a validated molecular target that may explain and correlate the noticeable antimicrobial activity of the bioactive candidates. Overall, the future of the discovery of anticancer and antibacterial agents from endophytic fungi is undoubtedly promising and bright.

## Data Availability

The datasets presented in this study can be found in online repositories. The names of the repository/repositories and accession number(s) can be found in the article/Supplementary Material.
