# Supplementary figures and images for: Cytotoxicity, Antimicrobial, and In Silico Studies of Secondary Metabolites From Aspergillus sp. Isolated From Tecoma stans (L.) Juss. Ex Kunth Leaves
Source: Front Chem. 2021 Oct 13;9:760083. doi: 10.3389/fchem.2021.760083 (PMC8548774; doi:10.3389/fchem.2021.760083)

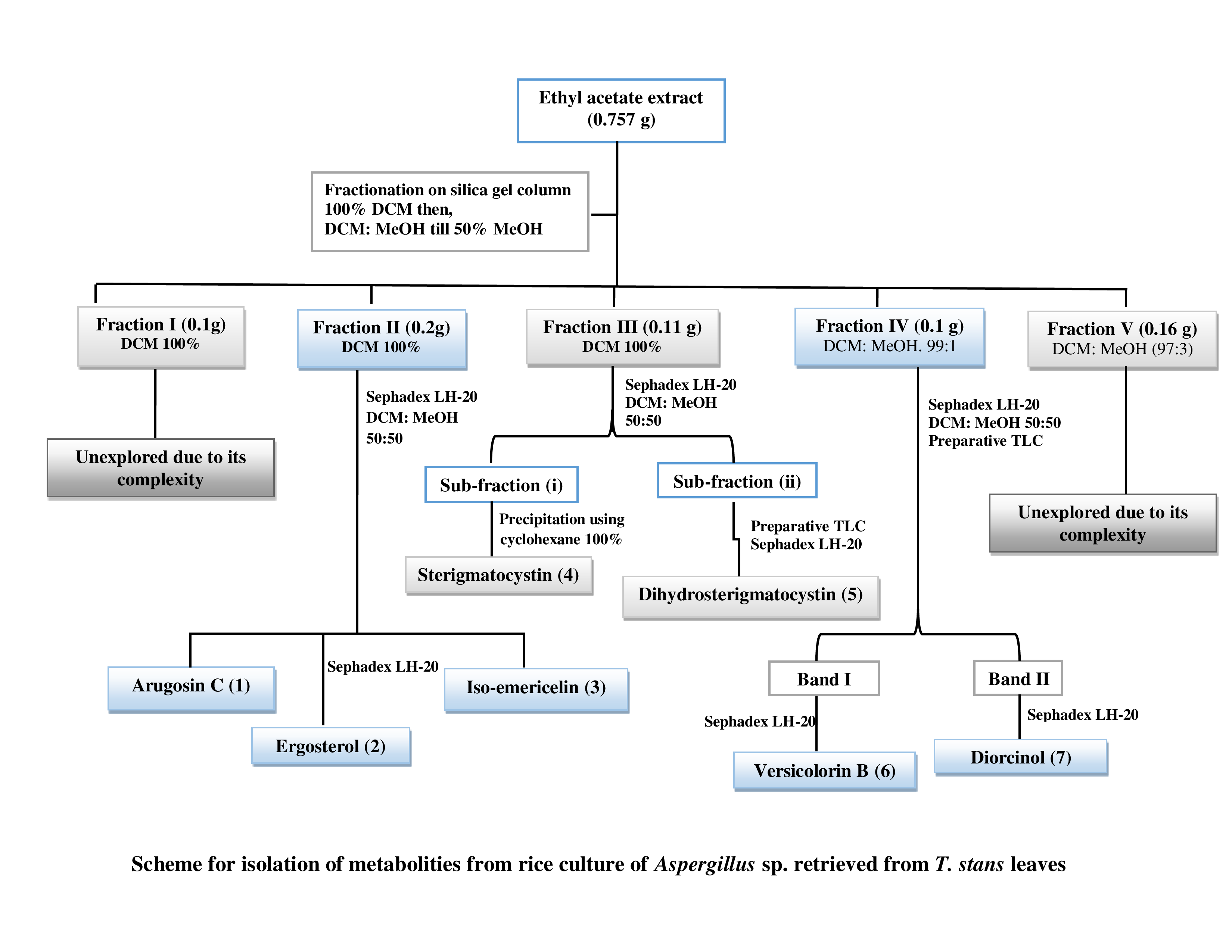

Supplement: Supplementary file 1 [file Image1.TIFF]
