# Supplementary material for: Cytotoxicity, Antimicrobial, and In Silico Studies of Secondary Metabolites From Aspergillus sp. Isolated From Tecoma stans (L.) Juss. Ex Kunth Leaves
Source: Front Chem. 2021 Oct 13;9:760083. doi: 10.3389/fchem.2021.760083 (PMC8548774; doi:10.3389/fchem.2021.760083)
Supplement: Supplementary file 2 [file DataSheet1.PDF]

Supplementary Figures

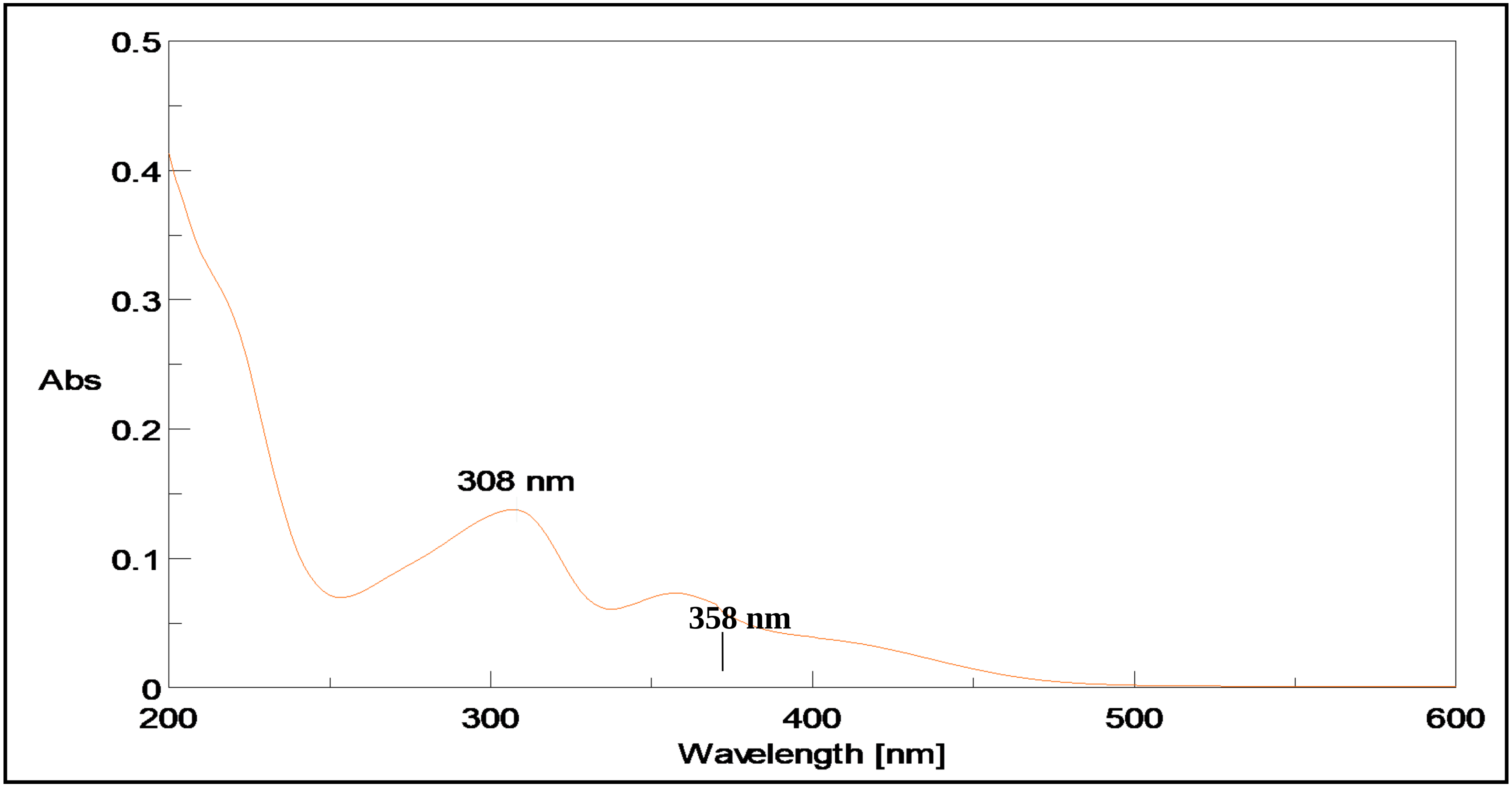

FIGURE S1: UV-spectrum of compound 1 in MeOH

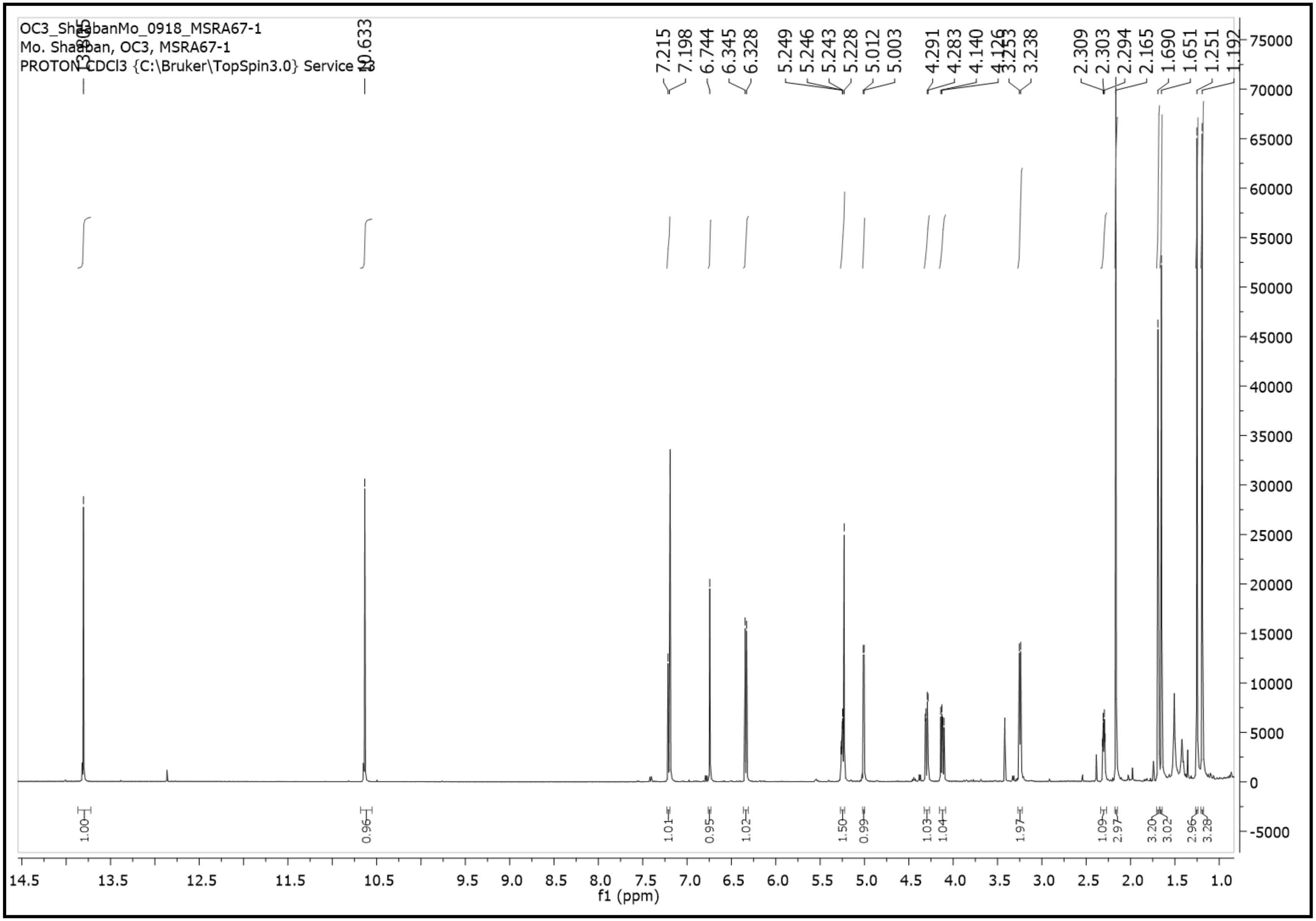

FIGURE S2: ^{1}H NMR spectrum of compound 1 ($CDCl_3$-*d*, 500 MHz)

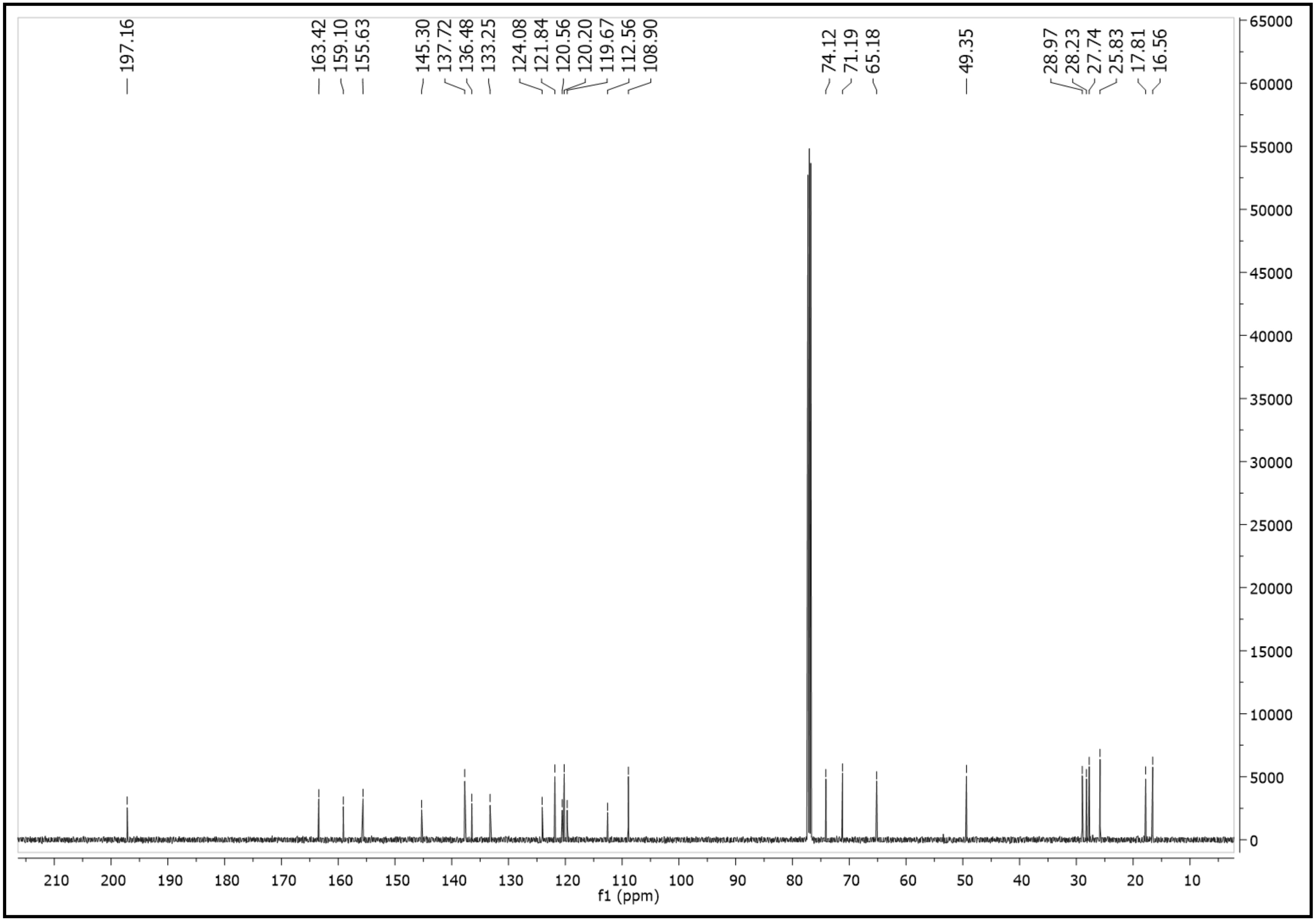

FIGURE S3: ^{13}C NMR spectrum of compound 1 ($CDCl_3$-*d*, 125

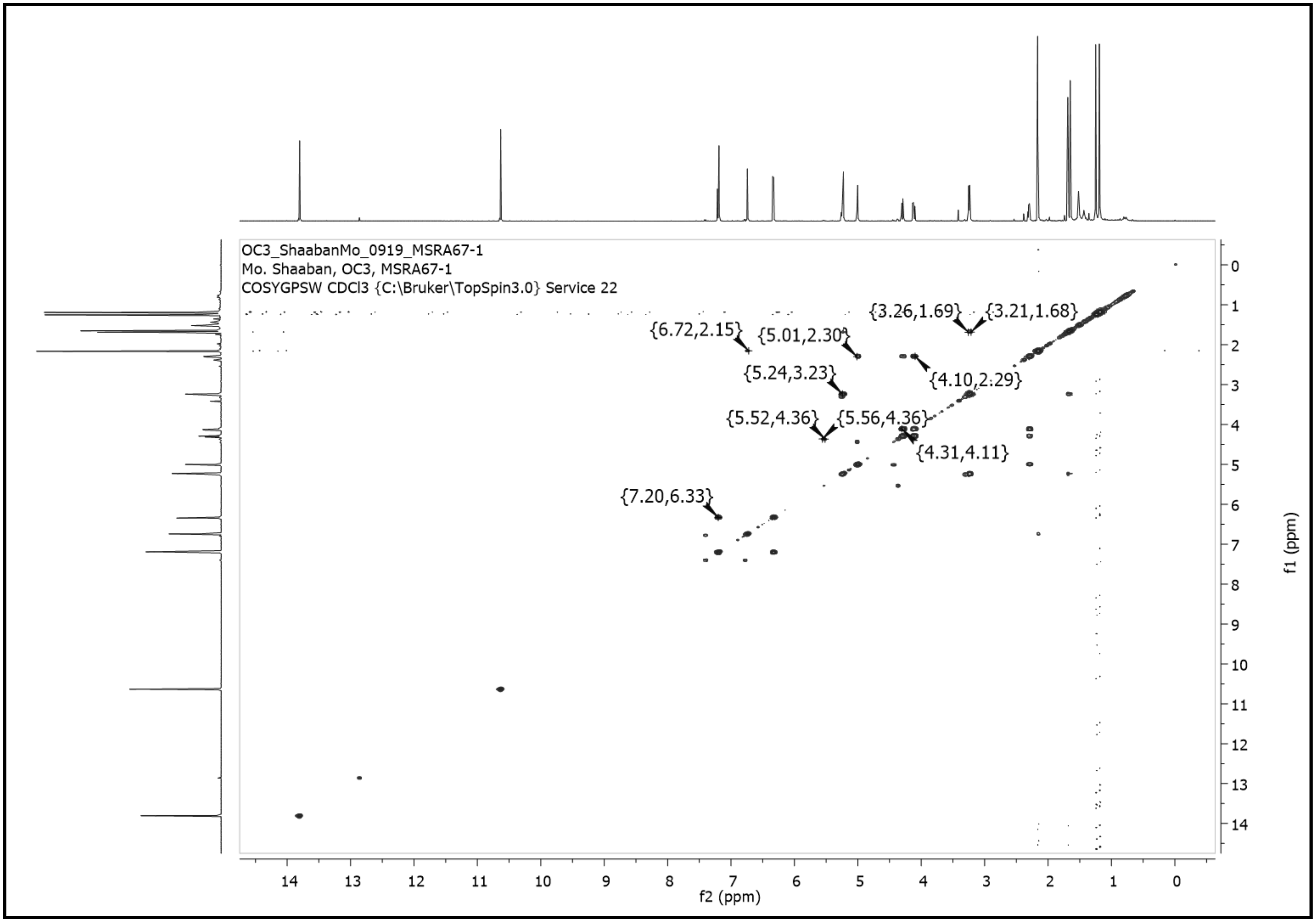

FIGURE S4: ^{1}H-^{1}H COSY spectrum of compound 1 (CDCl$_3$-*d*, 500

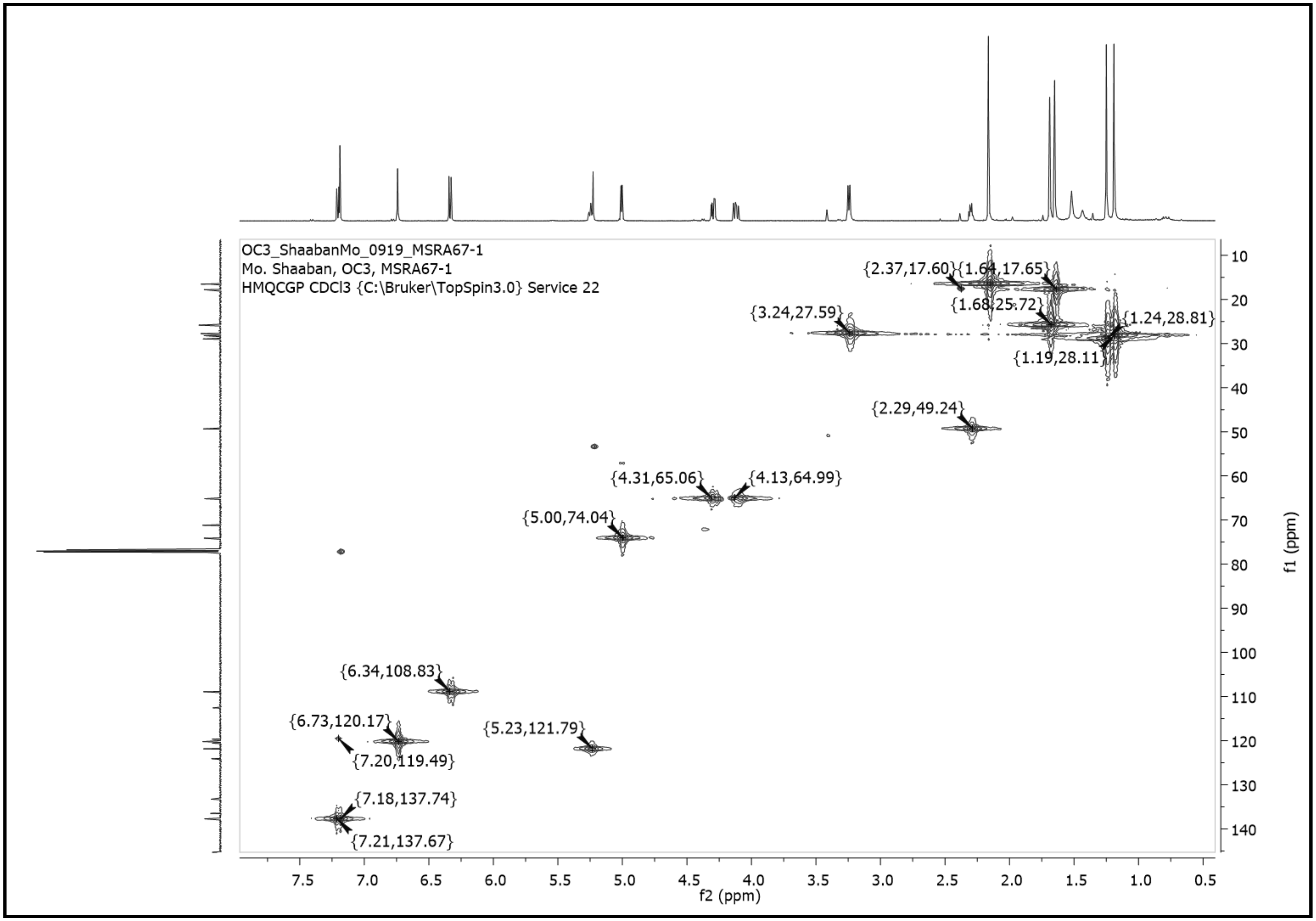

FIGURE S5: HMQC spectrum of compound 1 ($CDCl_3$-*d*, 500 MHz)

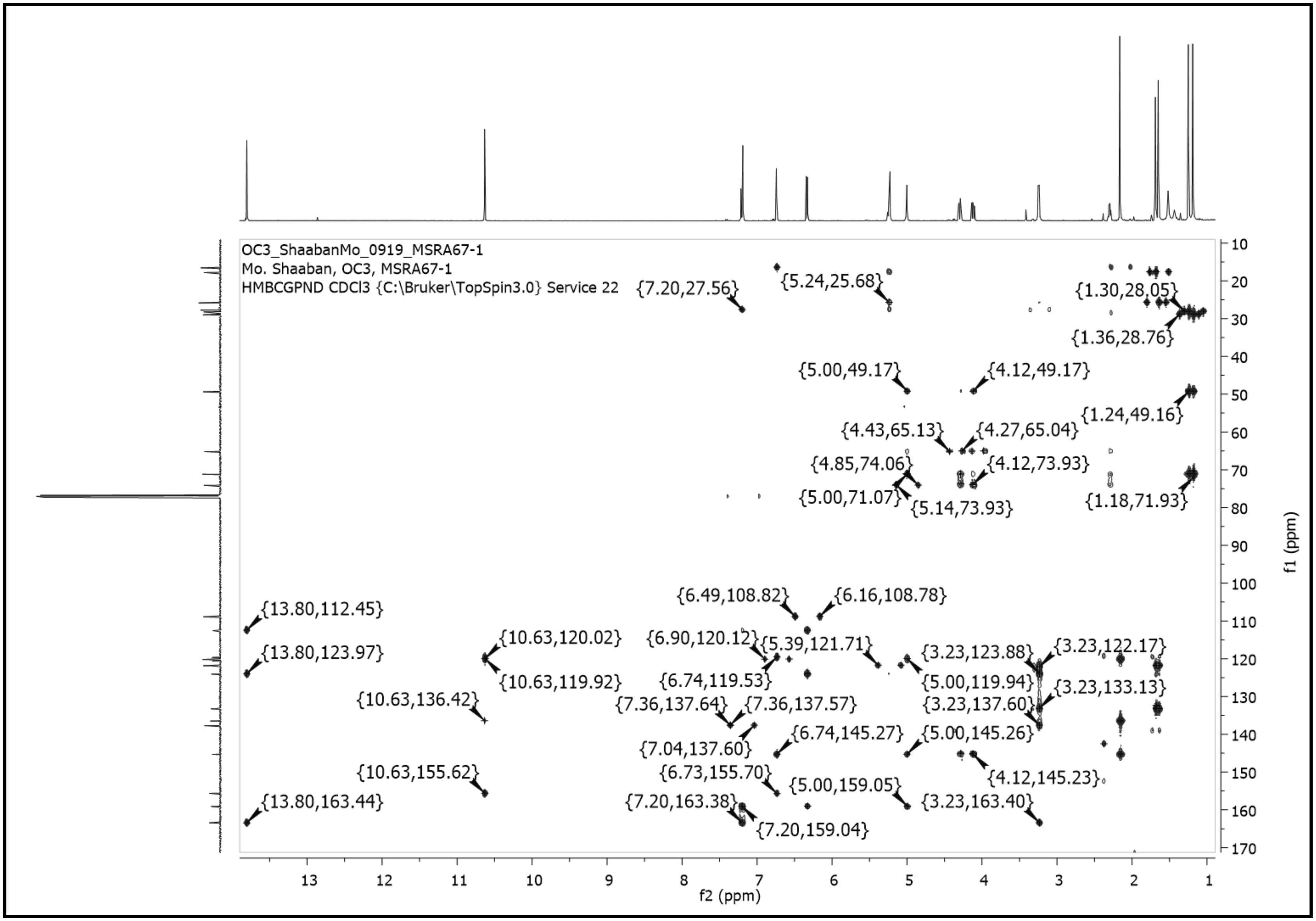

FIGURE S6: HMBC spectrum of compound 1 ($CDCl_3$-*d*, 500 MHz)

Analysis Report

Esquire 3000

Universität Bielefeld

Analysis Info

Analysis Name	OC3_ShaabanMo_0919_MSRA67-1_01.d
Method	Tune-nan.MS
Workgroup	OC3
Operator	S.Heitkamp
Acquisition Date	22.09.2017 15:58:09
Print Date	22.09.2017 16:28:26
Comment	Mo. Shaaban, OC3, MSRA67-1, MeOH

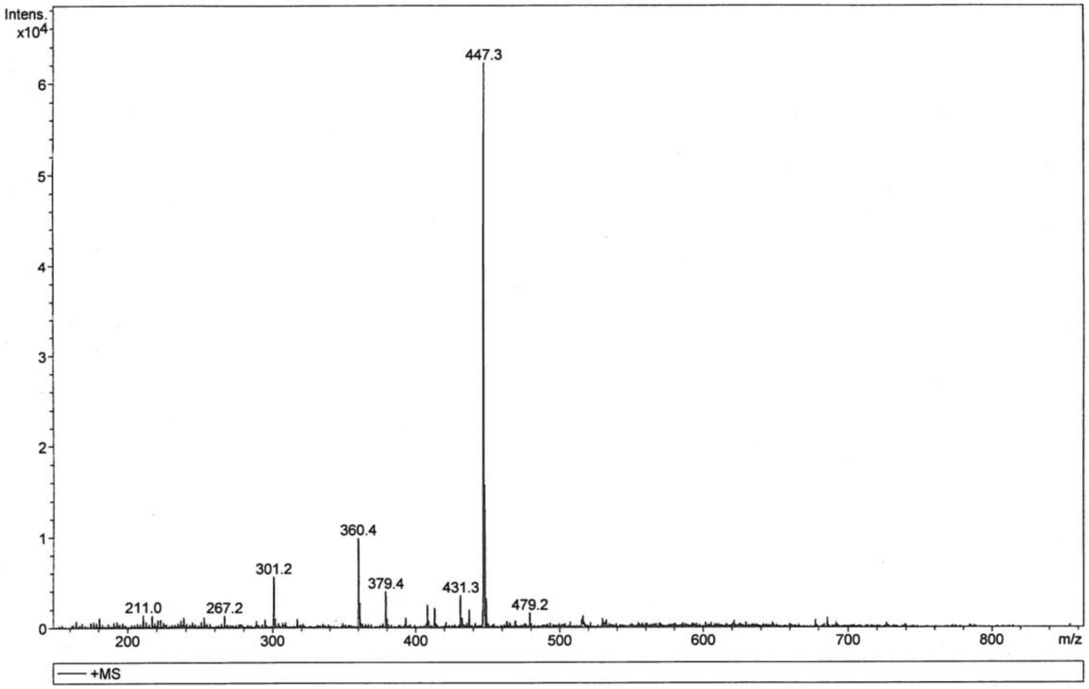

Acquisition Parameter

Mode

Ion Source Type	NanoESI, off-line
Ion Polarity	Positive
Mass Range Mode	Std/Normal
Scan Begin	50 m/z
Scan End	1500 m/z
Averages	20 Spectra
Rolling	on
Rolling, Averages	2 cts

Tune SPS

Target Mass	400 m/z
Compound Stability	40 %
Smart Parameter Setting	active

Tune Instrument

Trap Drive	40.8
Octopole RF Amplitude	120.0 Vpp
Lens 2	-60.0 Volt
Capillary Exit	91.8 Volt
Skim 1	22.9 Volt
Skim 2	6.0 Volt
Lens 1	-5.0 Volt
Cap Exit Offset	68.9 Volt
Octopole	2.54 Volt
Octopole Delta	2.40 Volt
Dry Temp (Set)	100 °C
Nebulizer (Set)	2.00 psi
Dry Gas (Set)	2.00 l/min
Accumulation Time	4683 µs
ICC Target	5000
Charge Control	on

FIGURE S7: Positive ESI-MSspectrum of compound 1

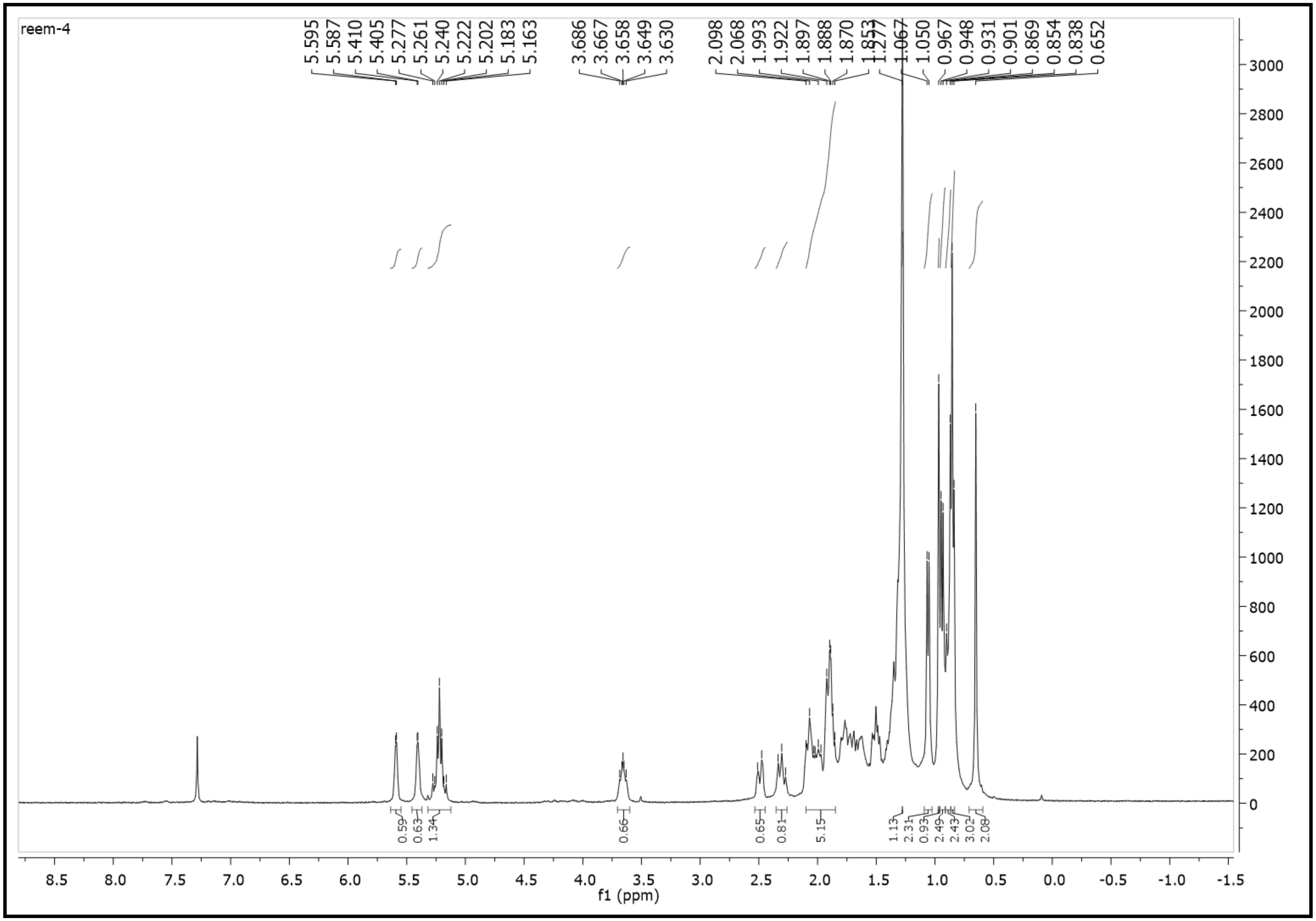

FIGURE S8:^{1}H NMR spectrum of compound 2 ($CDCl_3$-*d*, 400 MHz)

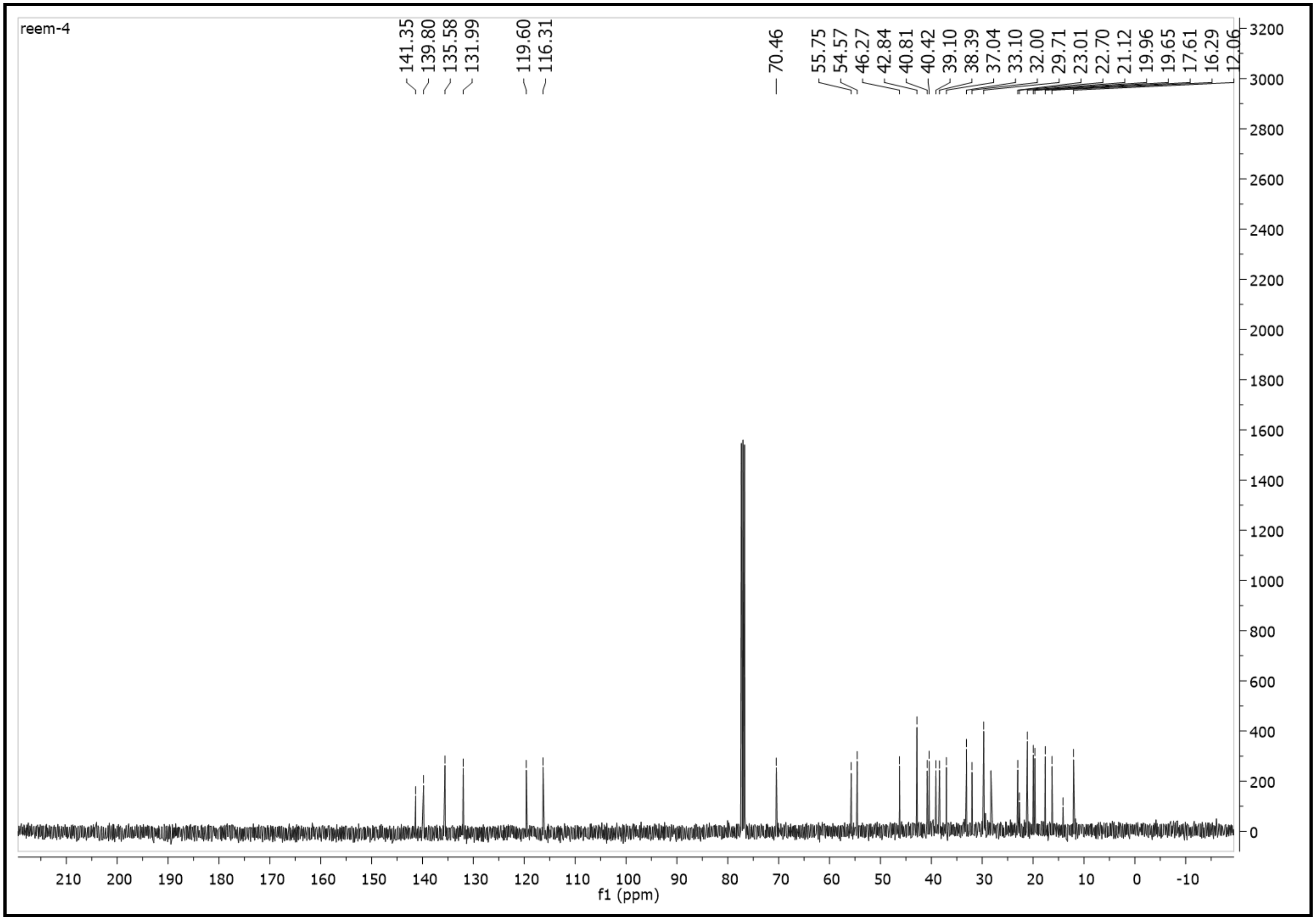

FIGURE S9: ^{13}C NMR spectrum of compound 2 ($CDCl_3$-*d*, 100

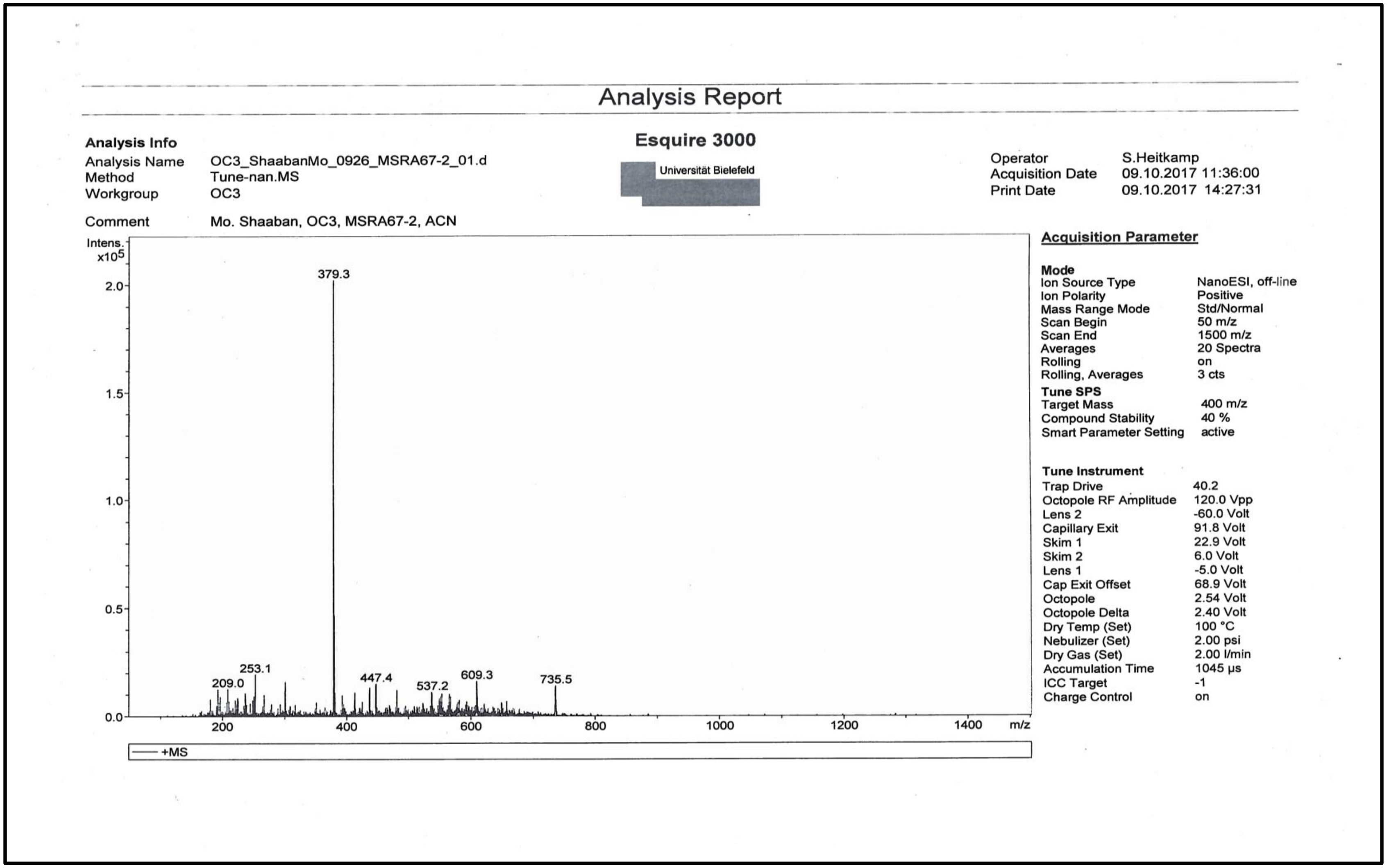

:

FIGURE S10: Positive ESI-MS spectrum of compound 2

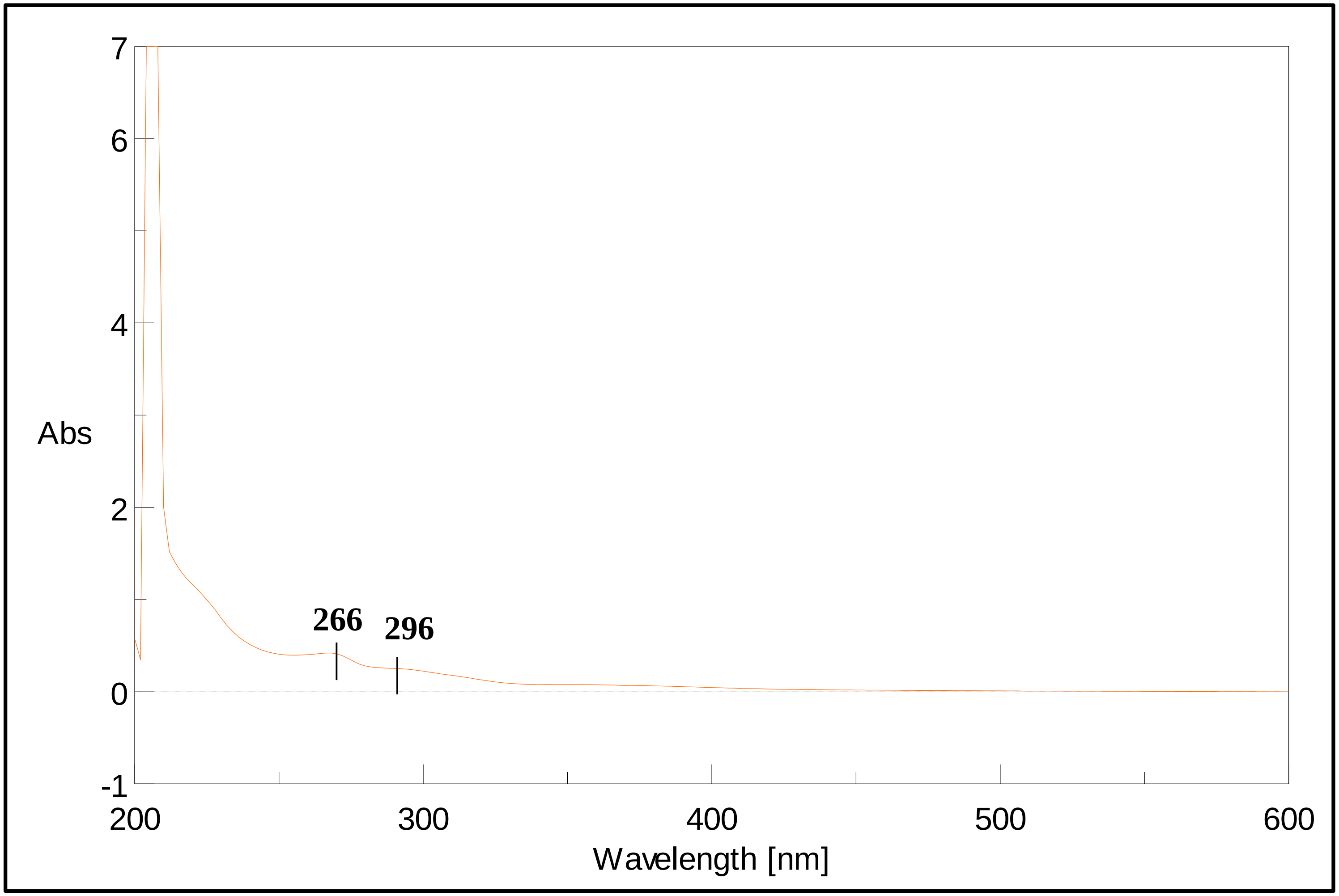

FIGURE S11: UV-spectrum of 3 in MeOH

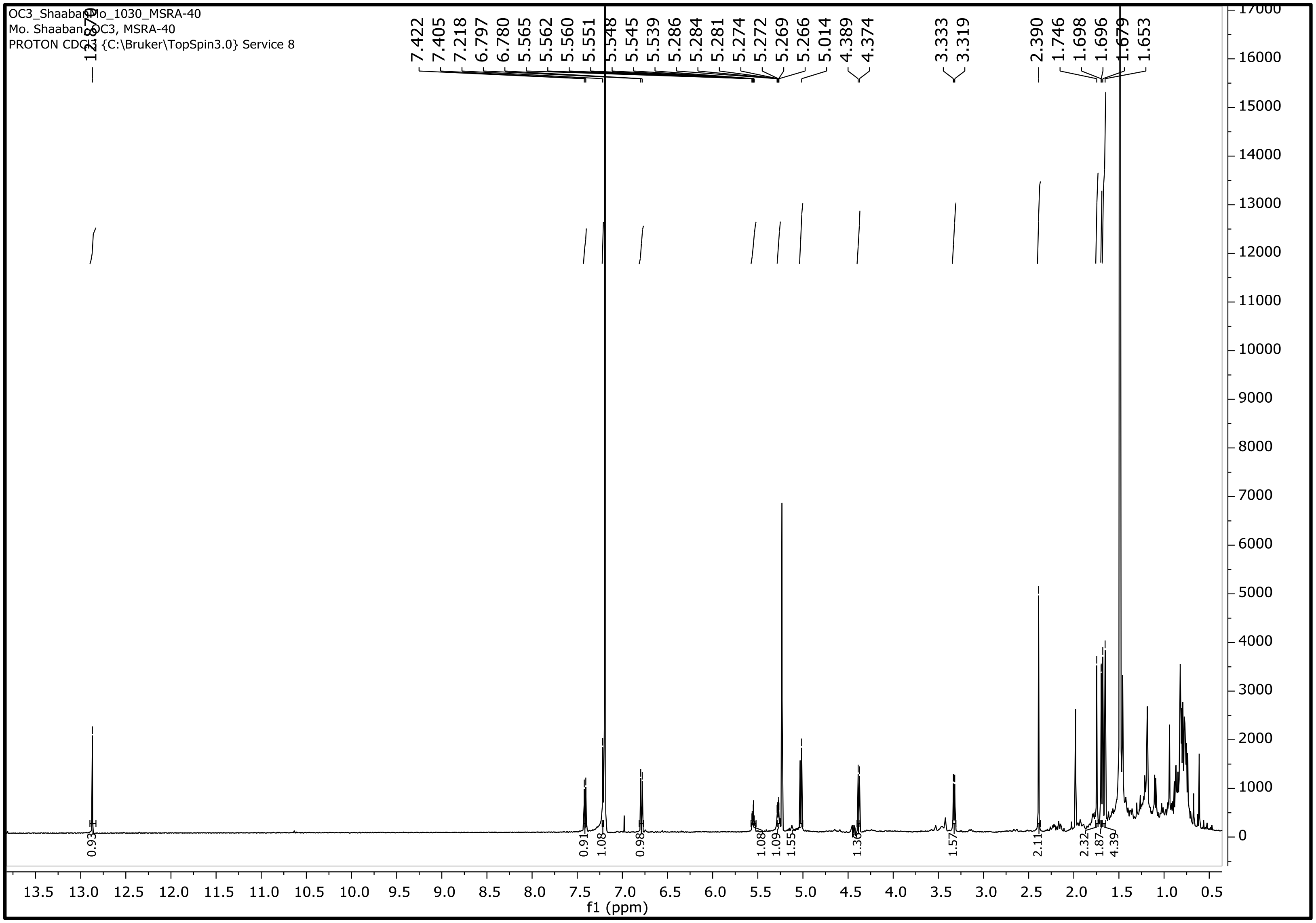

FIGURE S12: ¹H NMR spectrum of compound 3 ($CDCl_3$-*d*, 500 MHz)

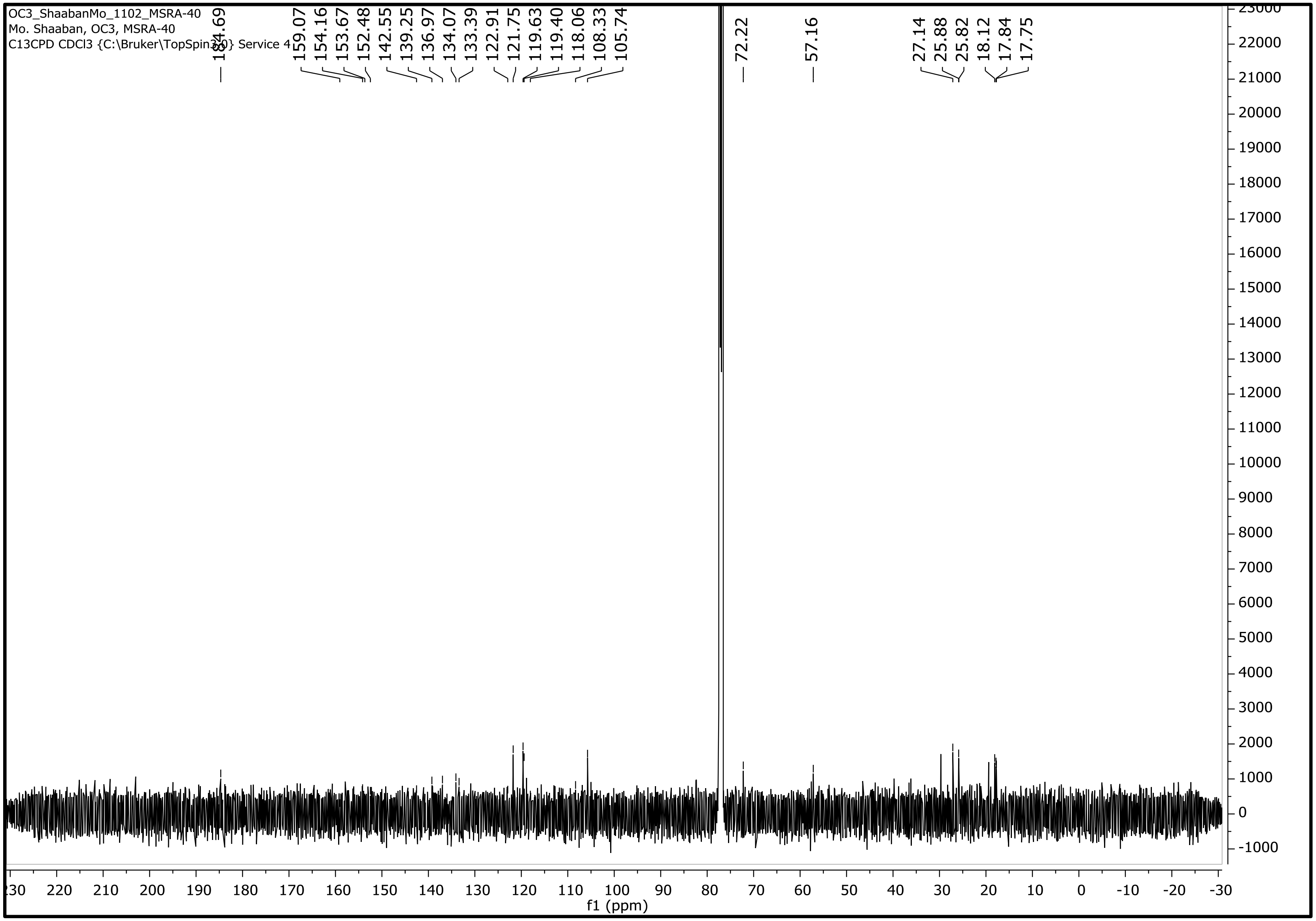

FIGURE S13: ^{13}C NMR spectrum of compound 3 ($CDCl_3$-*d*, 125

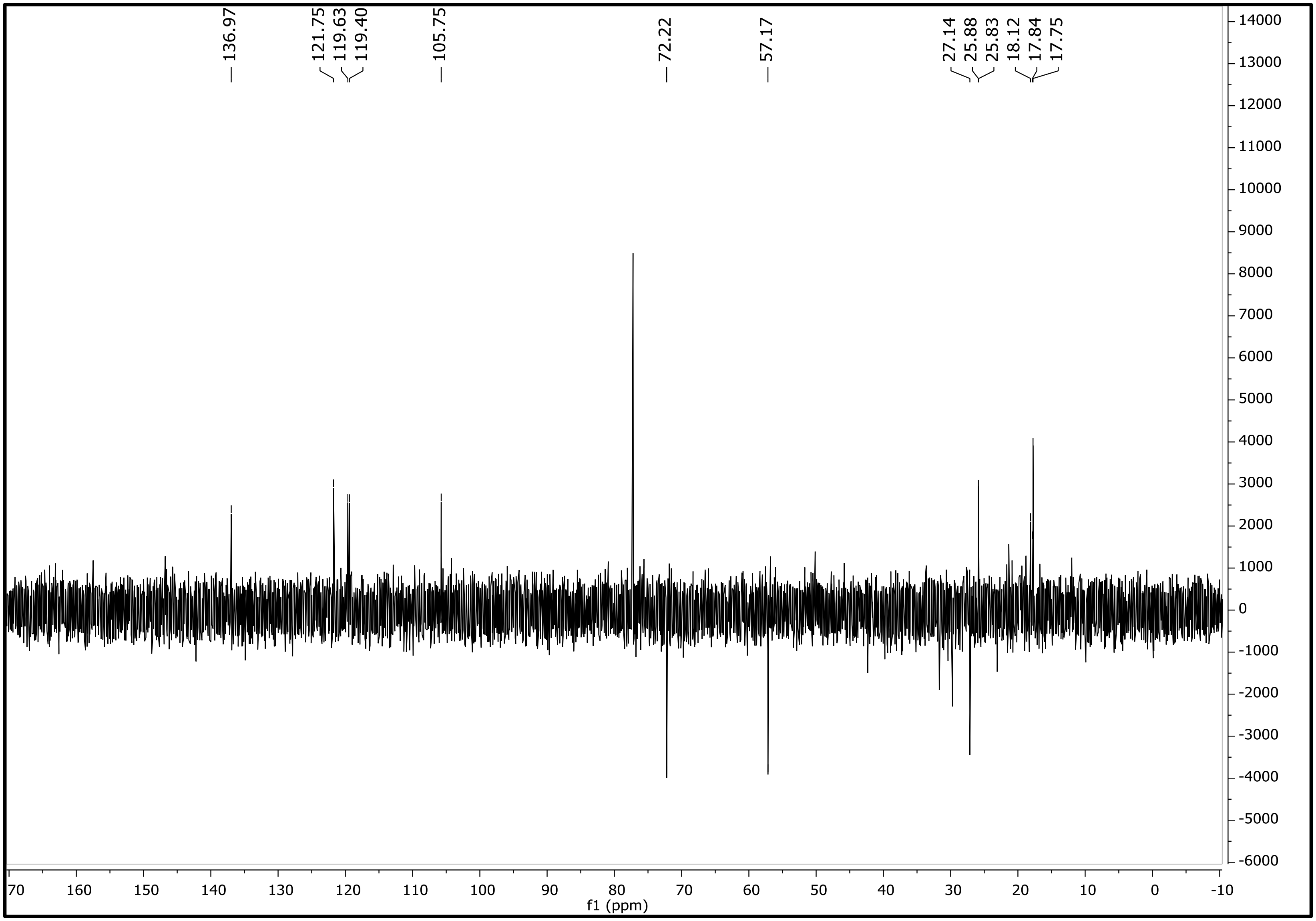

FIGURE S14: DEPT spectrum of compound 3 ($CDCl_3$-*d*, 125

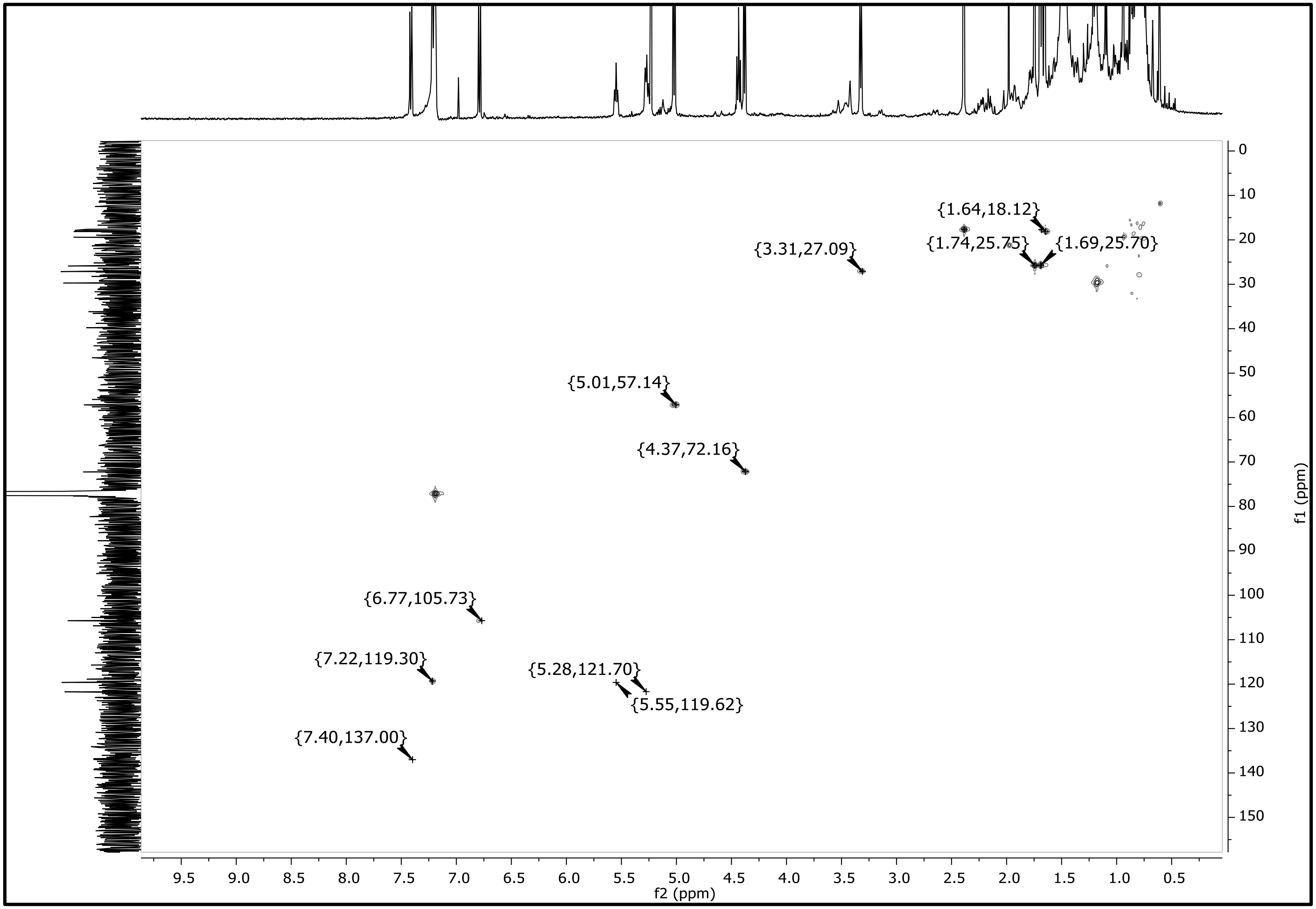

FIGURE S15: HMQC spectrum of compound 3 (CDCl$_3$-*d*, 500 MHz)

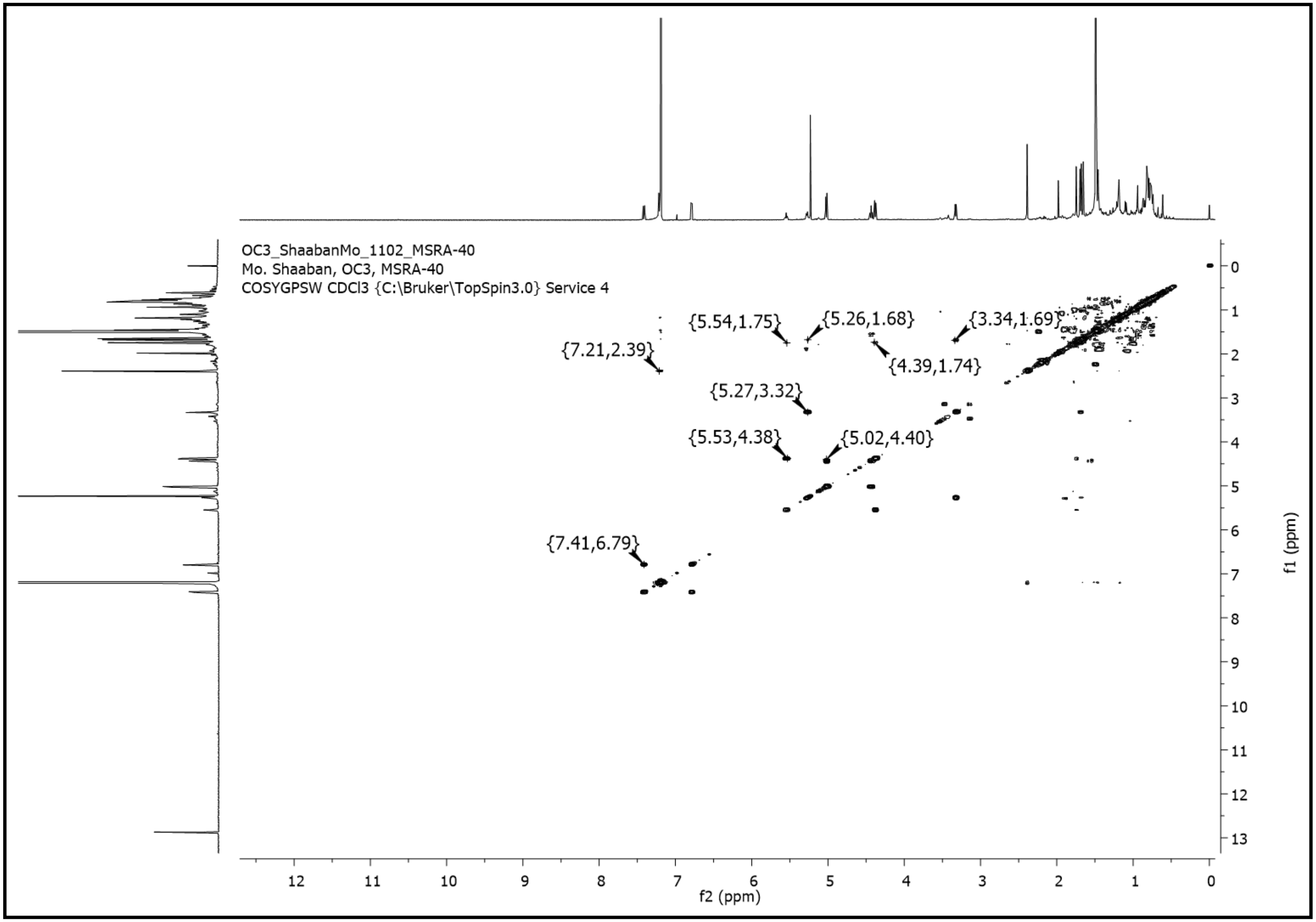

FIGURE S16: ^{1}H-^{1}H COSY spectrum of compound 3 ($CDCl_3$-*d*, 500

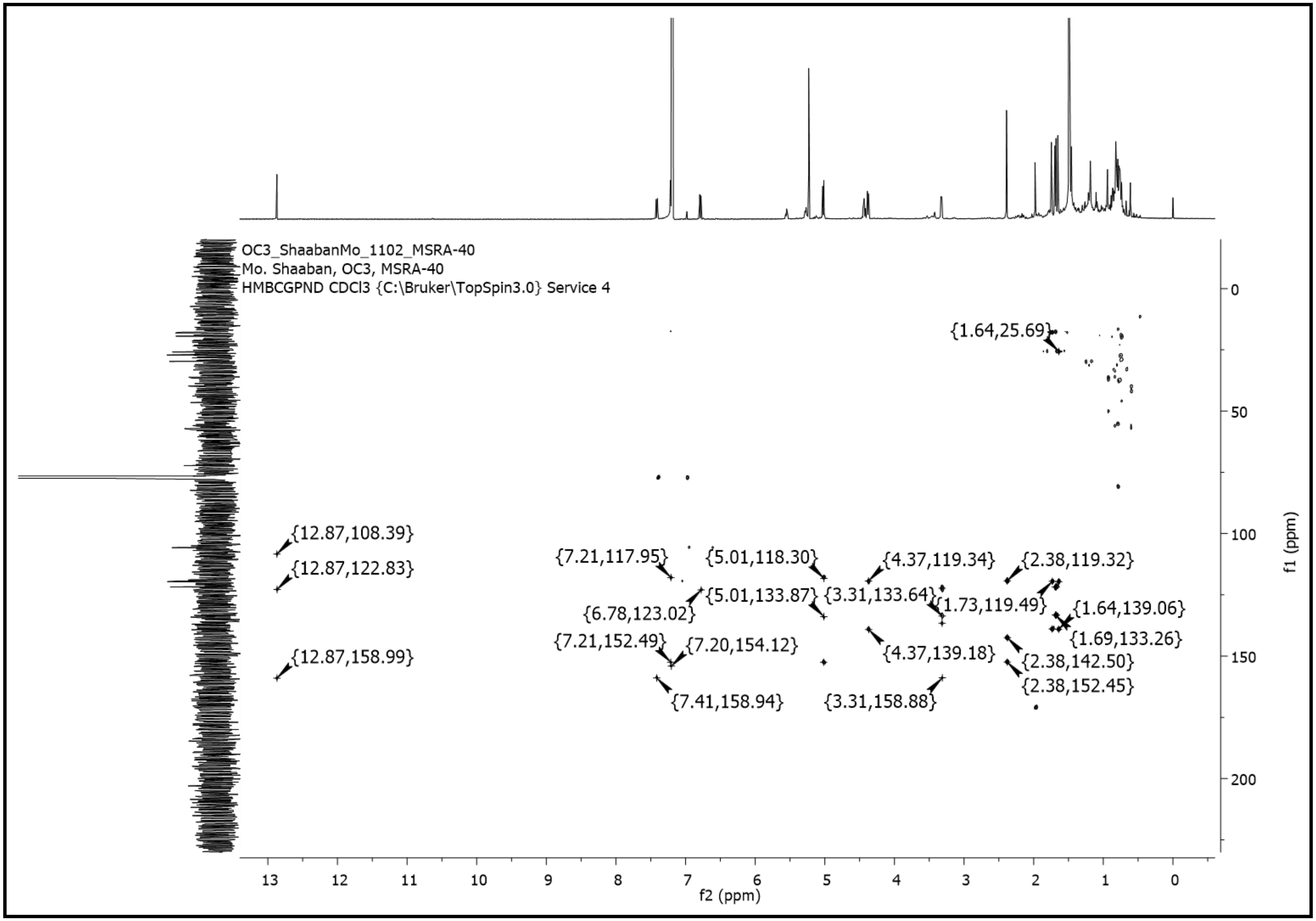

FIGURE S17: HMBC spectrum of compound 3 ($CDCl_3$-*d*, 500 MHz)

Analysis Report

Esquire 3000

Universität Bielefeld

Analysis Info

Analysis Name	OC3_ShaabanMo_1120_MSRA-40_01.d
Method	Tune-nan.MS
Workgroup	OC3
Comment	Mo. Shaaban, OC3, MSRA-40, ACN

Operator	S.Heitkamp
Acquisition Date	28.11.2017 09:39:58
Print Date	28.11.2017 10:41:13

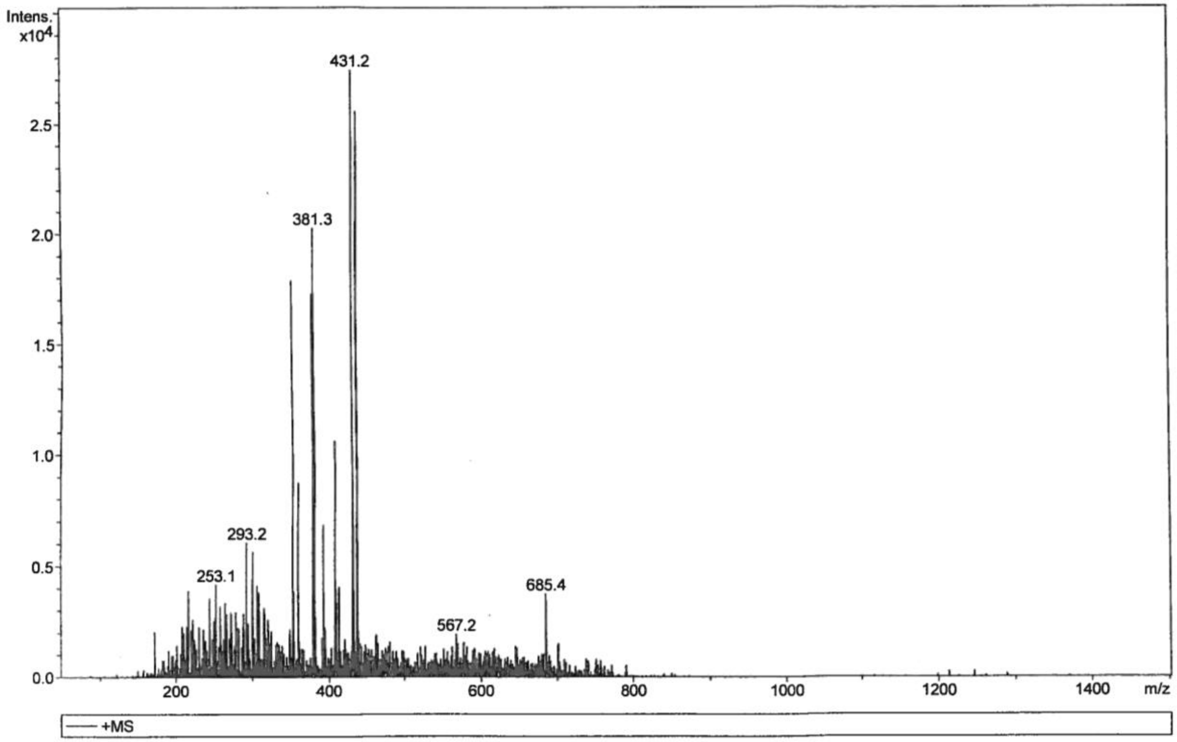

Acquisition Parameter

**Mode**	
Ion Source Type	NanoESI, off-line
Ion Polarity	Positive
Mass Range Mode	Std/Normal
Scan Begin	50 m/z
Scan End	1500 m/z
Averages	20 Spectra
Rolling	on
Rolling, Averages	3 cts
**Tune SPS**	
Target Mass	400 m/z
Compound Stability	40 %
Smart Parameter Setting	active
**Tune Instrument**	
Trap Drive	40.8
Octopole RF Amplitude	120.0 Vpp
Lens 2	-60.0 Volt
Capillary Exit	91.8 Volt
Skim 1	22.9 Volt
Skim 2	6.0 Volt
Lens 1	-5.0 Volt
Cap Exit Offset	68.9 Volt
Octopole	2.54 Volt
Octopole Delta	2.40 Volt
Dry Temp (Set)	100 °C
Nebulizer (Set)	2.00 psi
Dry Gas (Set)	2.00 l/min
Accumulation Time	2136 µs
ICC Target	5000
Charge Control	on

FIGURE S18: Positive ESI-MS spectrum of compound 3

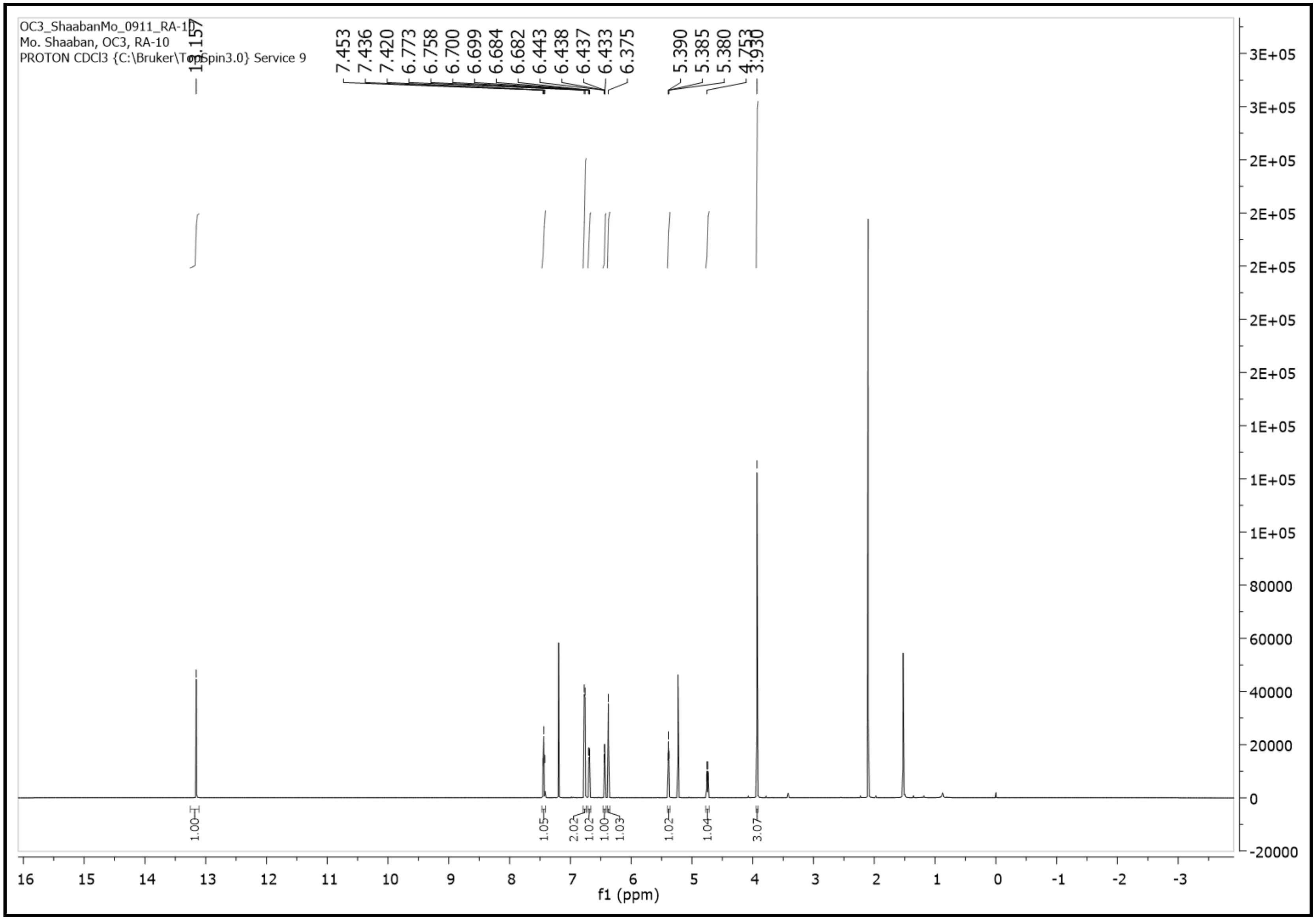

FIGURE S19: ^{1}H NMR spectrum of compound 4 ($CDCl_3$-*d*, 500 MHz)

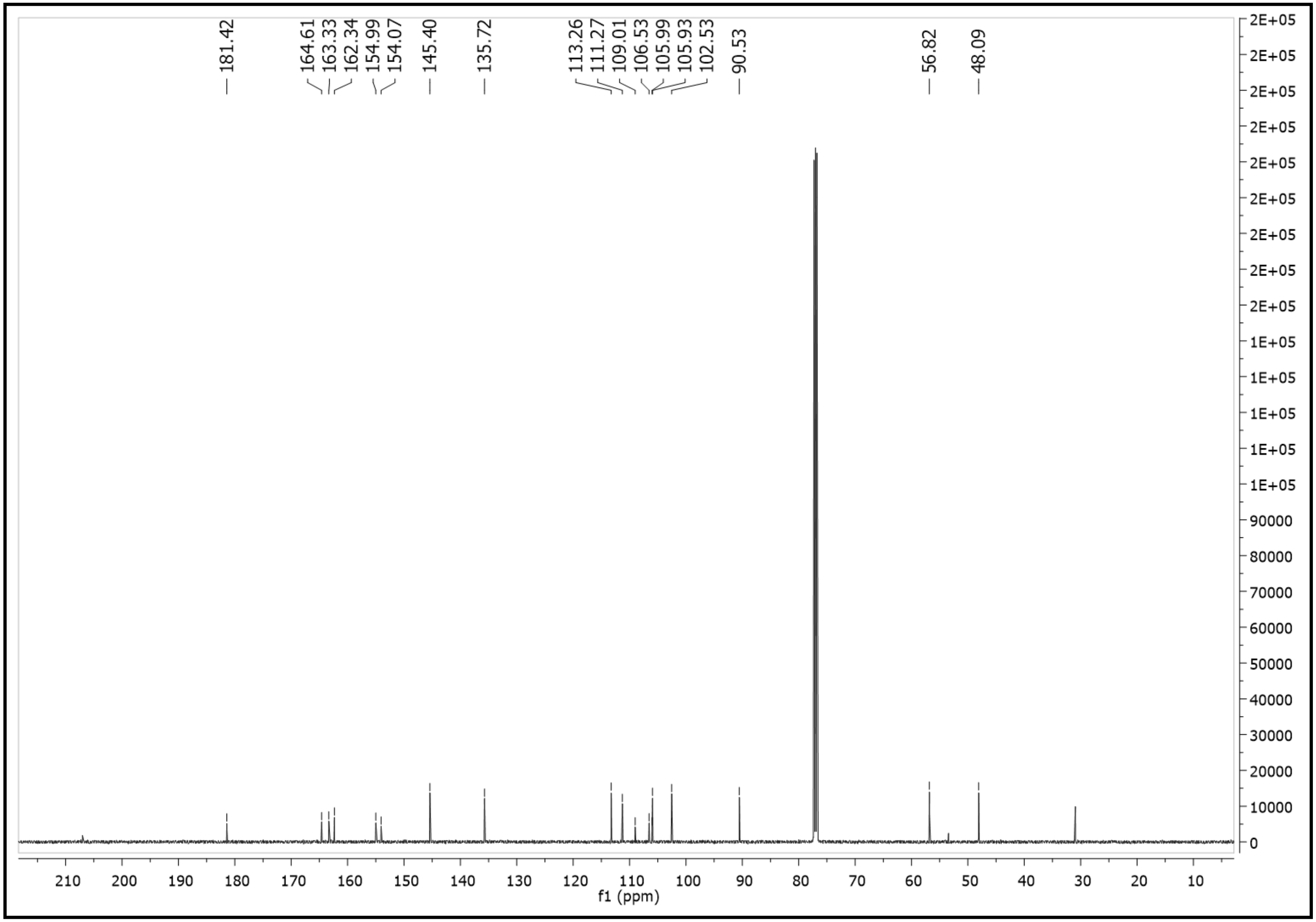

FIGURE S20: ^{13}C NMR spectrum of compound 4 ($CDCl_3$-*d*, 125 MHz)

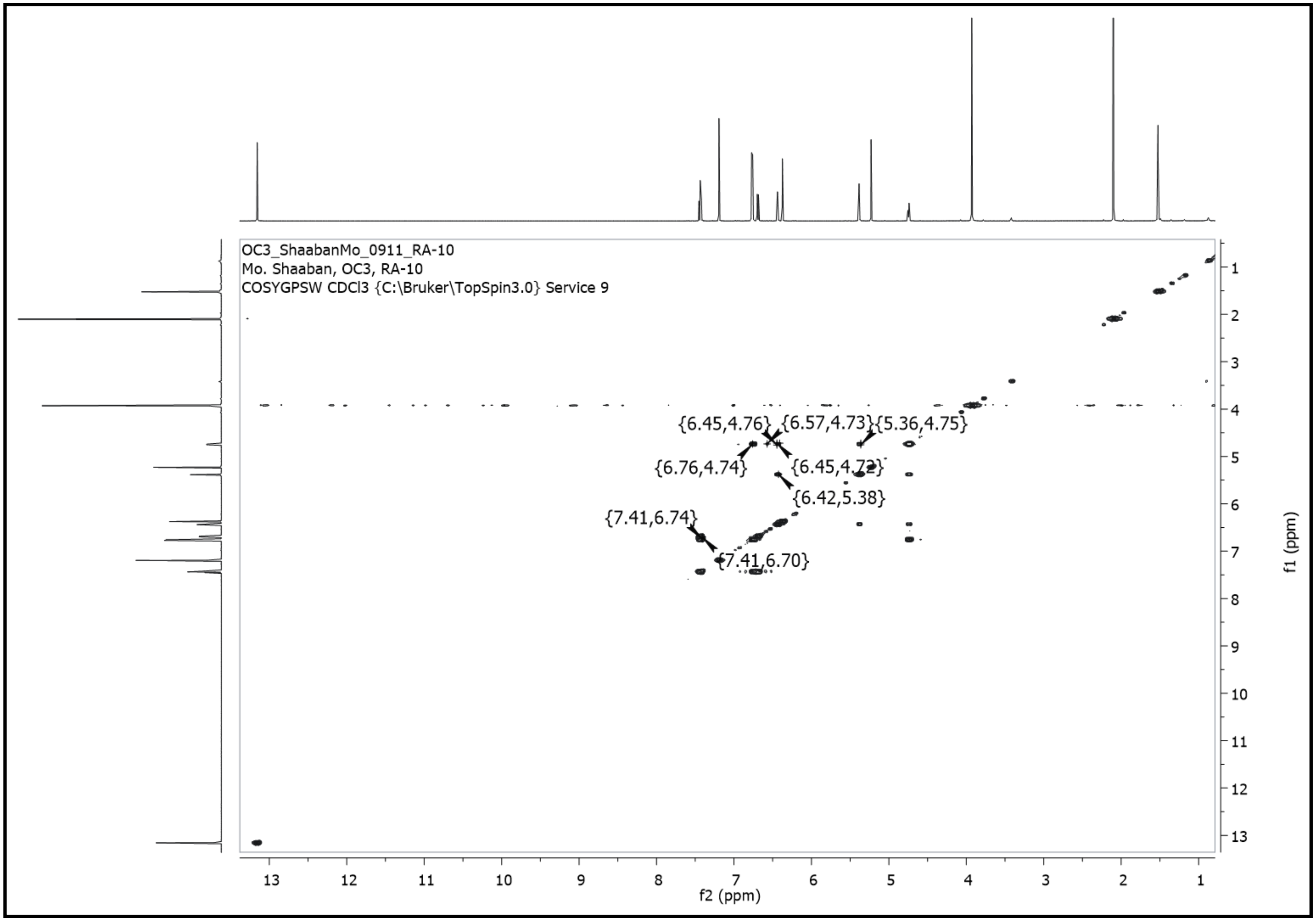

FIGURE S21: ^{1}H-^{1}H COSY spectrum of compound 4 ($CDCl_3$-*d*, 500 MHz)

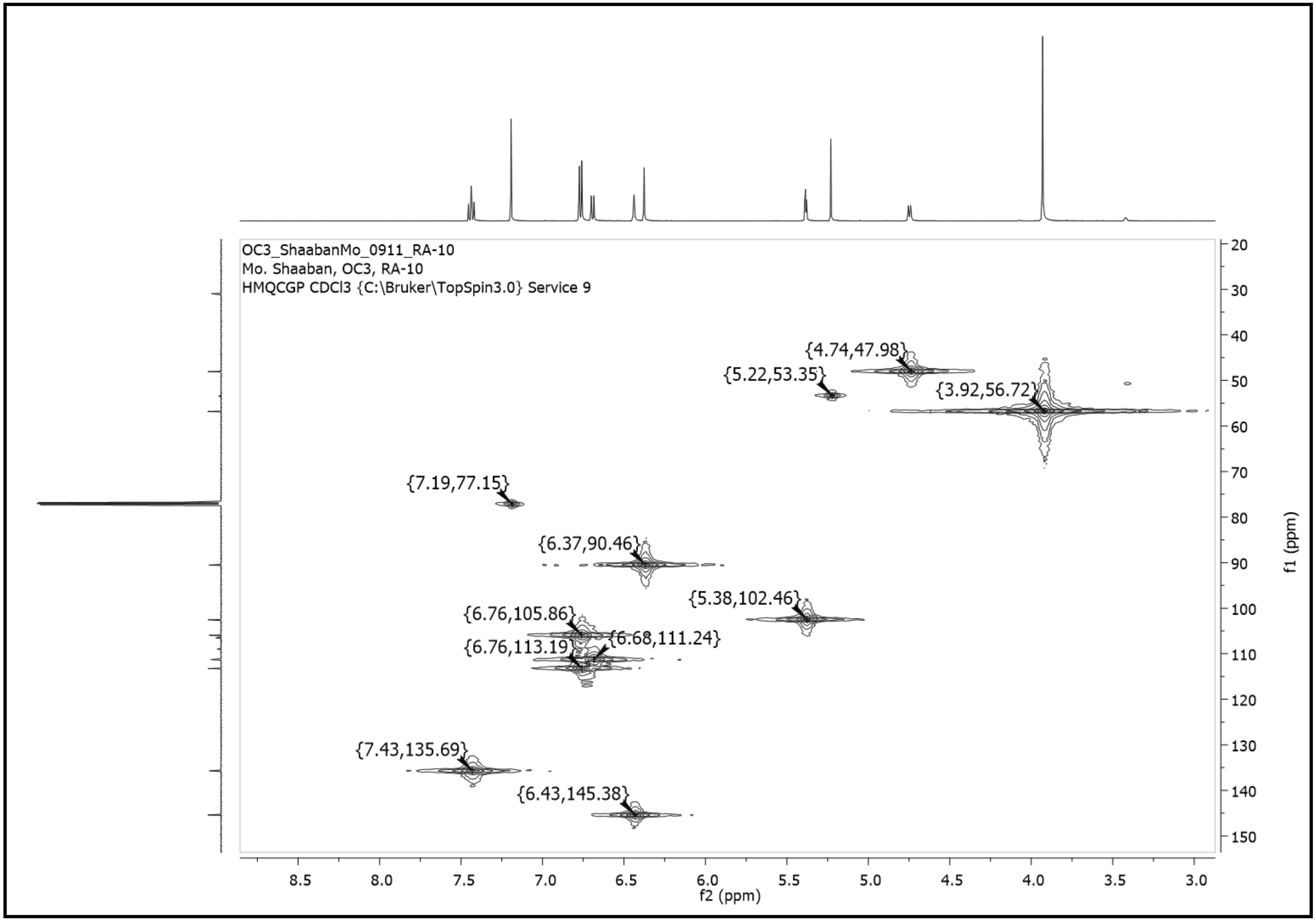

FIGURE S22: HMQC spectrum of compound 4 ($CDCl_3$-*d*, 500 MHz)

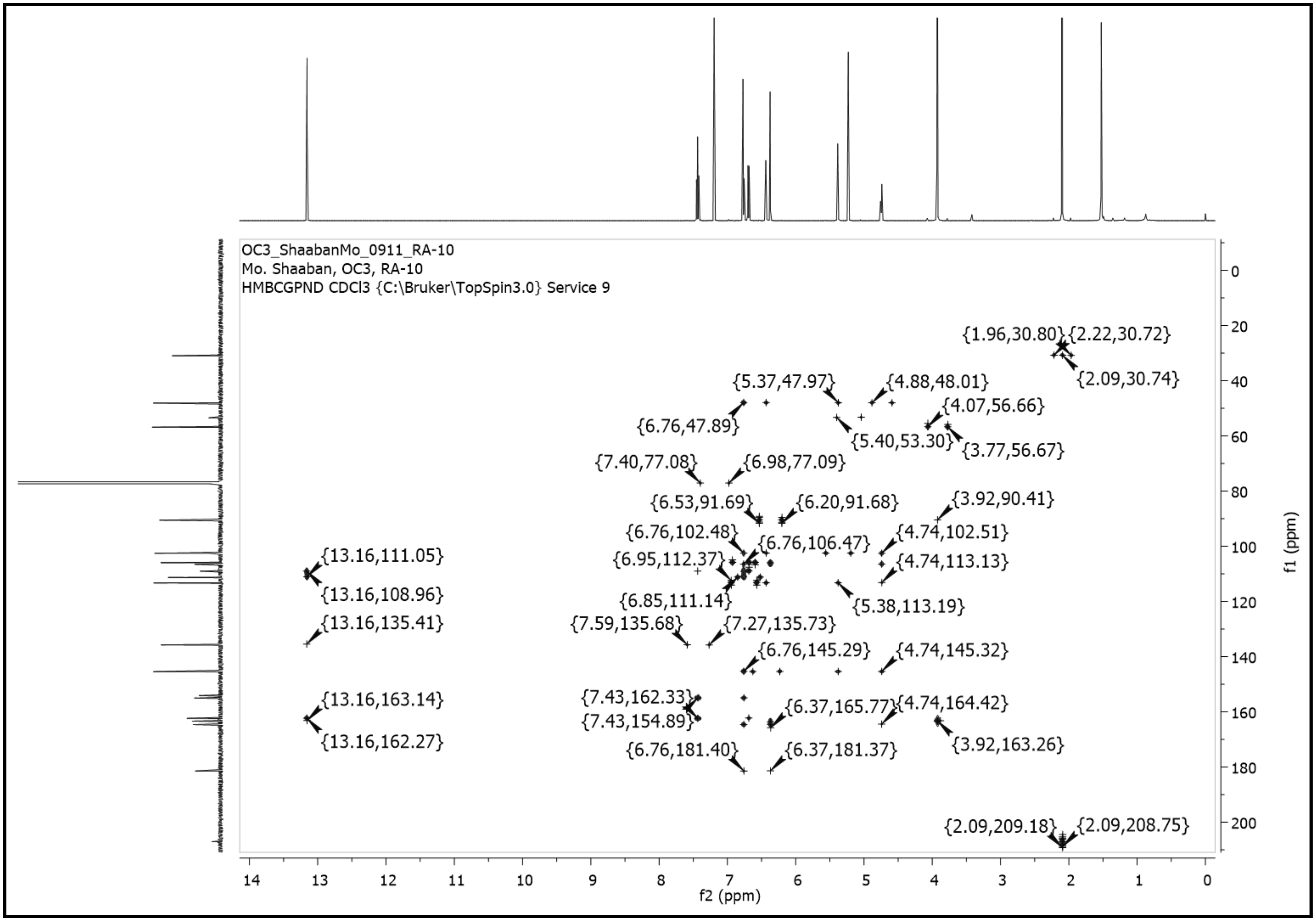

FIGURE S23: HMBC spectrum of compound 4 ($CDCl_3$-*d*, 500 MHz)

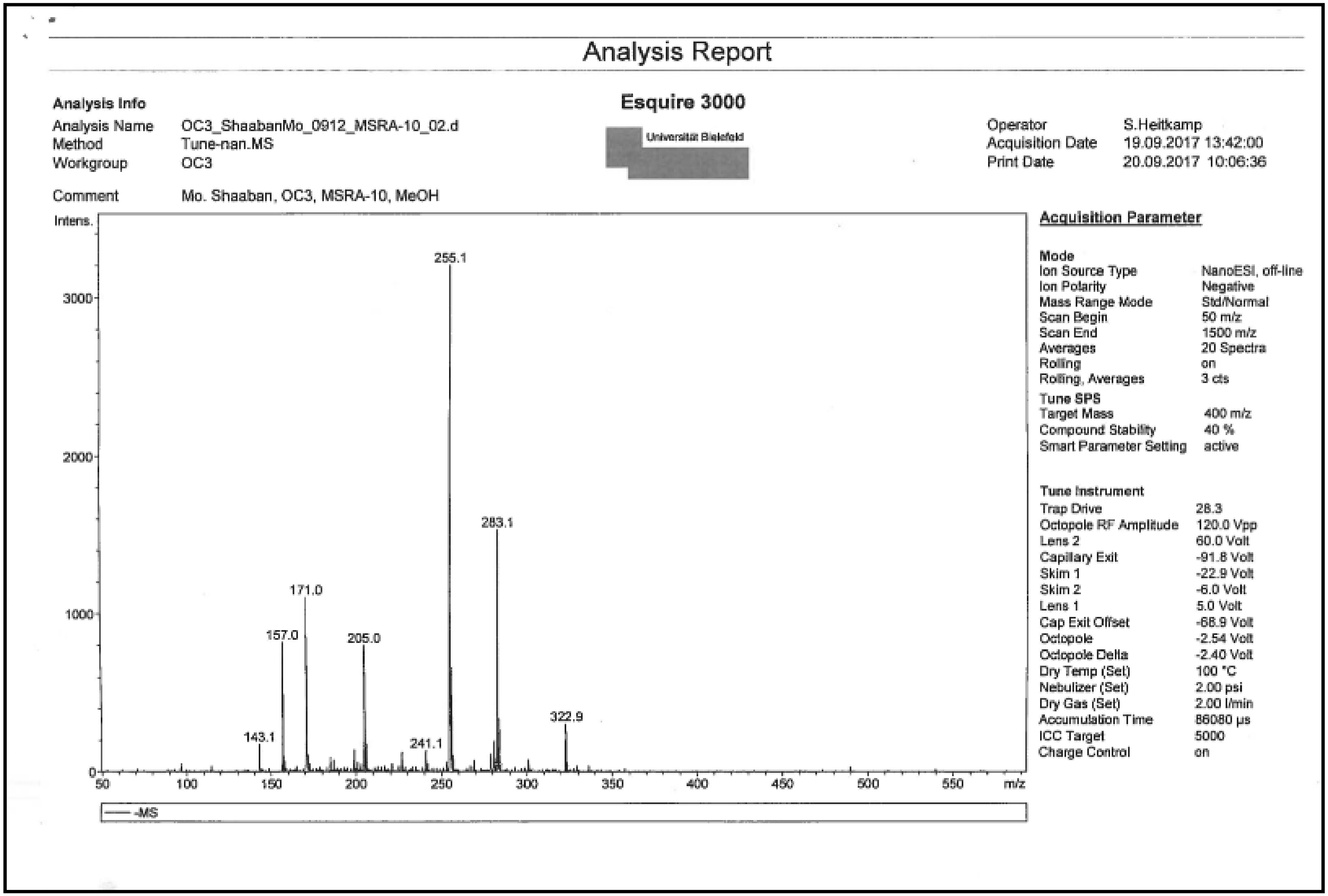

FIGURE S24: Negative ESI-MS spectrum of compound 4

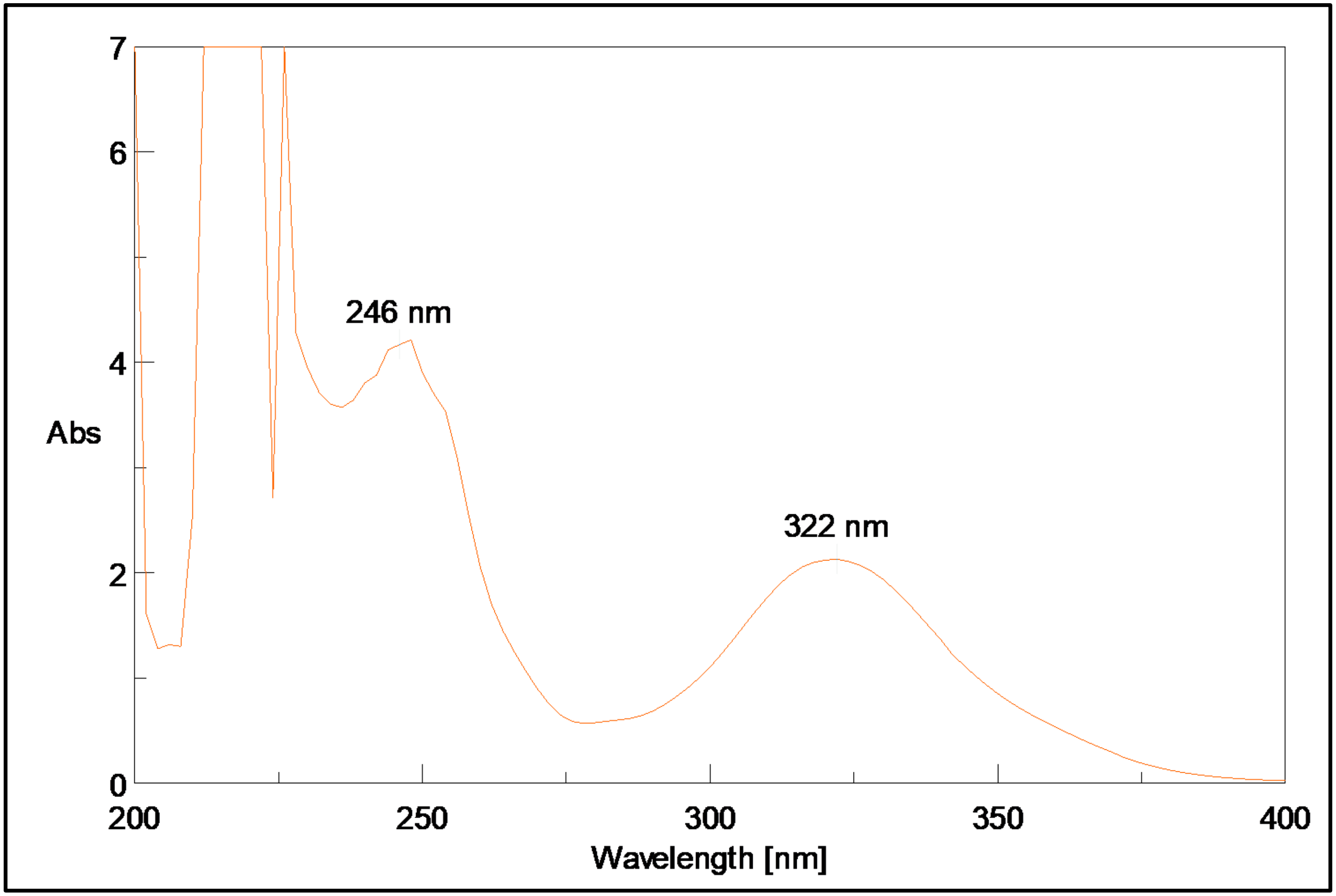

FIGURE S25: UV-spectrum of compound 5 in $CHCl_3$

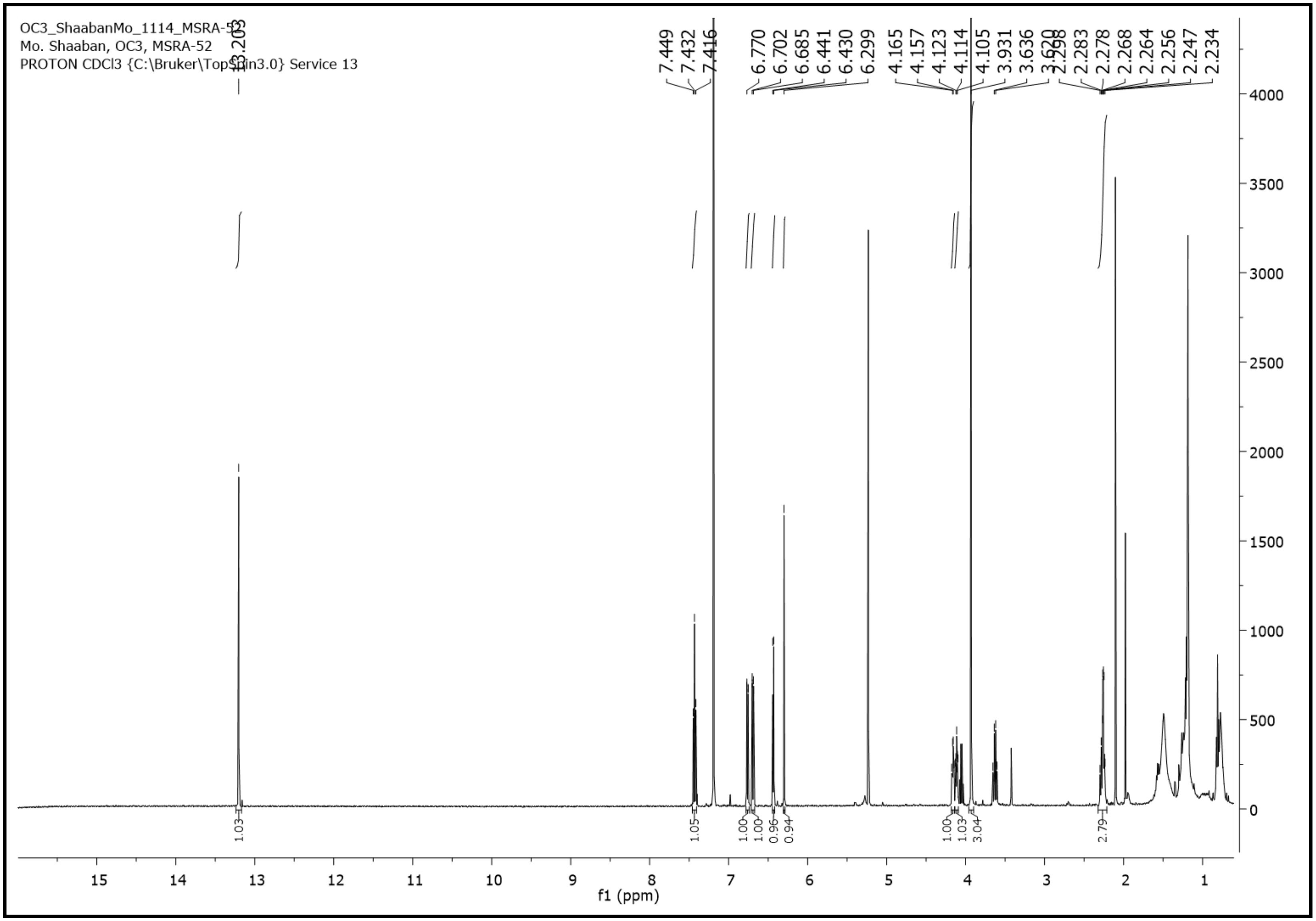

FIGURE S26: ^{1}H NMR spectrum of compound 5 ($CDCl_3$-*d*, 500 MHz)

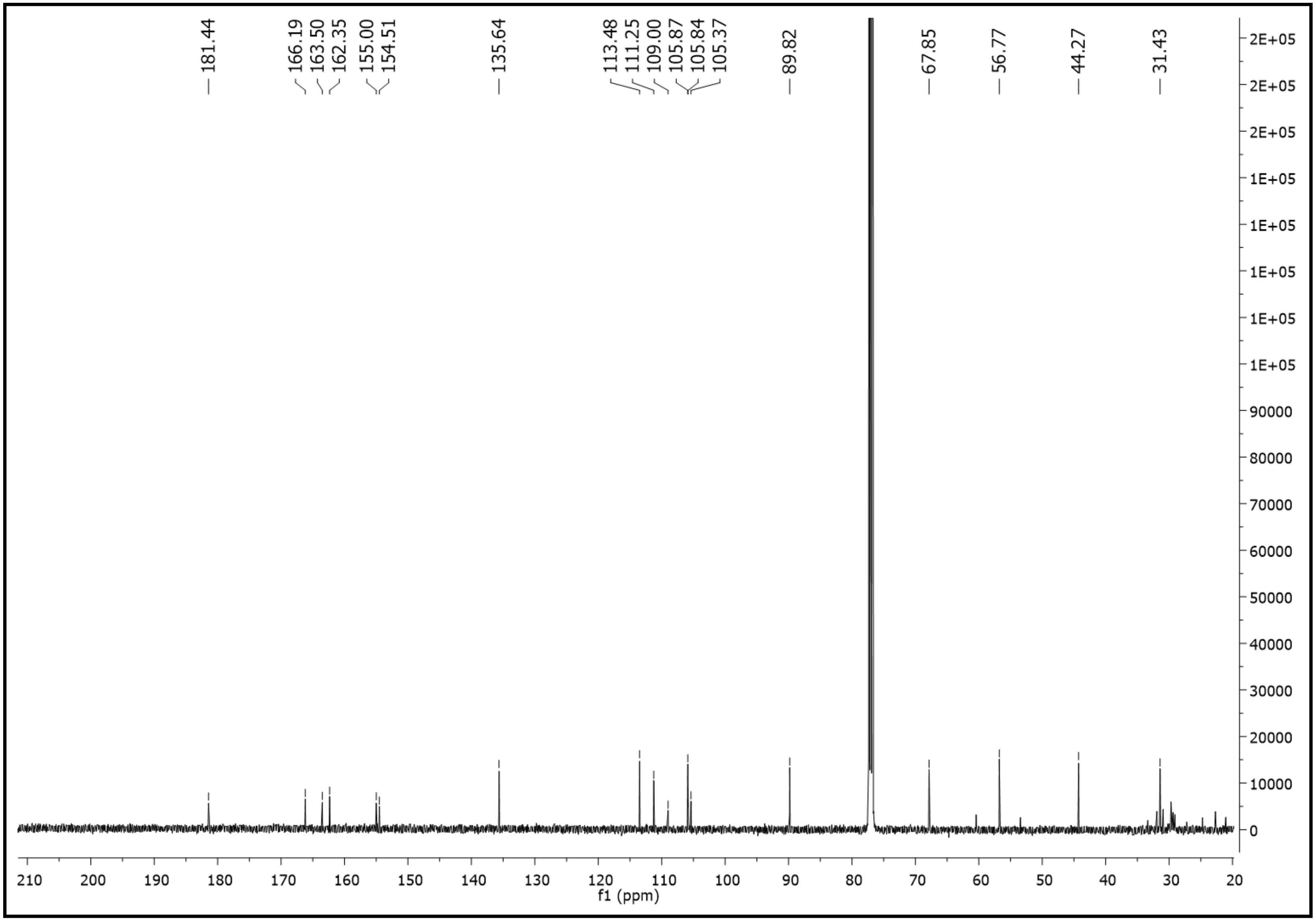

FIGURE S27: ^{13}C NMR spectrum of compound 5 ($CDCl_3$-*d*, 125

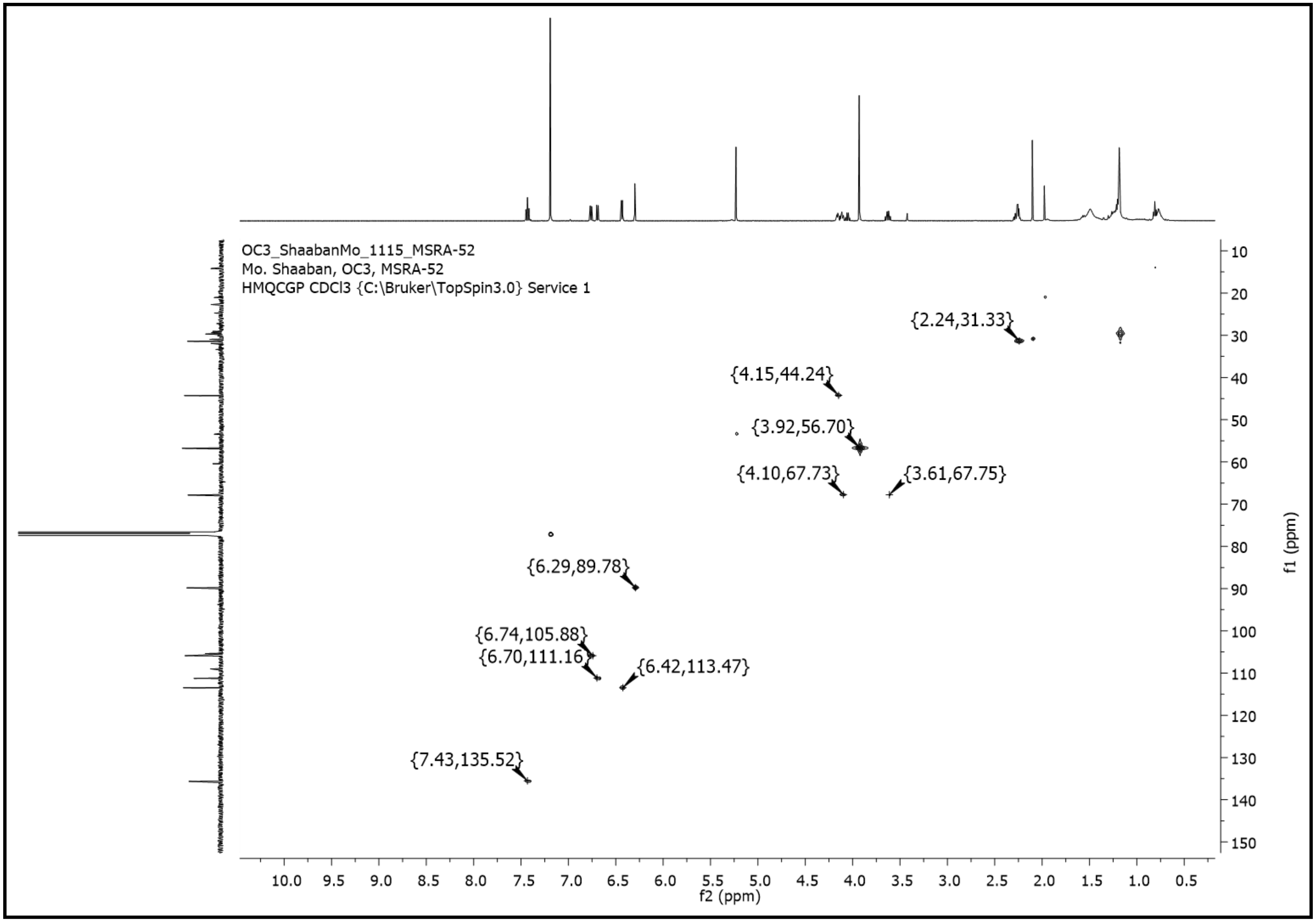

FIGURE S28: HMQC spectrum of compound 5 ($CDCl_3$-*d*, 500 MHz)

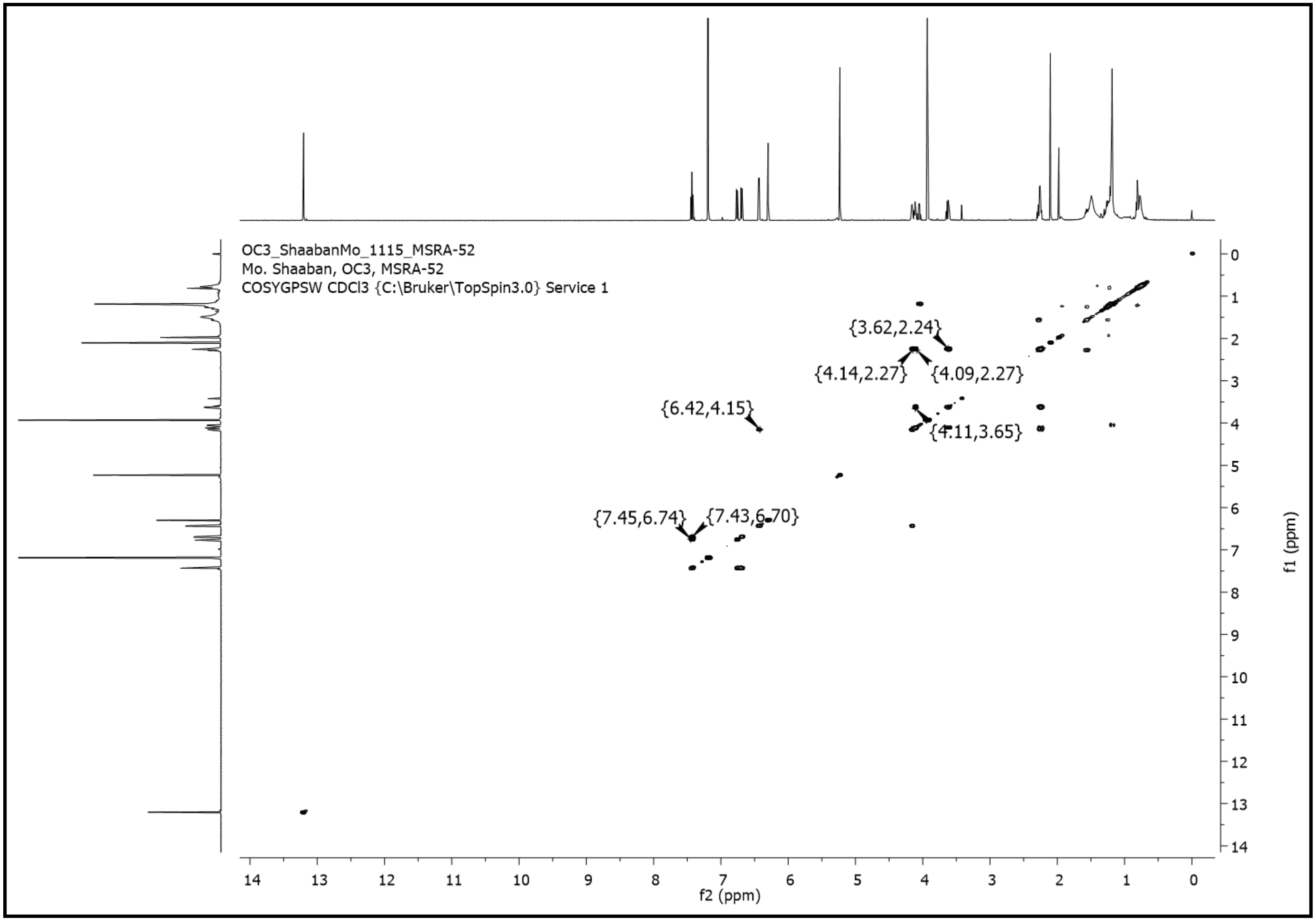

FIGURE S29: ^{1}H-^{1}H COSY spectrum of compound 5 ($CDCl_3$-*d*, 500

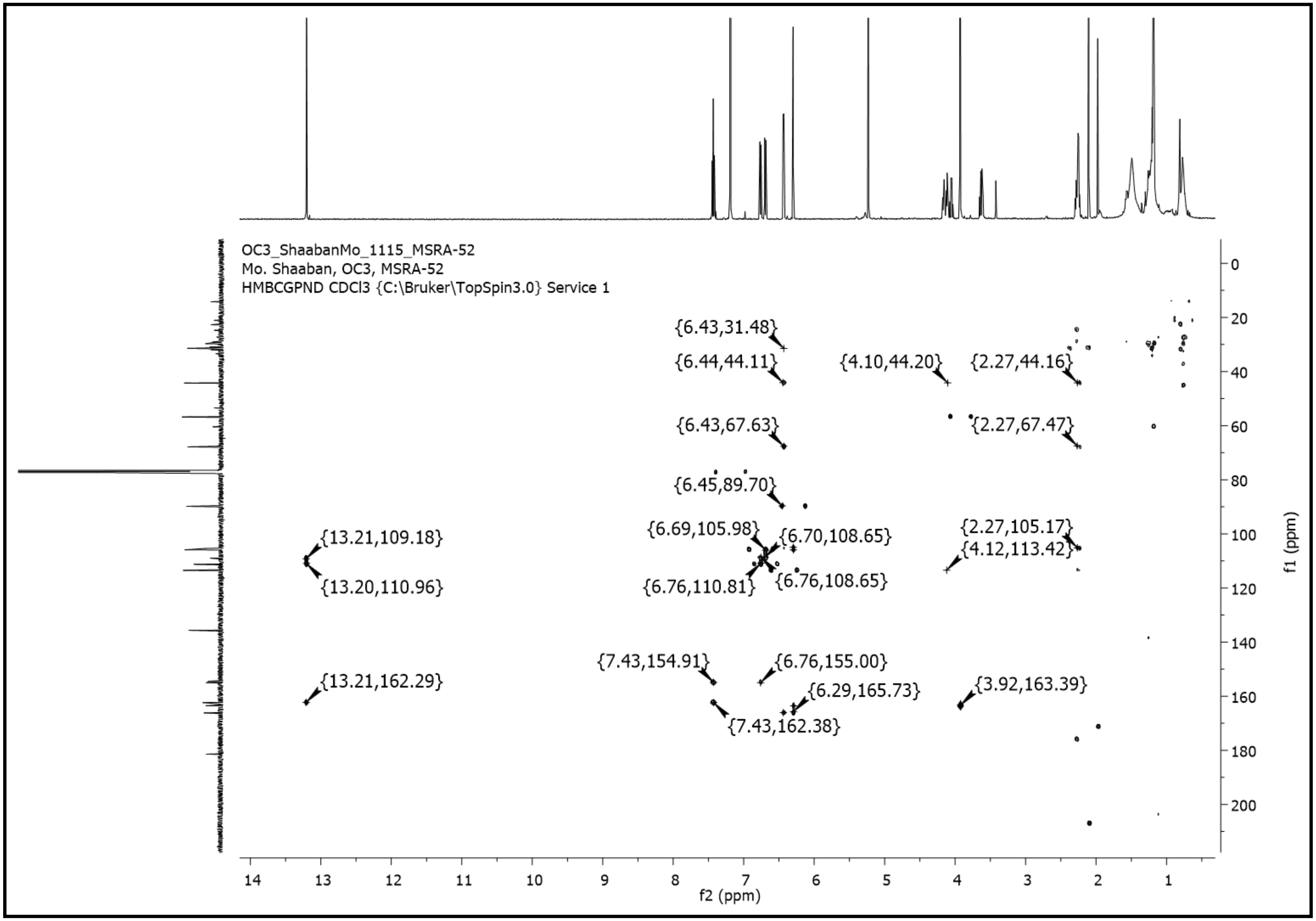

FIGURE S30: HMBC spectrum of compound 5 ($CDCl_3$-*d*, 500 MHz)

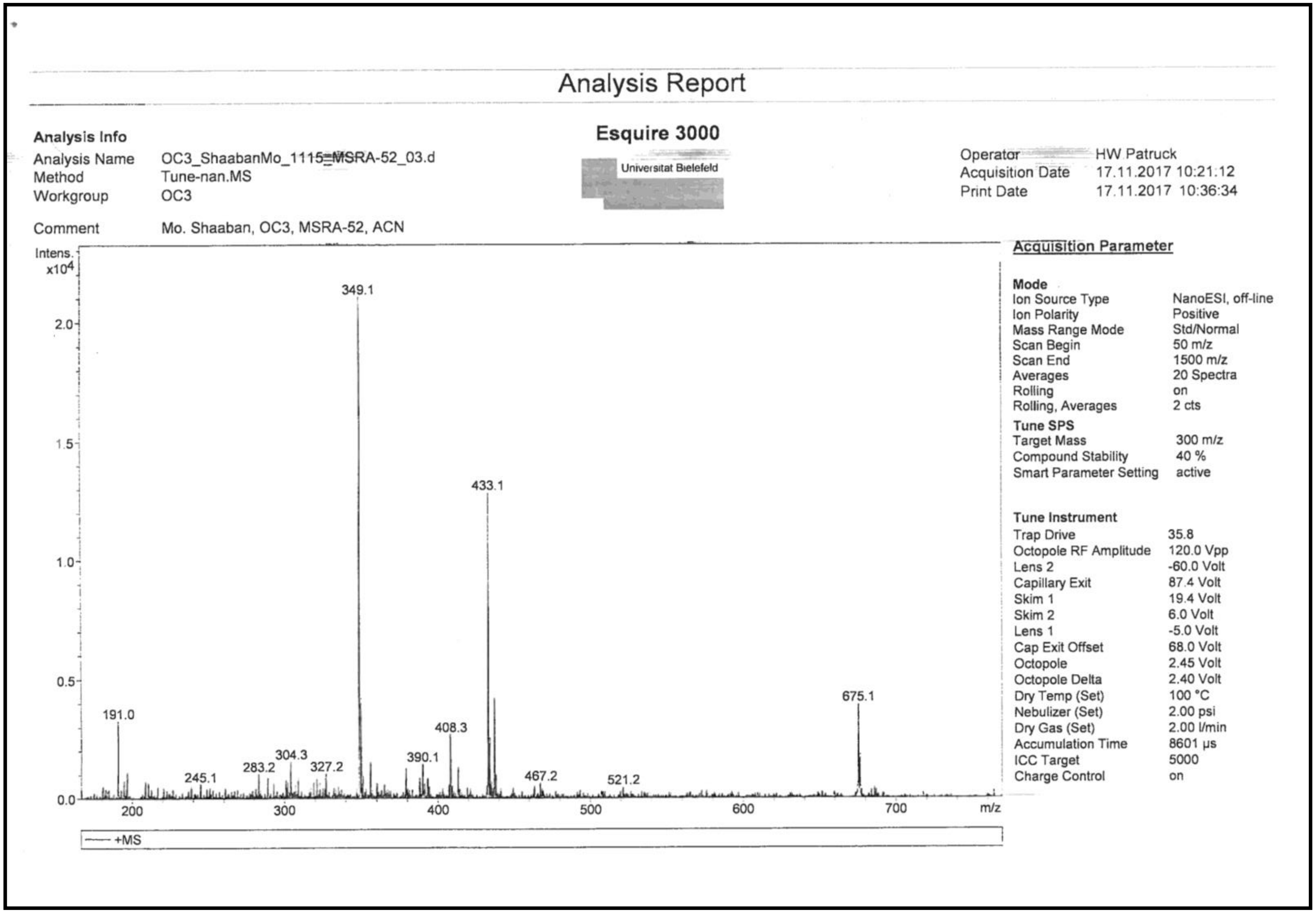

FIGURE S31: Negative ESI-MS spectrum of compound 5

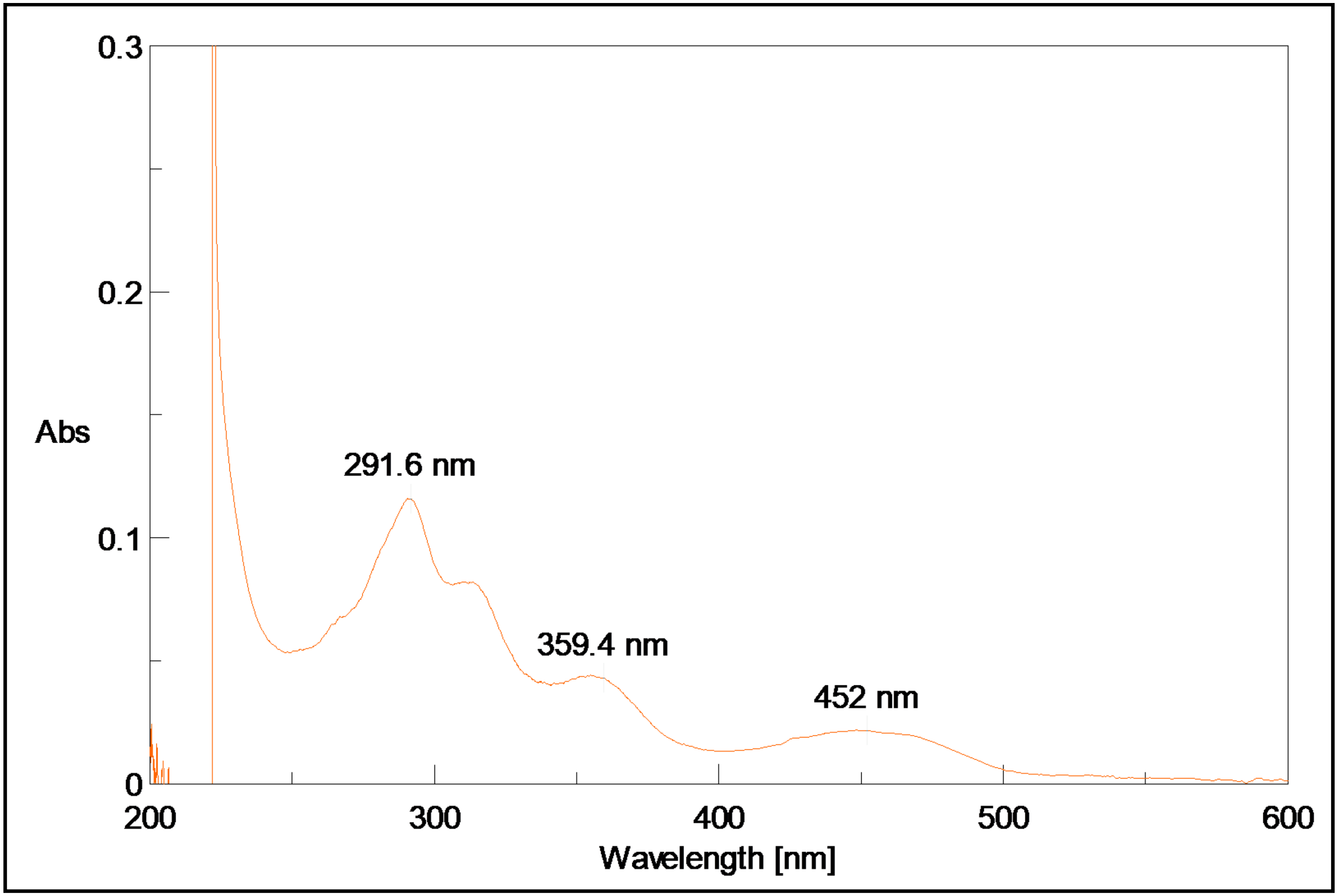

FIGURE S32: UV-spectrum of compound 6 in MeOH

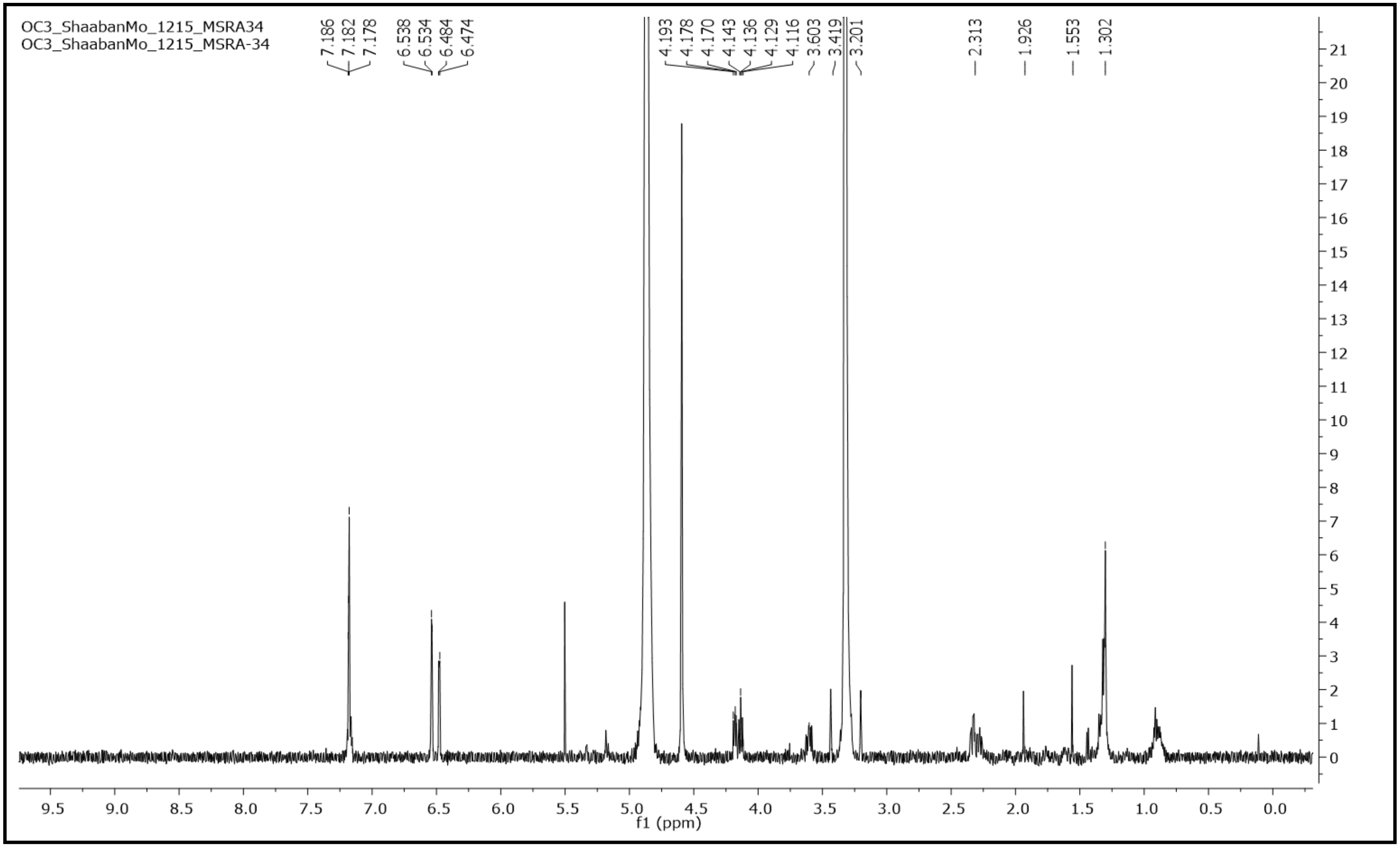

FIGURE S33: ^{1}H NMR spectrum of compound 6 (CD_3OD-d_4, 600

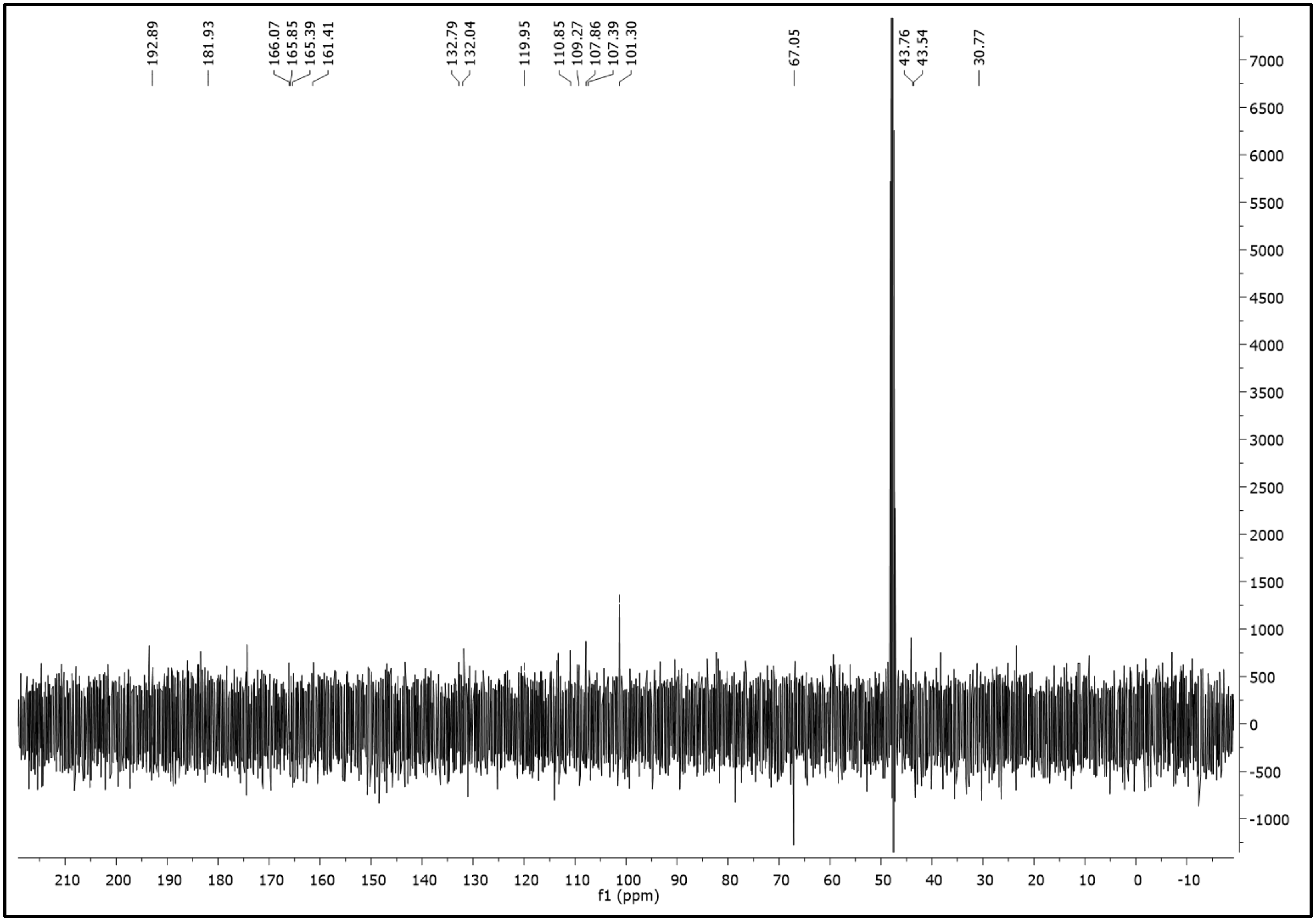

FIGURE S34: ^{13}C NMR spectrum of compound 6 (CD_3OD-d_4-*d*, 150

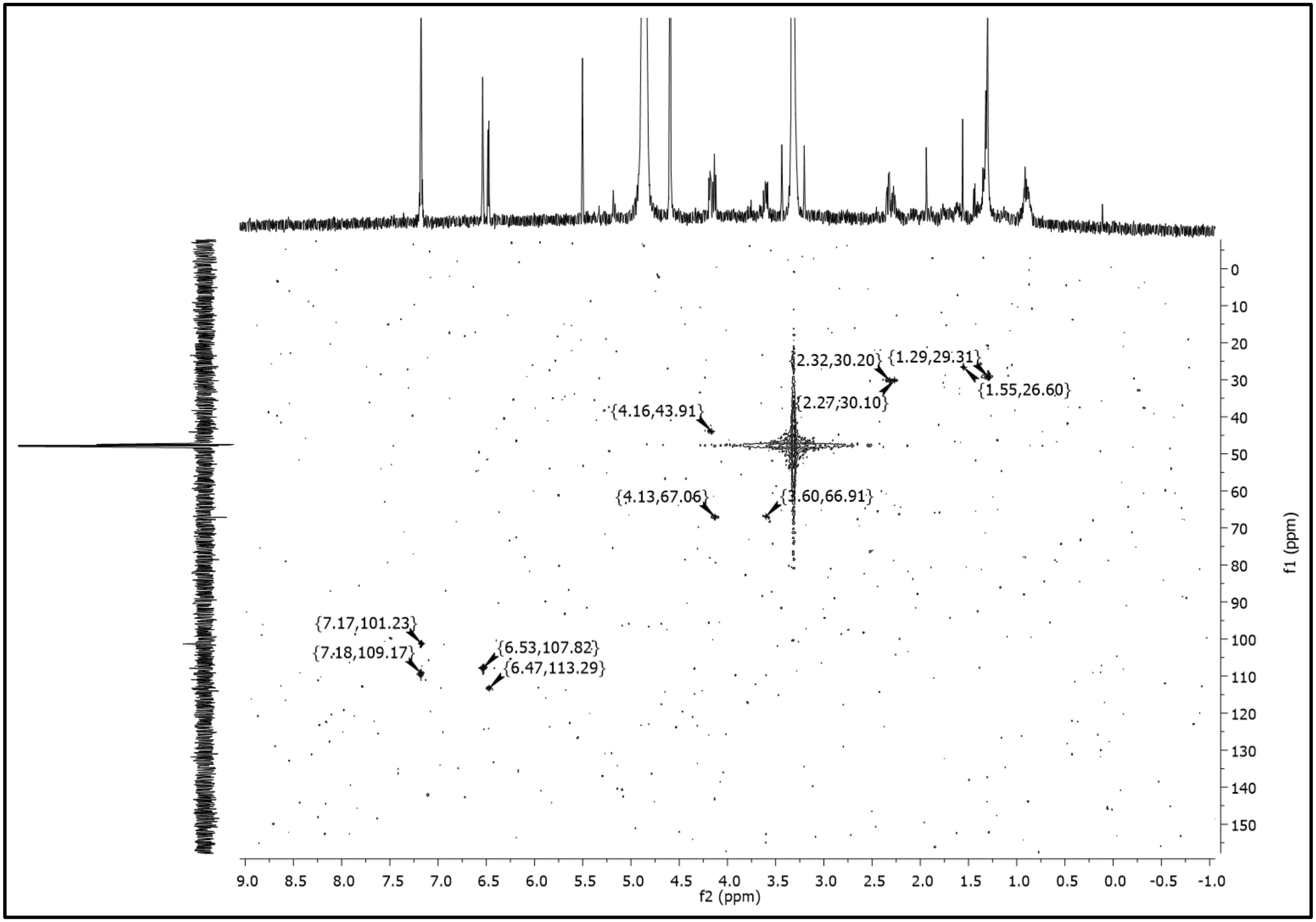

FIGURE S35: HMQC spectrum of compound 6 (CD_3OD-d_4, 600 MHz)

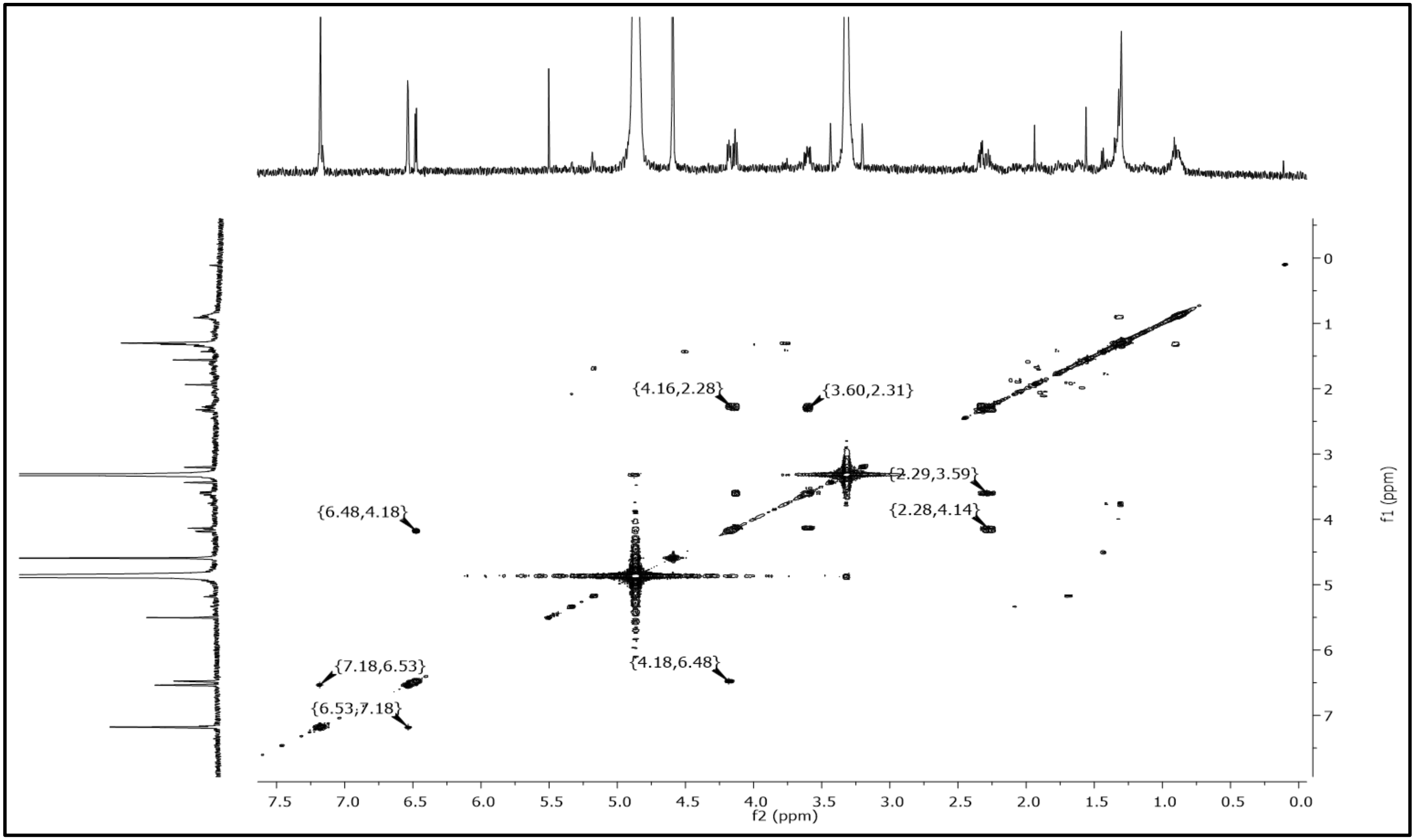

FIGURE S36:^{1}H-^{1}H COSY spectrum of compound 6 (CD_3OD-d_4, 600

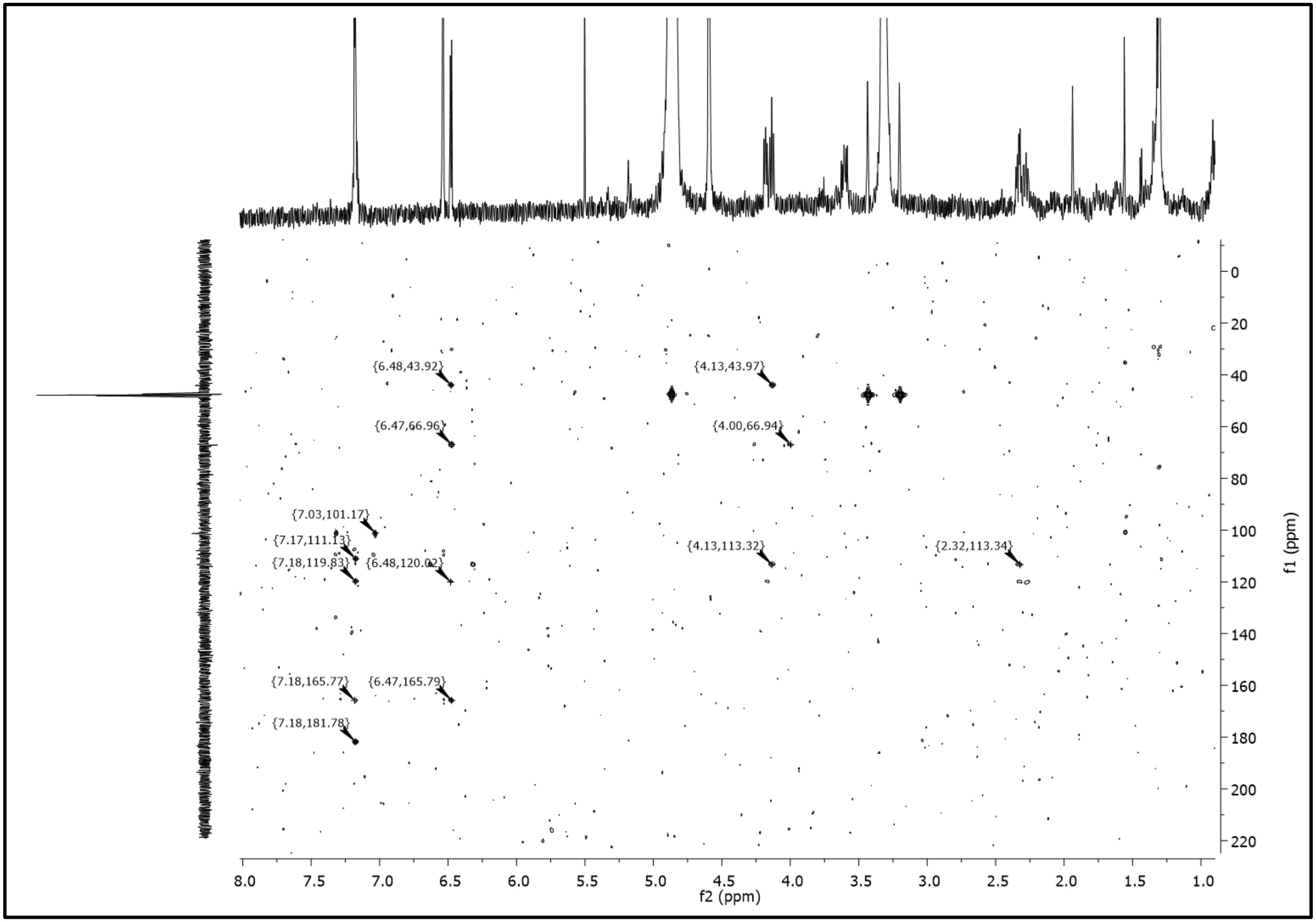

FIGURE S37: HMBC spectrum of compound 6 (CD_3OD-d_4, 600 MHz)

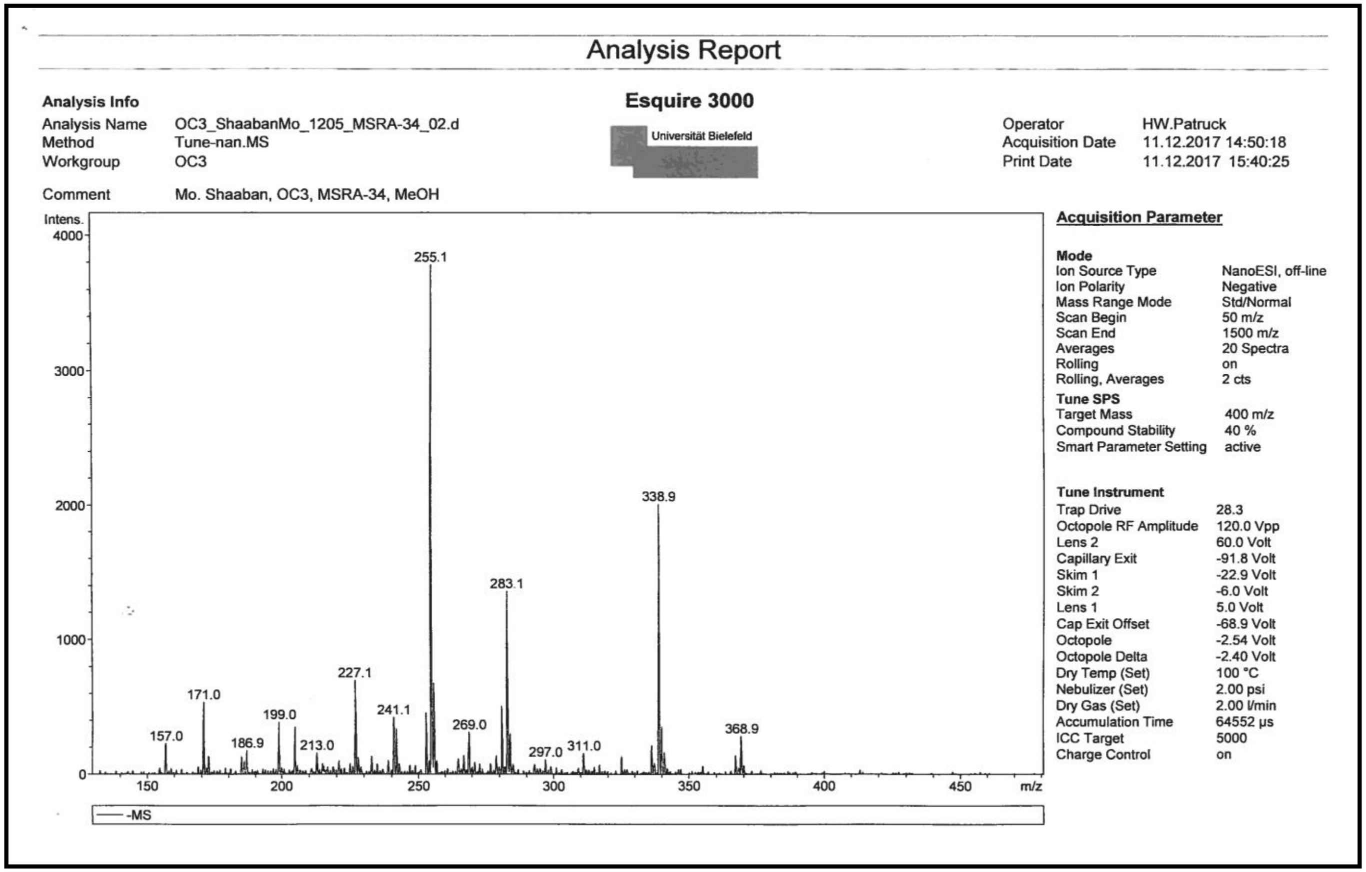

FIGURE S38: Negative ESI-MS spectrum of compound 5

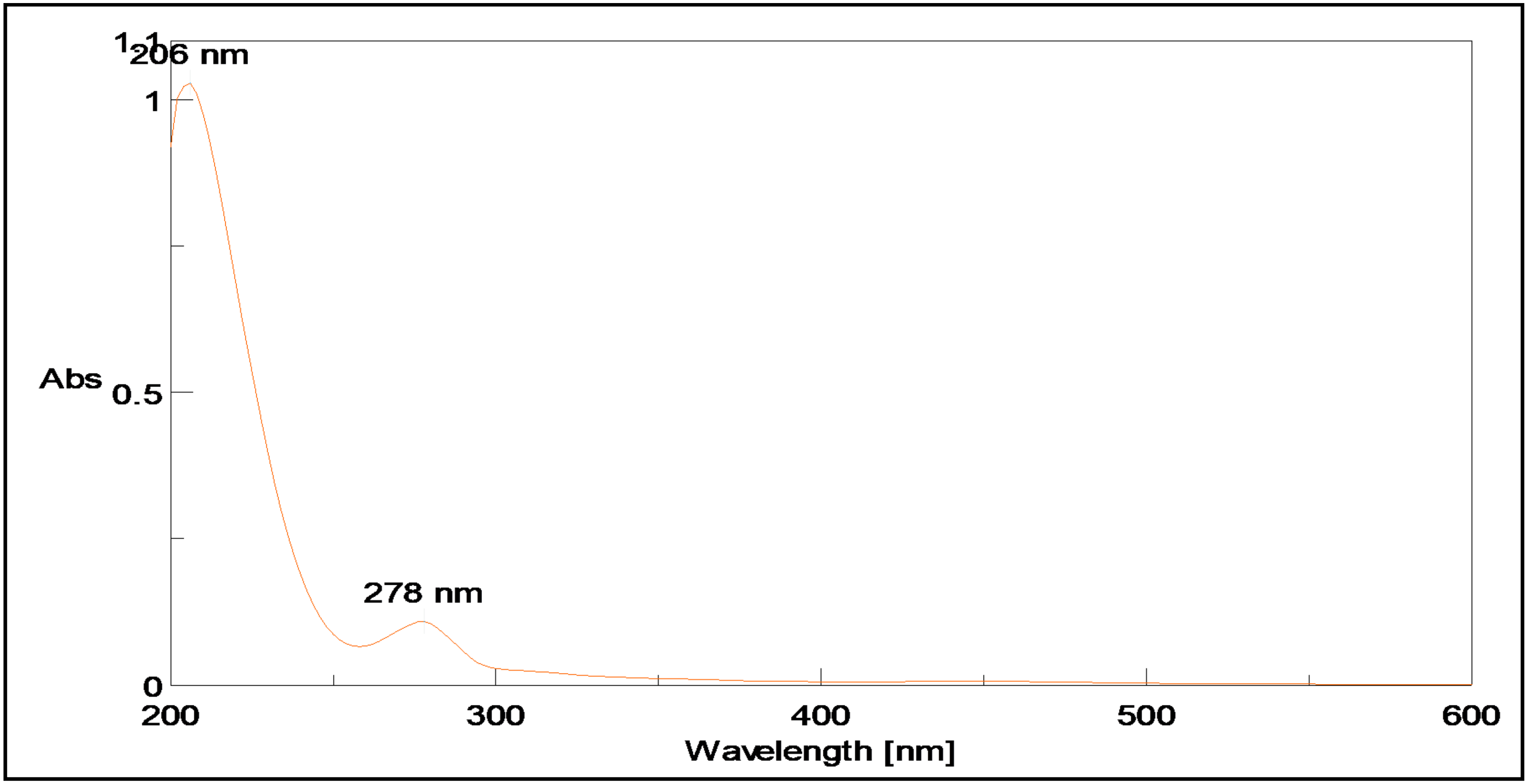

FIGURE S39: UV-spectrum of compound 7 in MeOH

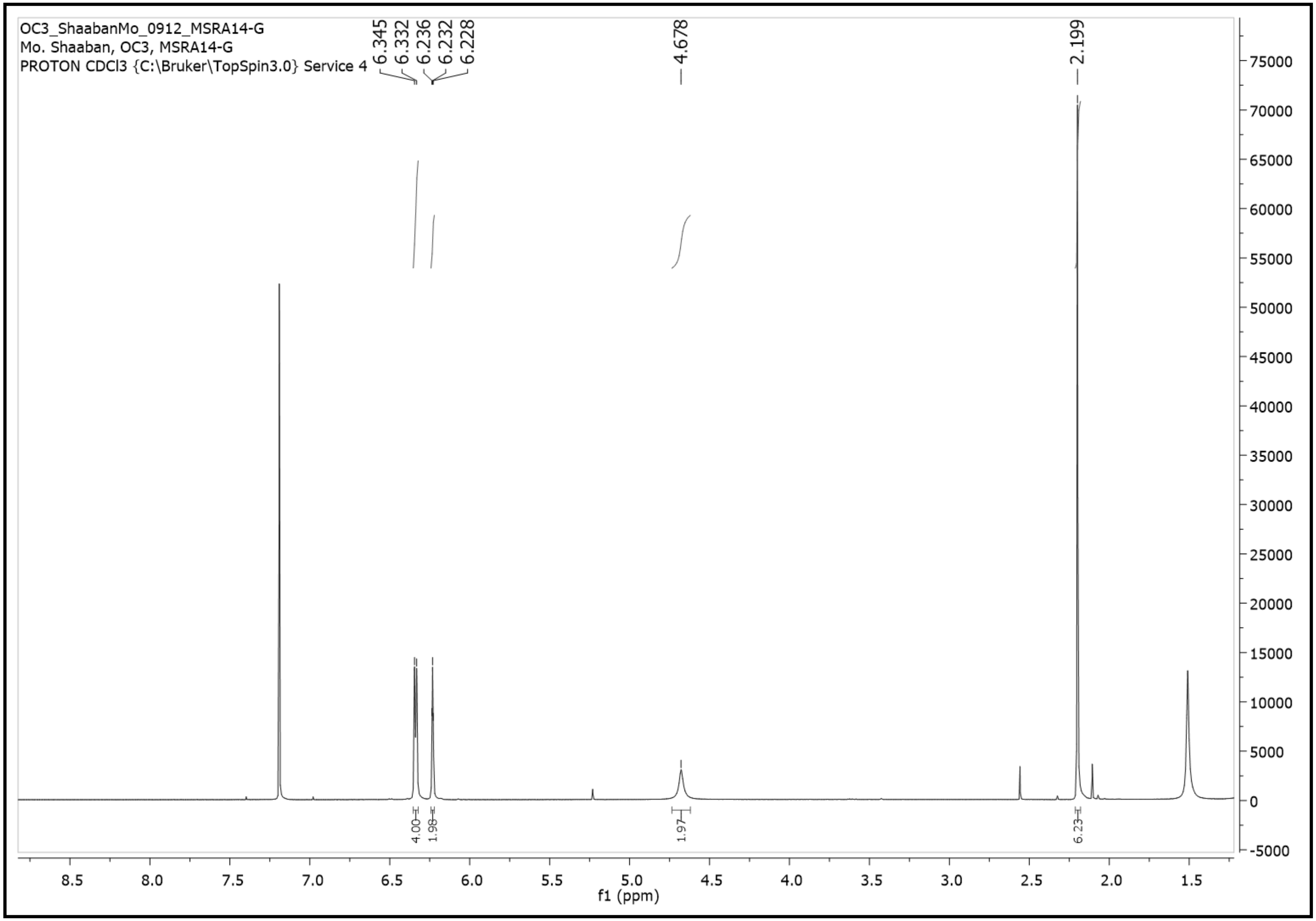

FIGURE S40:^{1}H NMR expansion of compound <u>7</u> ($CDCl_3$-*d*, 500 MHz)

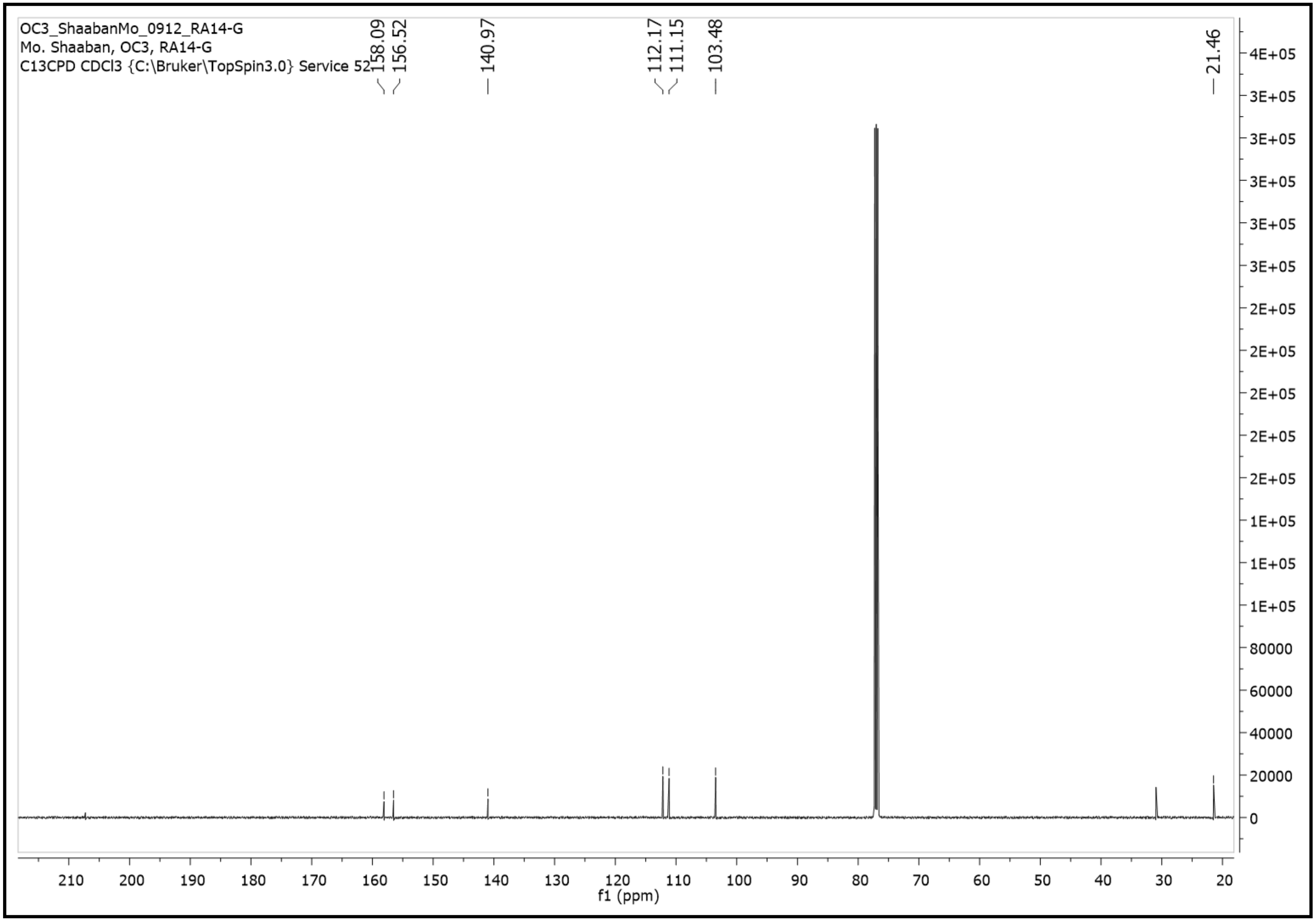

FIGURE S41: ^{13}C NMR spectrum of compound 7 ($CDCl_3$-*d*, 125 MHz)

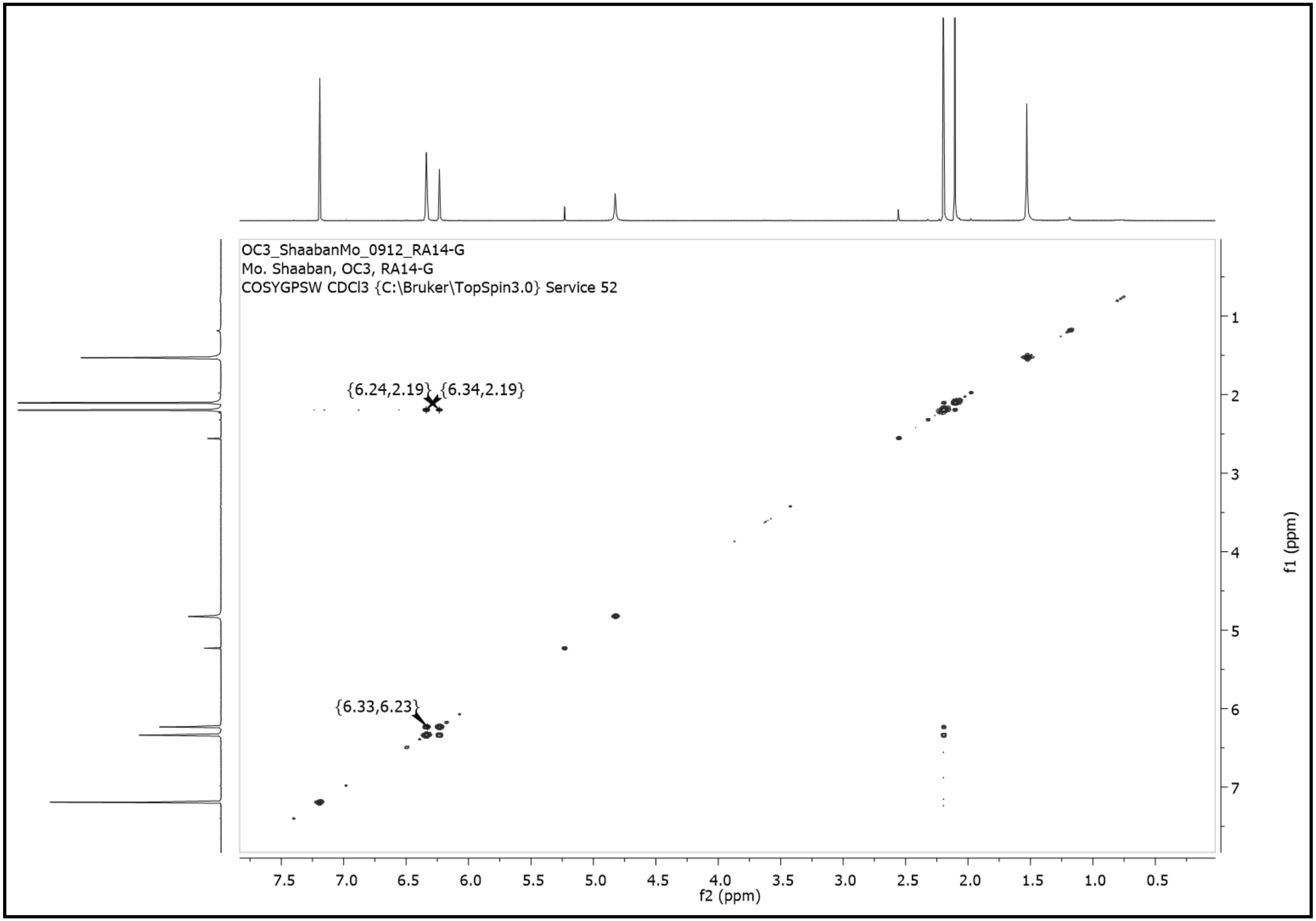

FIGURE S42: H-H COSY spectrum of compound 7 ($CDCl_3$-*d*, 500 MHz)

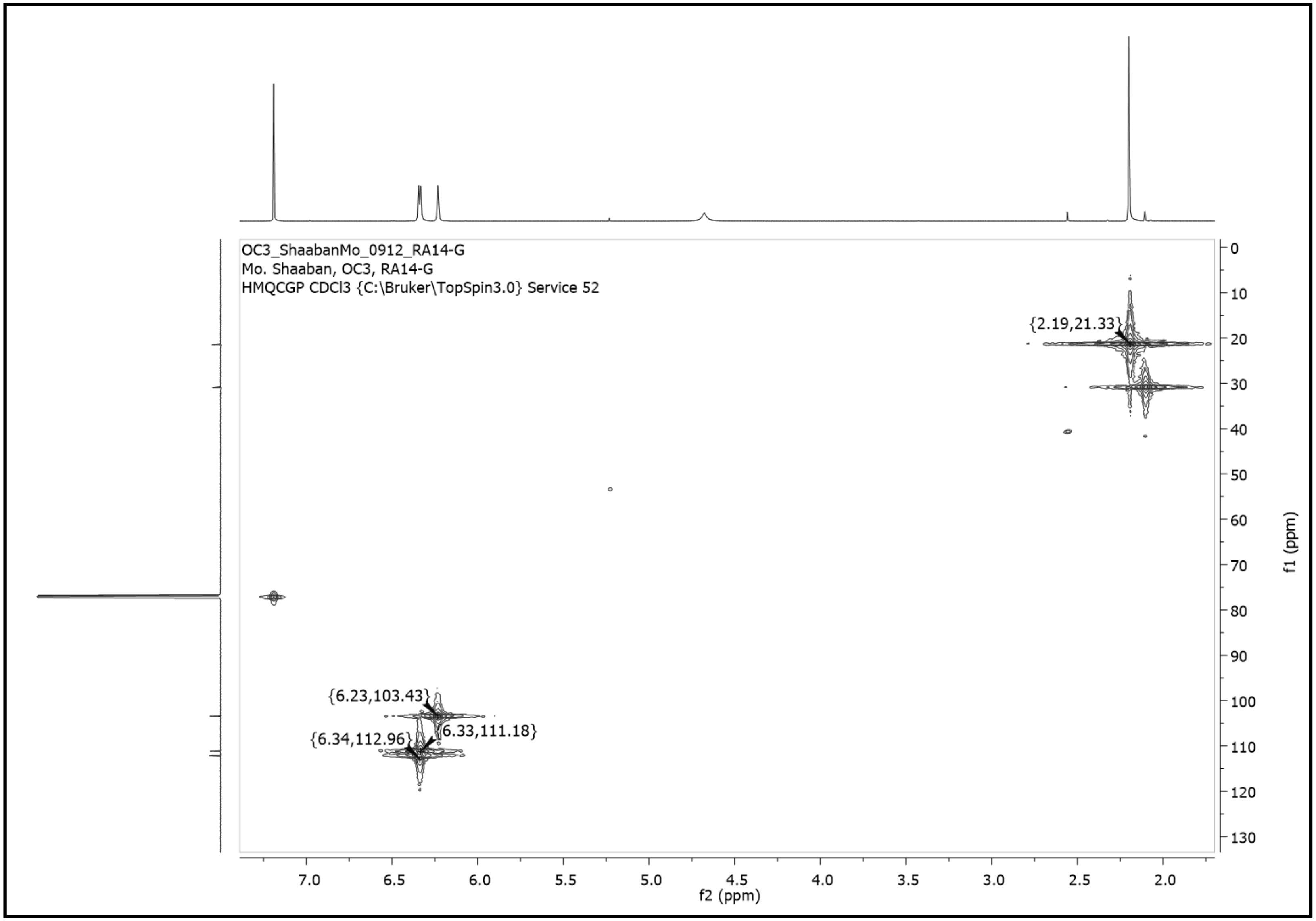

FIGURE S43: HMQC spectrum of compound 7 ($CDCl_3$-*d*, 500 MHz)

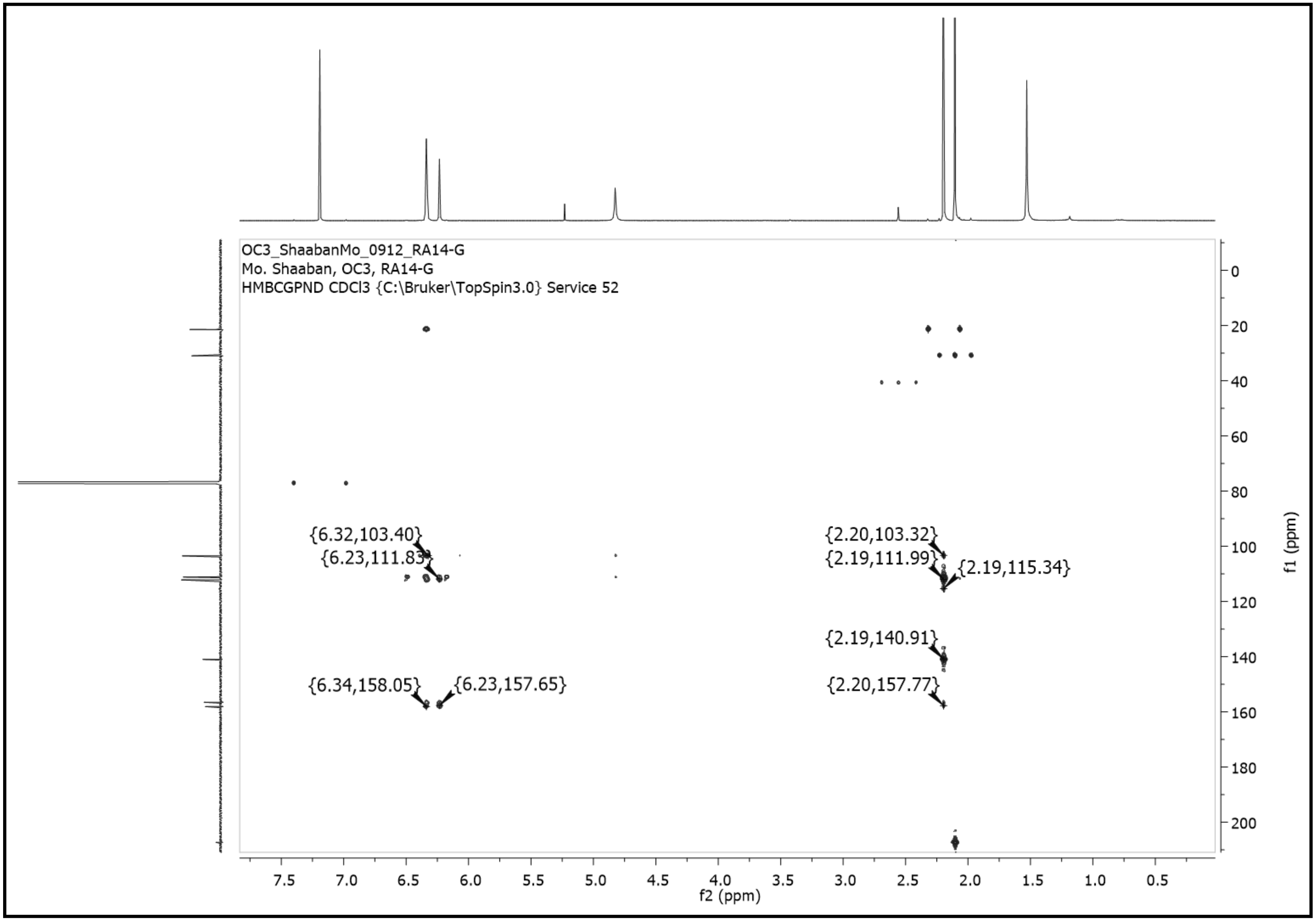

FIGURE S44: HMBC spectrum of compound 7 ($CDCl_3$-*d*, 500 MHz)

Center for DRUG DISCOVERY RESEARCH and DEVELOPMENT
Openlynx Report -
Sample: 60
File:A41020
Description:C-7

Vial:1:F,4
Date:16-Dec-2019

ID:
Time:11:54:2[illegible]

Printed: Mon Dec 16 12:16:06 2019

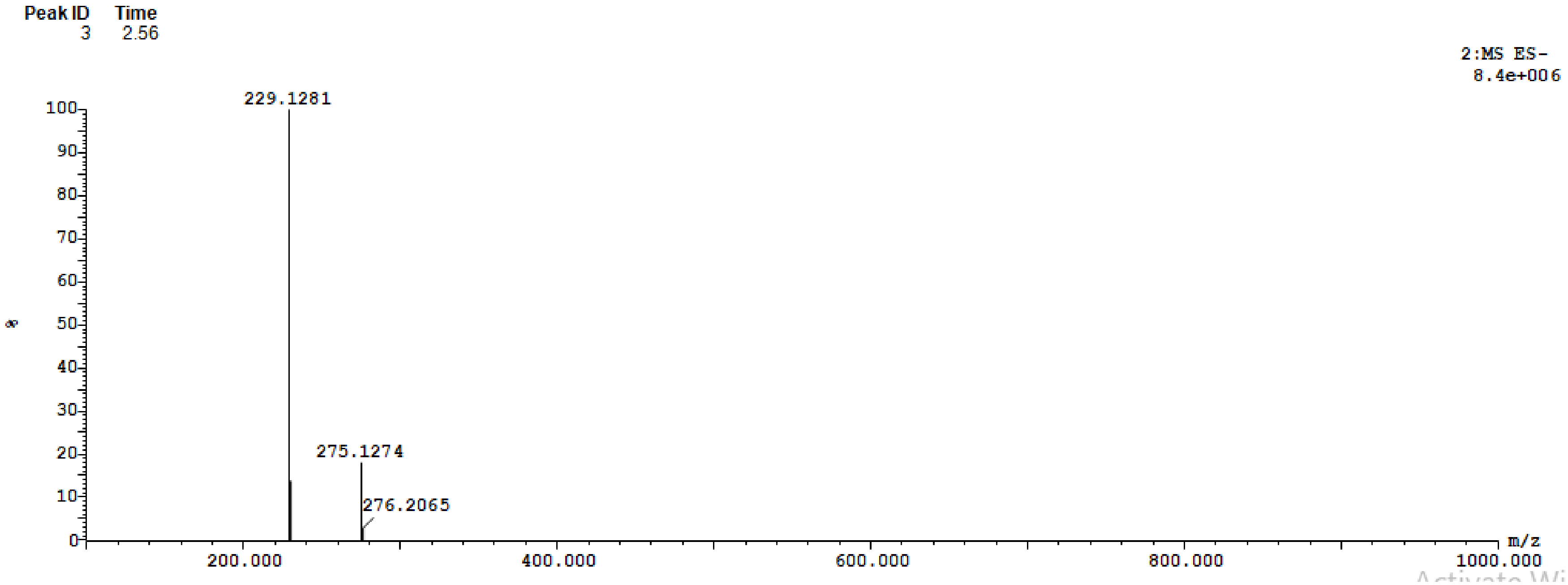

FIGURE S45: Negative ESI-MS spectrum of compound 7

Center for DRUG DISCOVERY RESEARCH and DEVELOPMENT
Openlynx Report -

Sample: 60	Vial:1:F,4	ID:
File:A41020	Date:16-Dec-2019	Time:11:54:2[illegible]
Description:C-7		

Printed: Mon Dec 16 12:16:06 2019

Peak ID	Time
3	2.56

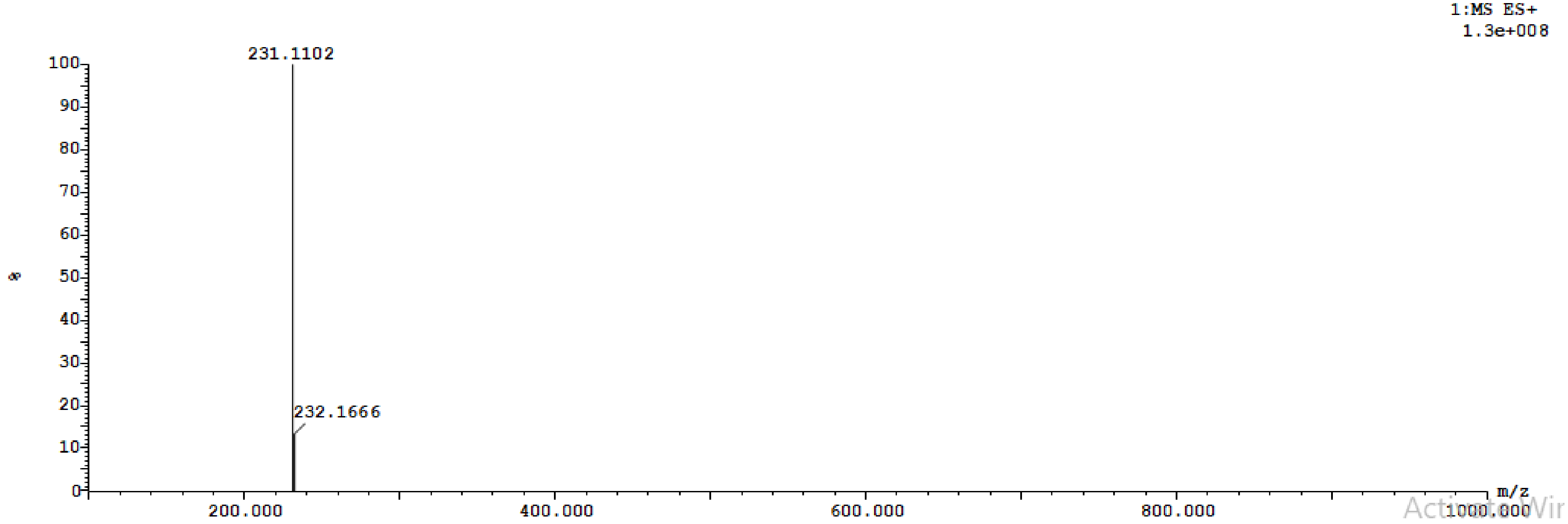

Figure 46:Positive ESI-MS spectrum of compound 7

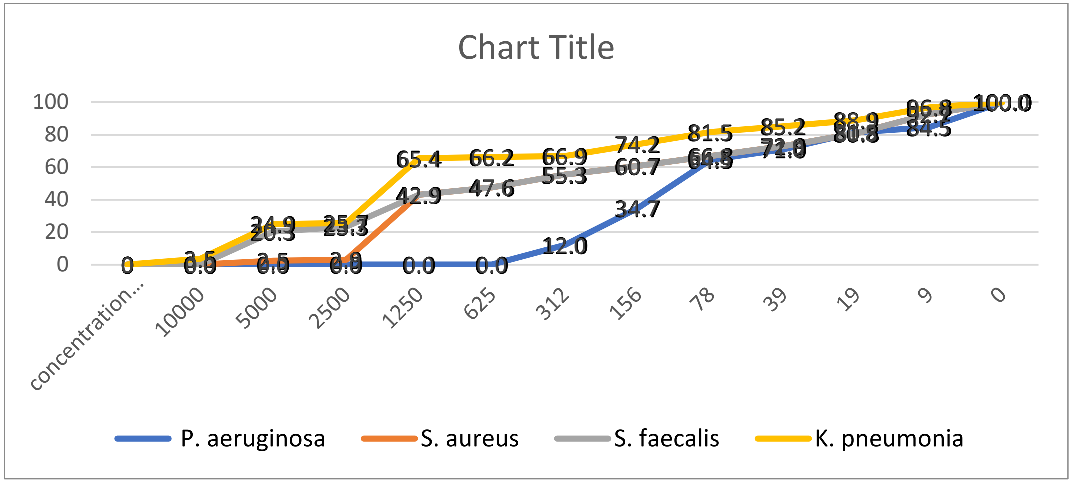
Chart Title
100
80
60
40
20
0
concentration...
10000
5000
2500
1250
625
312
156
78
39
19
9
0
P. aeruginosa
S. aureus
S. faecalis
K. pneumonia

(A)

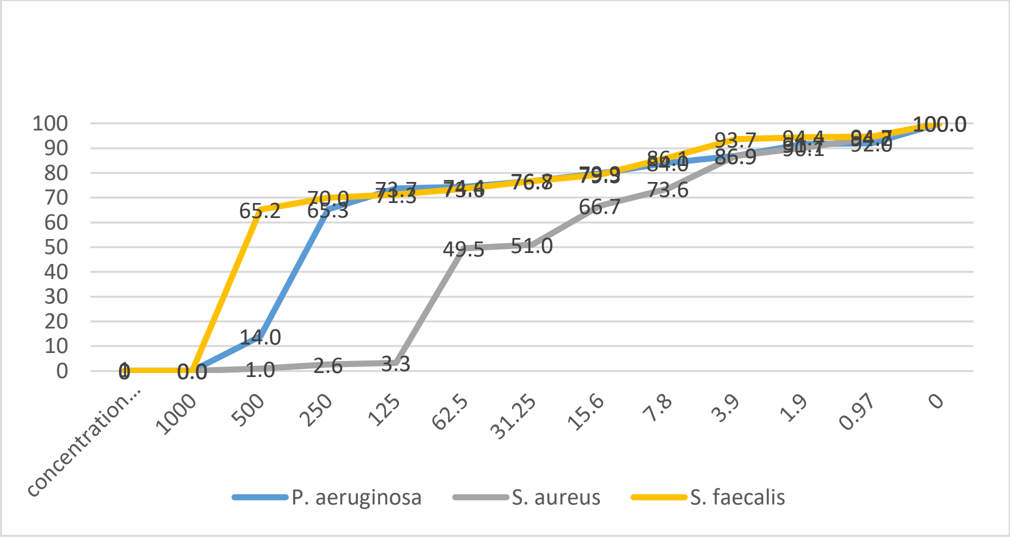
100
90
80
70
60
50
40
30
20
10
0
concentration...
1000
500
250
125
62.5
31.25
15.6
7.8
3.9
1.9
0.97
0
P. aeruginosa
S. aureus
S. faecalis

(B)

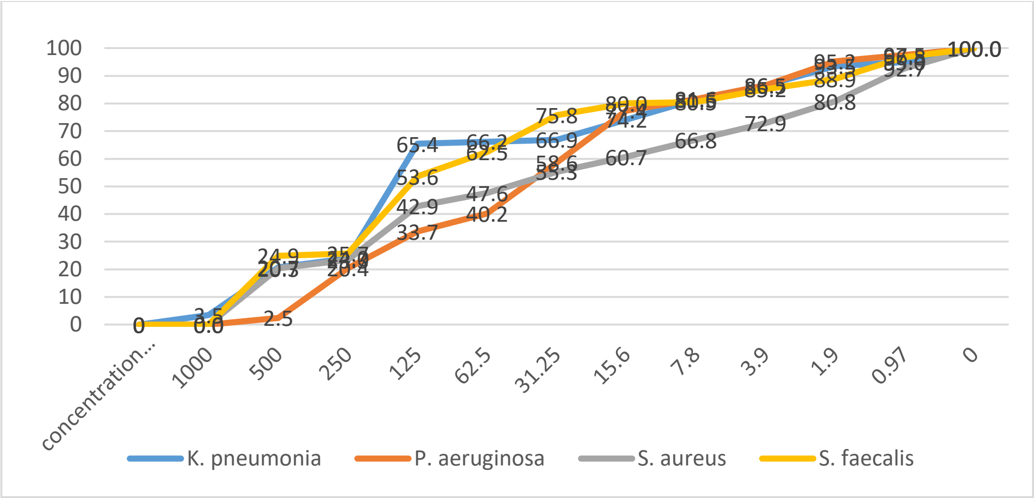
100
90
80
70
60
50
40
30
20
10
0
concentration...
1000
500
250
125
62.5
31.25
15.6
7.8
3.9
1.9
0.97
0
K. pneumonia
P. aeruginosa
S. aureus
S. faecalis

(C)

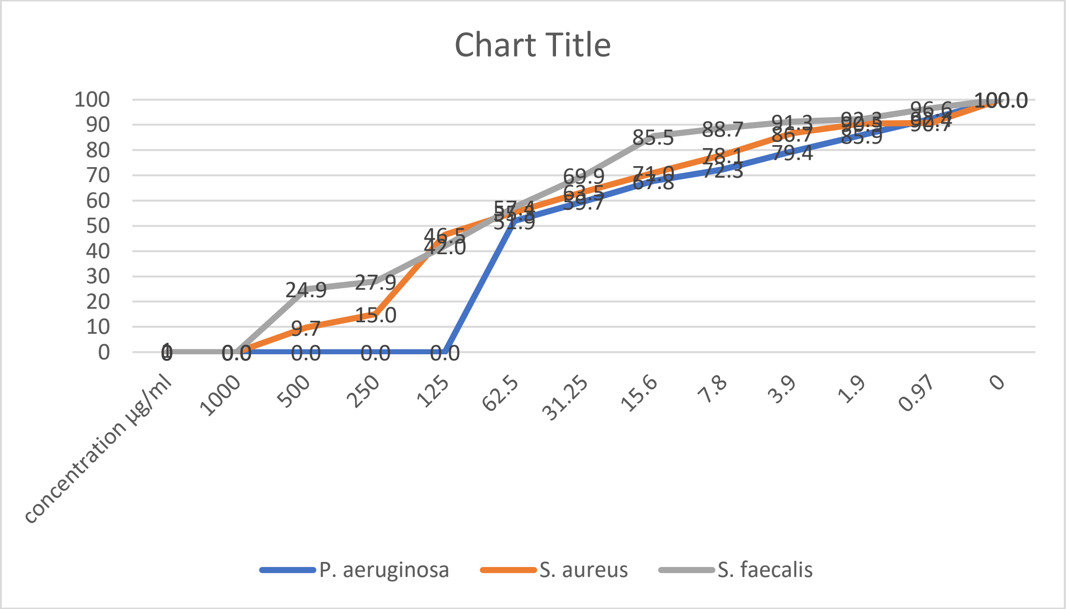
Chart Title
100
90
80
70
60
50
40
30
20
10
0
concentration µg/ml
1000
500
250
125
62.5
31.25
15.6
7.8
3.9
1.9
0.97
0
P. aeruginosa
S. aureus
S. faecalis

(D)

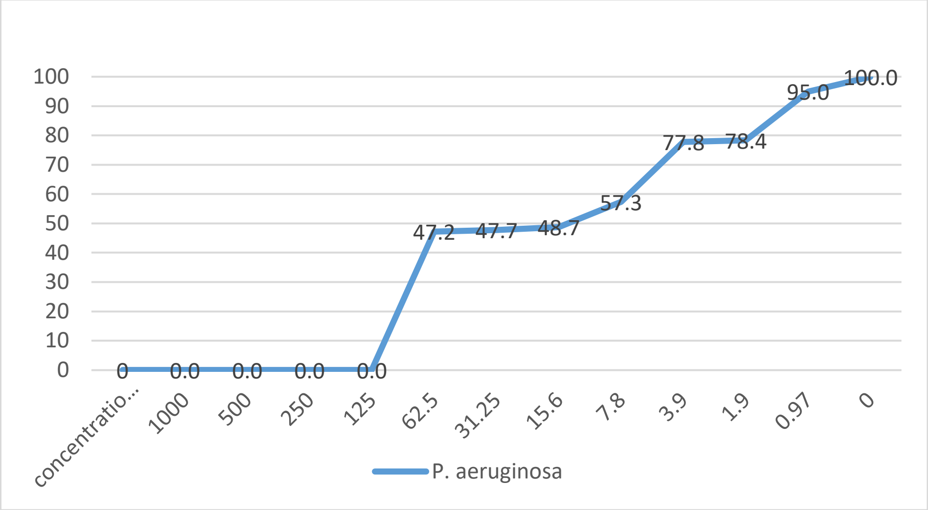

(E)

FIGURE S47:: Dose response curve for the effect of the EtOAC extract (A) and tested compounds 1 (B), 3(C), 4(D), 5(E) in broth microdilution assay against most susceptible reference microbial strains.
