# Supplementary material for: Cytotoxicity, Antimicrobial, and In Silico Studies of Secondary Metabolites From Aspergillus sp. Isolated From Tecoma stans (L.) Juss. Ex Kunth Leaves
Source: Front Chem. 2021 Oct 13;9:760083. doi: 10.3389/fchem.2021.760083 (PMC8548774; doi:10.3389/fchem.2021.760083)
Supplement: Supplementary file 3 [file DataSheet1.docx]

**Supplementary data**

**Detailed discussion for the structure elucidation of compounds 1, 3-7**

**Arugosin C (1)**

It was expected that compound **1** is related to hydroxylated and/or methoxylated xanthones based on its chromatographic properties (Zhu and Lin, 2007). The ^1^H NMR data showed two singlet signals at δ_H_ 13.80 and 10.63 ascribable to the exchangeable protons of OH-12 and 3, respectively. The presence of two aromatic rings (A, C) was confirmed by the presence of three aromatic protons, two of which are *ortho* coupled for H-9 (δ_H_ 6.34) and H-10 (δ_H_ 7.21) of ring-A, while a singlet signal for H-4 (δ_H_ 6.74) at ring C. In addition, the tetrahydropyran scaffold (ring D) was established from the doublet signal of the oxygenated- *sp*^3^ methine proton resonates at δ_H_ 5.01 (H-25), doublet or triplet signal at δ_H_ 2.30 for a non-oxygenated H-20 attached to *sp*^3^ methylene group (CH_2_-19, δ_H_ 4.30 and 4.13). Moreover, it was expected that **1** contains a prenyl group from the presence of doublet signal at 3.24 ppm (CH_2_-14), multiplet one at 5.25 ppm for CH-15, and two singlets at 1.69 and 1.65 ppm corresponding to C-17 and C-18 methyl groups. As well as the spectrum displayed three singlet signals resonate at δ_H_ 1.25, 1.19, and 2.17 for the methyl groups at C-22, 23, and 24, respectively. The HMQC data revealed that all non-exchangeable protons displayed a direct correlation to their carbon atoms. On the other hand, the integrity of ring A was confirmed from the ^1^H-^1^H COSY spectrum which declared the off-diagonal correlation between H-9 and H-10. As well as, from the HMBC data, correlations between H-9 and C-8 (159.1) through *^2^J* bond length were observed while, through *^3^J* with C-13 (112.6) and C-11(124.1), finally with C-1 (197.2) through ^4^*J* bonds. Similarly, H-10 displayed *^a 3^J* correlation with C-8 (159.1), C-12 (163.4), and C-14 (27.7). Interestingly, the substitution pattern of ring C is confirmed by the correlation between H-4 and C-6 (145.3), C-3 (155.6), C-2 (119.7), C-1 (197.2), and C-24 (16.6). On the other side, the ^1^H-^1^H COSY showed off-diagonal contiguous correlations of the methylene protons at position 19 with each other, and with methines at positions 20 and 25. As well as, the HMBC spectrum displayed a correlation between H-19 and C-20 (49.3), C-6 (145.3), and C-25 (74.1); also between H-25 (δ_H_ 5.01) and C-19 (65.2), C-20 (49.4), C-6 (145.3), C-7 (120.6), and C-8 (159.1). In all support the scaffold of ring D. The presence of the prenyl group was deduced from the HMBC correlation of CH_2_-14 with H-15 as well as the correlations between it with C-15 (121.8) and C-16 (133.3) and that between CH_3_-17 and CH_3_-18 with C-15 (121.8) and C-16 (133.3). Besides, its position at C-11 is due to the correlations between CH_2_-14 and C-10 (137.7), C-11 (124.1), and C-12 (163.4). In addition, the isopropyl substitution at position 20 was confirmed by the correlation between CH_3_-22 and 23 with C-21 (71.2) and their vicinal C-20. Also, the correlation between H-25, H-20, and H-19 with the quaternary C-21 supported the predicted framework. Finally, CH_3_-24 was attached to C-5 through its correlation with C-4 (120.2), C-5 (136.5), and C-6 (145.3) from the HMBC spectrum. Positive mode LR-ESI/MS spectrum finally confirmed the structure of **1** as arugosin C (Ballantine et al., 1973) by displaying a base peak at 447.3 corresponding to the adduct ion [M+Na] ^+^.

**Isoemericellin (3)**

It is related to the xanthone derivative based on its chromatographic and spectroscopic data**.** ^1^H NMR of **3** confirms the presence of rings A and B of a xanthone nucleus, deduced from the presence of two *ortho* coupling aromatic protons H-2 and H-1 of ring A at δ_H_ 7.41 and 6.79, respectively, together with singlet signal of H-12 for ring B at δ_H_ 7.22. Moreover, it was expected that **3** contains two prenyl groups, the 1^st^ one linked to the xanthone nucleus *via* ether linkage and distinguished by doublet signal of CH_2_-16 (δ_H_ 4.38), one multiplet for CH-17 (*δ*_H_ 5.55) together with two singlet signals for CH_3_-19 (δ_H_ 1.65) and 20 (δ_H_ 1.75). While, the 2^nd^ one was confirmed by doublet signal of CH_2_-21 (δ_H_ 3.33), one multiplet signal for CH-22 (δ_H_ 5.27) and singlet signals for CH_3_-25 (δ_H_ 1.70) and 24 (δ_H_ 1.68). Moreover, the ^1^H NMR showed two singlet signals for the methylene protons attached to the oxygen-bearing carbon (CH_2_-14) and CH_3_-15 at δ_H_ 5.01 and 2.39, respectively. The ^13^C-NMR, DEPT-135, and HMQC displayed five methine carbons δ_C_ 105.7 (C-1), 136.9 (C-2), 119.4 (C-12), 119.6 (C-17), and 121.8 (C-22), three methylenes *δ*_C_ 57.2 (C-14), 72.2 (C-16), and 27.1 (C-21); five methyl carbons δ_C_ 17.7 (C-15),18.1 (C-19), 25.9 (C-20), 17.8 (C-24), and 25.8 (C-25) and twelve quaternary among which the carbon signal at *δ*_C_ 184.7 (C-7) characteristic for xanthone nucleus ([Bringmann](https://pubmed.ncbi.nlm.nih.gov/?term=Bringmann+G&cauthor_id=12770594) et al., 2003). The analysis of ^1^H-^1^H COSY and HMBC spectra, delineated the exchangeable proton at position-4 (*δ*_H_ 12.87) as it displayed correlations with C-3 (122.9), C-4 (159.1), and C-5 (108.3). Moreover, the substitution pattern of ring A is confirmed by the correlation between the H-1 and H-2 and its correlation with C-3 (δ_C_ 122.90) as well as that between H-2 and C-4 (δ_C_ 159.10). In addition, ring B was also confirmed from the correlation of H-12 with C-8 (δ_C_ 118.10), C-10 (δ_C_ 152.5), and C-13 (δ_C_ 154.20) through *^3^J* and *^2^J* bonds, respectively. The findings supported the xanthone nucleus of the compound. Furthermore ^1^H-^1^H COSY and HMBC confirm the presence of the 1^st^ prenyl group at C-10 by the correlation of CH_2_-16 with H-17 and its correlations with C-17 (δ_C_ 119.6), C-18 (δ_C_ 139.10), and C-10 (δ_C_ 152.5). Moreover, the 2^nd^ group was established at C-3 by the correlation of 2H-21 with H-22 and its correlation through *^3^J* with C-23 (δ_C_ 133.40), C-2 (δ_C_ 136.9), and C-4 (δ_C_ 159.1). The position of the methylene group (CH_2_-14) at C-9 was established by its correlation with C-9 (δ_C_ 134.1), C-8 (δ_C_ 118.1), and C-10 (δ_C_ 152.5). On the other hand, CH_3_-15 is correlated to C-10 (δ_C_ 152.5), C-11 (δ_C_ 142.6), and C-12 (δ_C_ 119.4) to confirm its position. Based on the aforementioned analysis and its positive ESI/MS which displayed a molecular ion peak at m/z: 431.2, as well as comparison with previously published data for related compound (Bringmann et al., 2003) **3** was identified as isoemericellin.

**Sterigmatocystin (4) and dehydrosterigmatocystin (5)**

As in the case of **3**, compounds **4** and **5** are expected to be hydroxylated and/or methoxylated xanthones (Zhu and Lin, 2007). The ^1^H NMR data of **4** and **5** (**Table 1**) showed a singlet signal at δ_H_ 13.18 and 13.20 for the hydrogen-bonded phenolic proton at C-3, respectively. Moreover, the trisubstituted aromatic ring A in both compounds was established by the presence of three signals displayed at ≈ δ_H_ 6.70/6.69 (dd, H-4), 7.41/7.43 (t-like, H-5), and 6.80/6.76 (dd, H-6), as well as the singlet signal for H-11 at ≈ δ_H_ 6.35/6.30 which support the presence of penta-substituted ring B which further confirmed the hydroxylated and/or methoxylated xanthone structures of **4** and **5**, respectively. Moreover, it was expected that **4** display a bisfuran ring due to the presence of two proton signals at δ_H_ 6.76 (H-14), 4.72 (H-15) together with olefinic protons at δ_H_ 5.40 (H-16), and 6.42 (H-17) which are replaced by two saturated methylene groups at δ_H_ 2.27 (H-16), 4.11 (H-17_a_), and 3.63 (H-17_b_) in **5** to establish the presence of bisdihydrofuran ring in **5** instead of bisfuran ring in **4**. Moreover, both compounds showed a singlet signal at ≈ 3.9 for an OCH_3_ group. The ^13^C NMR of both compounds (**Table 1**) showed thirteen carbon resonances among which the carbonyl carbon at ≈ *δ*_C_ 181 is characteristic for xanthone skeleton (Zhu and Lin, 2007) with one methoxy signal. In addition to four characteristic carbons for the bisfuran ring in **4** especially the olefinic CH-16 (102.5) and CH-17 (145.3), which were replaced by two methylenes in the bisdihydrofuran ring of **5** resonate at 31.4 (CH_2_-16) and 67.9 (CH_2_-17). The COSY and HMBC data support the presence of ring A in both compounds by the off-diagonal sequential correlation of H-5 with H-4 and H-6 as well as the cross-peak of H-4 with C-2, C-3, and C-7; H-5 with C-3 and C-7, further between H-6 and C-1, C-2, C-4, and C-7. Moreover, ring B core structure was established based on the correlation of H-11 and C-12, C-13, C-1, C-8, C-9, and C-10. Further, the complete consistency of the bisfurane ring in **4** was supported from the ^1^H-^1^H off-diagonal correlation between H-15, H-14, and H-16 and that between H-16, H-15 and H-17. As well as, from the HMBC data, H-14 was coupled to C-9 (106.5), C-10 (164.7), C-15 (48.3), C-16 (102.5), and C-17 (145.3), while H-15 with C-9, C-10, C-14, C-16, and C-17. Also, H-17 displayed long and short-range correlations between C-14, C-15, and C-16. On the other hand, the dihydrobisfuran ring in **5** was confirmed by correlation of H-15 with vicinal protons at positions 14 and 16; H-16 with H-15 and H-17 as well as the methylene protons at C-17 showed cross-peaks with each other and with H-16. In addition, the correlation between H-14 and C-10 (166.2), C-11 (89.8), C-15 (44.3), C-16 (31.4), and C-17 (67.9); H-15 and C-14 (113.5); H-17 and C-15 (44.3) as well as that of H-16 with C-9 (105.8), C-14 (113.5), and C-15 (44.3). The location of the methoxy group at position 12 in both compounds was established from the HMBC correlation with C-11and C-12, while the hydroxyl group was assigned at position 3 as it is correlated with C-2, C-3, C-4, and C-5. Final confirmation of **4** and **5** was obtained from the positive ESI/MS which displayed molecular ion peak at *m/z* 347.1 [M+Na]^+^ for **4** which is two a.m.u. less than that of **5** *m/z* 349 [M + Na]^+^. Accordingly, **4** was identified as sterigmatocystin (Davies et al., 1960), while **5** is dihydrosterigmatocystin (Gorst-Allman et al., 1977; Zhu and Lin, 2007).

**Versicolorin B (6)**

The chromatographic properties and spectroscopic data of **6** suggest that it possesses anthraquinone chromophore (Hawas et al., 2012). The anthraquinone skeleton was established from the presence of three aromatic protons in ^1^H NMR. One is displayed as a singlet signal at δ_H_ 7.17 in addition to two *meta*-coupled protons shown at δ_H_ 7.18 and 6.54. Together with fourteen carbon signals, in ^13^C NMR spectrum, among which the key signals are for C-9 and C-10 at *δ*_C_ 192.9 and 181.8, respectively (Cole and Cox, 1981). In addition, it was expected that **6** contains bisdihydrofuran ring which also supported from the ^1^H NMR spectrum by the presence of di-oxymethine proton H-11 (δ_H_ 6.48), H-12 (δ_H_ 4.14), 2H-13 (δ_H_ 2.27 and 2.31), oxymethylene protons at position 14 (δ_H_ 4.13 and 3.60) in addition to their corresponding carbon signals at δ_C_ 113.3, 43.9, 30.2, and 67.1, respectively. Further confirmation of **6** was obtained from ^1^H-^1^H COSY and HMBC data which evidence the anthraquinone moiety through the off-diagonal correlation between H-5 and H-7 as well as the long-range coupling between H-5/C-7 (107.8), H-5/C-10 (181.8), H-7/C-5 (109.2), H-7/C-8a (108.0) in ring C and H-4/C-2 (120.0), H-4/C-9a (110.9) in ring A. Furthermore, bisdihydrofuran ring was established from the off-diagonal correlations which confirm the connectivity of H-11 with H-12 and H-13 with H-12 and H-14. The HMBC data confirmed this finding through the various cross-peaks between H-11 with C -2, C-3, C-12, C-13, C-14; *^2^J* bond correlation of H-12/C-2; H-14 through *^3^J* bond length with C-11 and C-12. In addition to the *^3^J* bond correlation of H-13 to C-2 and C-11. Finally, negative LR-ESI-MS exhibited a molecular ion peak at *m/z* 338.9 [M-H] ^-^ corresponding to the molecular formula C_18_H_12_O_7_. Accordingly, compound **6** was identified as versicolorin B or C (Cole and Cox, 1981; Jakšić et al., 2012). Interestingly, it was found that the optical activity of **6** is –1.5° which is indicative for its levorotatory form, ultimately established **6** as versicolorin B, since versicolorin C is optically inactive.

**Diorcinol (7)**

Chromatographic properties and UV data of **7** suggest that it may contain a simple aromatic chromophore (Itabashi et al., 1993). It was expected that compound **7** displays two identical molecules linked together due to the presence of a doublet signal integrated to four *meta*-coupled protons at δ_H_ 6.34 (4/4`, 6/6`) together with t-like signal integrated to two *meta*-coupled protons at δ_H_ 6.23 (2/2`). In addition to the singlet signal integrated for 6H at δ_H_ 2.20 ascribable to 7/7`-CH_3_ and the two protons at δ_H_ 4.68 characteristics for phenolic OH-3/3`. The ^13^C NMR data together with HMQC showed six methines correlated to their corresponding protons through *^1^J* bond and six quaternary carbons all displayed in the aromatic region in addition to two methyl groups. Moreover, the ^1^H-^1^H COSY and HMBC gave final confirmation of the chemical structure of **7**. Finally, ESI/MS support that compound **7** is a dimer of 3- hydroxyl, 5- methyl phenyl ether due to the presence of *m/z* at 231.1102 [M+H] ^+^) and 229.1232 [M- H]^-^ in positive and negative mode, respectively. Hence, **7**  was identified as 3, 3'-dihydroxy-5, 5'-dimethyl diphenyl ether, diorcinol (Itabashi et al., 1993).

**Detailed discussion of docking results on Hsp90 for compounds 1, 6, and 7**

**1** has high binding affinity at the catalytic interface with a docking score of -9.0 Kcal/mol with most of the interactions are hydrophobic. For example, Ring A displayed hydrophobic *pi*-stacking interaction with the alkyl residue of Ala-55. In addition, ring C showed *pi*-stacking with the amide functionality of the critical amino acid Asp-54. The methyl group at position 18 of the iso-pentene sidechain configure hydrophobic interaction with the methyl residue of Phe-138. Furthermore, compound **6** showed much-varied interaction with the key amino acids. Rings A and B displayed hydrophobic interaction with the methyl group of the Ala-55 residue, while a conventional hydrogen bonding was obvious between the hydroxyl group at position 6 in ring C and the carboxylic functionality of Asp-93. Also, *pi*-stacking was obvious between ring C and Asn-51, while Met-98 displayed hydrophobic interactions with rings A and B of the core structure. Also, an electrostatic hydrogen bonding was displayed between ring B and Thr-184 with a net result of the good binding energy of -8.9 Kcal/mol. Lastly, **7** displayed fair fitting within the kinase cleft with a binding energy of -7.5 Kcal/mol deduced from the *pi*-stacking interaction with the methyl group of Ala-55 and the sulfur atom of Met-98. Also, the methyl substitution of the first phenolic ring displayed hydrophobic interaction with both Phe-138 and Leu-107 amino acids.
